# *Chryseobacterium lacus* sp. nov. Isolated From the Surface Water of Two Lakes With Light-Induced Carotenoid Production

**DOI:** 10.3389/fmicb.2020.00251

**Published:** 2020-03-04

**Authors:** Jing Zhang, Cheng Gao, Xue-Mei Yu, He-Yuan Lun, Zong-Jun Du

**Affiliations:** ^1^Marine College, Shandong University, Weihai, China; ^2^State Key Laboratory of Microbial Technology, Shandong University, Qingdao, China

**Keywords:** *Chryseobacterium*, genome, transcriptome, carotenoid synthesis, light, surface water

## Abstract

Two Gram-stain-negative, rod-shaped, gliding, catalase-positive, and facultative anaerobic strains, YLOS41^T^ and XH07, were isolated from surface water of Yilong Lake and West Lake of Dali in Yunnan Province, respectively. Both strains were yellow-colored under light conditions and white-colored under dark conditions. The results of physiological and chemotaxonomic characterization, sequencing and phylogenetic analysis, and draft genome sequence comparison demonstrated that the two strains represented a single novel species within the genus *Chryseobacterium*, for which the name *Chryseobacterium lacus* sp. nov. is proposed. The type strain is YLOS41^T^ (= KCTC 62352^T^ = MCCC 1H00300^T^), and the second strain is XH07 (= KCTC 62993). During the cultivation process, we found that the colony color of the two strains changed from white to yellow with illumination. The study investigated the effects of light irradiation on the strain YLOS41^T^. Results showed that light irradiation did not affect the growth of cells but significantly increased carotenoid synthesis, which caused the change of colony color. In-depth metabolic analysis was conducted by transcriptome. The predominant changes were found for genes involved in carotenoid synthesis as protection from light damage. Based on the genome and transcriptome, we proved that strain YLOS41^T^ possessed a complete synthetic pathway of carotenoid and speculated that the production was zeaxanthin. This was the first report of *Chryseobacterium* species with light-induced carotenoid synthesis. This study enhances our present knowledge on how *Chryseobacterium* species isolated from surface water responds to light damage.

## Introduction

The genus *Chryseobacterium* belongs to the family *Flavobacteriaceae* and was emended from the genus *Flavobacterium* (Vandamme et al., 1994; Bernardet et al., 1996). In general, species of the genus *Chryseobacterium* are yellow-colored, rod-shaped, and non-spore-forming bacteria; they typically have menaquinone 6 as the sole respiratory quinone (Bernardet et al., 2006). Currently, the genus *Chryseobacterium* comprises 110 species with validly published names^1^, among which *Chryseobacterium gleum* is the type species (Vandamme et al., 1994). The members of the genus *Chryseobacterium* have been isolated from a wide variety of habitats including plants, soil, roots of trees, clinical specimens, animal bodies, freshwater, wastewater, compost, sludge, sediments, and dairy products (Tai et al., 2006; Cho et al., 2010; Montero-Calasanz et al., 2013; Xu et al., 2015; Yang et al., 2015; Zhao et al., 2015).

Light is an environmental stimulus that affects many living organisms, including prokaryotes. Bacteria in the visible region will be exposed to high light intensities, which means that transmission of large amounts of energy, if not controlled, can lead to accumulation of reactive oxygen species (ROS) in the cells (Muramatsu and Hihara, 2012). Photosynthetic bacteria have both non-enzymatic and enzymatic mechanisms to protect themselves against ROS (Latifi et al., 2009). Non-phototrophic bacteria use quenchers, such as carotenoids, which interact with an excited singlet oxygen itself to prevent damage of cellular molecules (Glaeser et al., 2011). Many bacteria have carotenoids in the visible light region. Some of these bacteria showed a difference in carotenoid synthesis under dark and light conditions. Regarding light-dependent physiological features, the study of light-induced carotenoid production is relatively more developed, while other photodependent phenotypes have not yet been extensively characterized (Takano et al., 2011). Light irradiation-induced synthesis of carotenoids means that these bacteria have light-regulating systems to control carotenoid biosynthesis, but the details of such a regulatory system are poorly known. Currently, the only clarified mechanisms of light-induced carotenoid synthesis are in *Myxococcus xanthus* and *Thermus thermophilus* (Pérez-Marín et al., 2008; Takano et al., 2011, 2014). Light-induced transcriptional regulators, CarA/LitR, were involved in carotenoid production in these bacteria. They are thought to be involved in light-induced transcriptional regulation in a wide variety of non-phototrophic bacteria, as the seventh photosensory protein family (Takano et al., 2011).

In this study, we report a novel *Chryseobacterium* species based on the polyphasic approach. It was observed that the colony colors of two strains were different in the dark or with illumination, and so we investigated the effects of light irradiation on the strain YLOS41^T^. Through pigment detection and genomic and transcriptomic analysis, we proved that light irradiation significantly increased carotenoid synthesis, causing the difference in colony color in the dark or with illumination. We explored the synthetic pathways for the synthesis of carotenoids and the genes that may be involved in light-induced regulation. To date, this is the first report of *Chryseobacterium* species with light-induced carotenoid synthesis, which increased our understanding of how *Chryseobacterium* species, isolated from surface water responds to light damage.

## Materials and Methods

### Organism, Growth, and Maintenance Conditions

The strain YLOS41^T^ was isolated from surface water from the Yilong Lake, Yunnan Province, China (102° 34′ 22.8″ E, 23° 40′ 4.8″ N), and strain XH07 was isolated from surface water from the West Lake of Dali, Yunnan Province, China (100° 3.0461’ E, 26° 0.8606’ N). These two strains were subsequently maintained and subcultivated on modified agar 2216 (0.5% peptone, 0.1% yeast extract, 2% agar, water) at 33°C. They were then stored at -80°C in sterile 15% (v/v) glycerol. Due to the lack of effect of light irradiation on the growth of cells, the strains were cultivated without extra supplemental illumination for physiological and chemotaxonomic analysis. Light treatment of the strain was carried out under the illumination system of the incubator.

### Morphological, Physiological, and Biochemical Analysis

The morphological characteristics of the strains were investigated after 24 h of incubation on modified agar 2216. The Gram reaction was examined using the bioMérieux Gram-stain kit according to the manufacturer’s instructions. Cell morphology was investigated using light microscopy (E600; Nikon) and transmission electron microscopy (Jem-1200; Jeol). Gliding motility was carried out according to the method of Bowman (2000). Growth at different temperatures was examined on modified agar 2216 at 4, 15, 20, 25, 28, 30, 33, 37, 40, 45, and 50°C. The pH range for growth was tested between pH 5.5 and 9.5 (in increments of 0.5 pH units) in modified broth 2216 at 33°C and then the OD_600_ was measured. Tolerance to NaCl was examined on modified agar 2216 in the presence of 0.0–4.0% (w/v) NaCl at intervals of 1.0%. Growth on modified agar 2216 under anaerobic (10% H_2_, 10% CO_2_, and 80% N_2_) and microaerobic (5% O_2_, 10% CO_2_, and 85% N_2_) conditions was examined after incubation for 14 days in an anaerobic jar.

Tests for oxidase, catalase, nitrate, and lipase (Tweens 20, 40, 60, and 80), and hydrolysis of casein, agar, alginate, cellulose, and starch were carried out according to Dong and Cai (2001). The presence of flexirubin-type pigments was carried out according to Reichenbach (1989). The presence of carotenoid pigments was extracted and detected by the method of Biebl et al. (2007). The substrate-oxidation profile tests were carried out using Biolog GEN III microplates. Carbohydrate fermentation tests were performed using API 50 CHB fermentation kits (bioMérieux) and tests for additional enzyme activities were carried out using API 20NE strips and the API ZYM system (bioMérieux). For phenotypic characterization, all tests were repeated at least twice.

### Sequencing and Phylogenetic Analyses

Amplification of the 16S rRNA gene was performed by PCR with two primers: 27F and 1492R (Liu et al., 2014). The amplified gene was ligated into the pMD18-T vector (Takara). Then, recombinant plasmids were replicated in *Escherichia coli* DH5α cells. The 16S rRNA gene sequences obtained were compared with closely related sequences using BlastN and EzBioCloud’s Identify services. Multilocus sequence analysis of strains YLOS41^T^ and XH07 (MLSA) was performed using the partial sequences of three housekeeping genes. The sequences of the housekeeping genes, *gyrB*, *rpoB*, and *rpoD*, were obtained from GenBank. Accession numbers of these sequences are listed in Supplementary Table S1. The order of the three housekeeping genes was *gyrB*-*rpoB*-*rpoD*. The length of the rearranged sequence was 5643 bp (Supplementary Material). Phylogenetic trees of the 16S rRNA gene and housekeeping genes were reconstructed with neighbor-joining (NJ) and maximum likelihood (ML) algorithms in the MEGA software, version 7.0 (Kumar et al., 2016), with a bootstrap of 1000 replications (Felsenstein, 1985).

### Random Amplification of Polymorphic DNA (RAPD) Fingerprinting

Random amplification of polymorphic DNA (RAPD-PCR) was carried out according to the method by Bernardet et al. (2005). The cellular DNA was obtained using the genomic DNA mini kit (TaKaRa Bio). Primers P1 (5′-CTGCTGGGAC-3′) and P2 (5′-CGCCCTGCCC-3′) were used in this study (Bernardet et al., 2005). The PCR cycling program was (1) one cycle of 94°C for 5 min; (2) 30 cycles of 94°C for 1 min, 36°C for 2 min, 72°C for 2 min; and (3) one cycle of 72°C for 5 min. The PCR products were electrophoresed in 1% agarose gel.

### Proteomic Fingerprinting

A single colony of two strains on the modified agar 2216 was resuspended in 300 μl of water and vortexed. Then, the suspension was added to 900 μl of ethanol, mixed, and centrifuged at 15,000 × *g* for 2 min. The supernatant was discarded and the cell pellet was added to 20 μl of 70% formic acid and mixed. Next, the suspension was added to 20 μl of acetonitrile, mixed, and centrifuged at 15,000 × *g* for 2 min. One microliter of supernatant was spotted on a 96-spot polished steel MALDI target plate and naturally dried. These spots were added to 1 μl of matrix solution (alpha-cyano-4-hydroxycinnamicacid) and naturally dried. The 96-spot was detected using MALDI-TOF MS (Bruker Daltonics). Bacterial mass spectrometry data were collected via FlexControl. The MS spectra were analyzed by using MALDI Biotyper 3 software.

### Genomic DNA Sequencing, Assembly, Annotation, and Genomes Comparison

Extraction and purification of genomic DNA were performed by a bacterial genomic DNA mini kit (TaKaRa Bio). The genomic DNA was sequenced using the Illumina Hiseq sequencing technology. The draft genome was assembled and genes were identified by the method described previously (Zerbino and Birney, 2008; Hyatt et al., 2010). Genes were annotated using NCBI, KEGG, and COG databases. The gene clusters of secondary metabolites were identified by antiSMASH program (Blin et al., 2017). The DNA G + C content was determined according to the draft genome. The average nucleotide identity (ANI) was calculated using the BLAST (ANIb) and MUMmer (ANIm) algorithms (Richter and Rosselló-Móra, 2009). DNA–DNA hybridization (DDH) was calculated according to the method described by Meier-Kolthoff et al. (2013).

### Chemotaxonomic Analysis

For chemotaxonomic analyses, the biomass of two novel strains and related type strains, *Chryseobacterium taklimakanense* CCTCC AB 208154^T^ (presently the formally named species that shows the highest 16S rRNA gene similarity and the closest phylogenetic relationship with the novel species) and *Chryseobacterium gleum* JCM 2410^T^ (the type species of the genus *Chryseobacterium*), was harvested in the late stage of the exponential growth phase. Fatty acids were extracted and analyzed according to the method described by Sasser (1990). Polar lipids analysis was carried out according to Tindall et al. (2007).

### RNA Isolation, Library Preparation, cDNA Sequencing, and Bioinformatics

The biomass of the strain YLOS41^T^ was harvested in the medium stage of the exponential growth phase. The experimental group was under light conditions. The control group was under dark conditions. There were three parallel experiments in each group. For the extraction of total RNA, the Invitrogen TRIZOL^®^ Reagent Kit was used. The RNA quality was evaluated by test concentration (Qubit^®^ RNA Assay Kit), purity (NanoPhotometer^®^ spectrophotometer), integrity (RNA Nano 6000 Assay Kit of the Bioanalyzer 2100 system), and agarose gel electrophoresis. Thereafter, library preparation and cDNA sequencing were carried out by Novogene (Tianjin, China). The transcriptome library was constructed using the NEBNext^®^ Ultra^TM^ Directional RNA Library Prep Kit (Illumina) and cDNA was sequenced using Illumina Hiseq^TM^2500.

The raw data in fastq format were first processed through in-house perl scripts. In this step, clean data (clean reads) were obtained by removing reads containing adapter, reads containing ploy-N, and low-quality reads from raw data. Reads mapping to the reference genome was performed using Bowtie2-2.2.3 (Langmead and Salzberg, 2012). Quantification of gene expression was performed using HTSeq v0.6.1 (Trapnell et al., 2009). For DESeq with biological replicates, differential expression analysis was carried out using the DESeq R package (v1.18.0). Genes with an adjusted *P* value (padj) < 0.05 found by DESeq were assigned as differentially expressed (Anders and Huber, 2013). The sequence data were submitted to NCBI via the Sequence Read Archive (SRA) and are available under the BioProject ID: PRJNA577976. The SRA accession numbers were SRR10343458, SRR10343457, SRR10343456, SRR10343455, SRR10343454, and SRR10343453.

## Results

### Morphological, Cultural, Physiological, and Biochemical Characteristics

**FIGURE 1:**
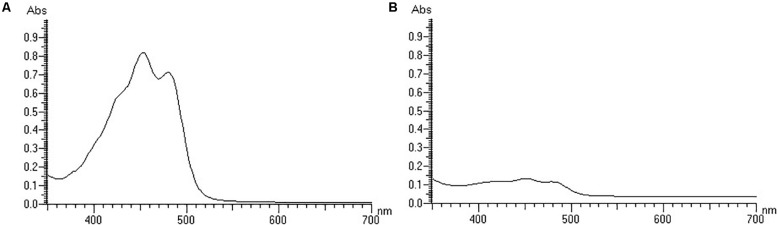
The UV–vis spectra of the carotenoid extracts of the strain YLOS41^T^ under light **(A)** or dark **(B)** conditions.

**FIGURE 2:**
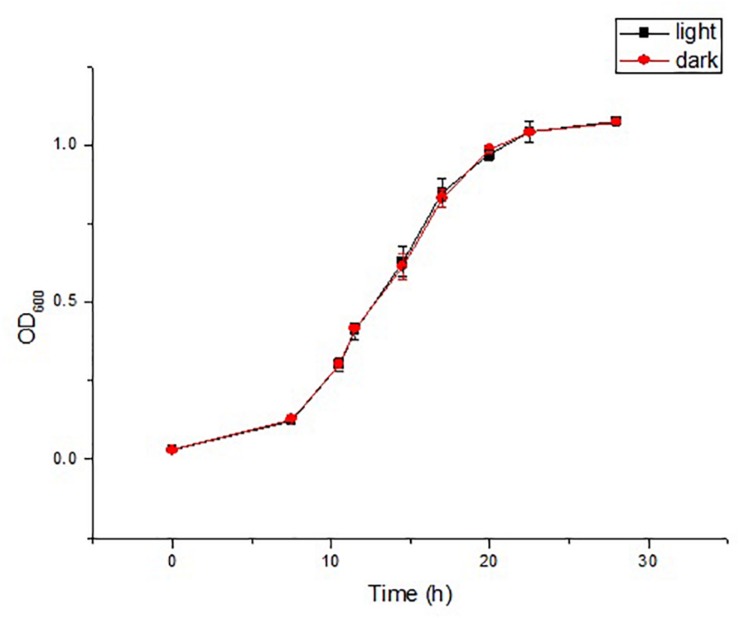
Time courses of cell growth (OD_600_) in the strain YLOS41^T^ under dark or light conditions (*n* = 3).

Irrespective of the cultivation time, both strains were yellow-colored under light conditions and white-colored under dark conditions. White-colored colonies slowly turned yellow after a period of light treatment, but the color did not change without light treatment. The strains were first cultured under light conditions, and the grown colonies were yellow, and then cultured under dark conditions, and the newly grown colonies were white (Supplementary Figure S1). The above results indicated that light irradiation could stimulate the colony color to turn yellow, and that the color did not change without light irradiation. Then, the pigments were detected. The isolates did not produce flexirubin-type pigments neither in light treatment nor in dark treatment. For the detection of carotenoid pigments, strain YLOS41^T^ presented UV–vis absorption peaks at 421, 452, and 478nm in the light treatment, but not in the dark treatment (Figure 1). The result meant that there was carotenoid synthesis under light cultivation and not under dark cultivation. Therefore, the difference in colony color under light and dark conditions was caused by the presence of carotenoids. Then, we investigated the effect of light irradiation on cell growth. Results showed that light irradiation did not affect the growth of cells (Figure 2).

Both strains were Gram-stain-negative and rod-shaped. The cells of these two strains were 0.5–0.7 μm in width and 0.8–2.6 μm in length (Supplementary Figure S2). The strains were able to grow at 4–40°C (optimum 33°C), at pH 6.5–9.0 (optimum pH 7.0–8.0), and at 0.0–2.0% (w/v) NaCl (optimum 0.0%). Growth of the isolates occurred in anaerobic and microaerobic conditions. Both strains have no flagella. Gliding motility was observed. The detailed morphological, physiological, and biochemical characteristics were provided in the species description, and the features that distinguish the two novel strains from their closely related species are listed in Table 1.

**TABLE 1:** Differential phenotypic characteristics of the novel strains and related species.

**Characteristics**	**1**	**2**	**3**	**4**
Flexirubin-type pigments	−	−	−	+
Citrate utilization	−	−	−	+
**Hydrolysis of**				
Tween 20	+	+	−	+
Tween 80	+	+	−	+
**Enzyme activities**				
Tryptophan deaminase	−	−	+	−
Urease	−	−	−	+
Arginine dihydrolase	−	−	−	+
α-Chymotrypsin	−	w	w	−
α-Glucosidase	−	−	+	+
β-Glucosidase	−	−	−	+
*N*-acetyl-β-glucosaminidase	−	−	−	+
Acid from				
L-Arabinose	−	−	−	+
D-Fructose	−	−	−	+
D-Mannose	−	−	w	+
D-Maltose	−	−	+	+
Starch	−	−	+	+
Glycogen	−	−	+	+
Potassium 5-ketogluconate	+	+	w	−
**Oxidation of**				
Dextrin	−	−	+	+
D-Maltose	−	−	+	+
D-Cellobiose	−	−	+	+
Gentiobiose	−	−	+	+
D-Turanose	−	−	−	+
D-Raffinose	+	−	−	−
3-Methyl glucose	−	−	+	+
D-Glucose-6-PO_4_	−	−	+	+
D-Fructose-6-PO_4_	−	+	−	+
L-Arginine	+	+	−	+
L-Aspartic acid	−	−	+	+
L-Glutamic acid	−	−	+	+
L-Histidine	+	−	−	−
L-Lactic acid	−	−	+	−
Formic acid	−	−	−	+

*Strains: 1, *C. lacus* sp. nov. YLOS41^*T*^; 2, *C. lacus* XH07; 3, *C. taklimakanense* CCTCC AB 208154^*T*^; 4, *C. gleum* JCM 2410^*T*^. All data were obtained in this study. All strains are positive for indole production; activity of catalase, gelatinase, alkaline phosphatase, esterase (C4), esterase lipase (C8), leucine arylamidase, valine arylamidase, cystine arylamidase, acid phosphatase, and naphthol-AS-BI-phosphohydrolase; oxidation of gelatin, glycyl-L-proline, Tween 40, acetoacetic acid, and acetic acid; acid production from D-glucose and aesculin ferric citrate. All strains are negative for activity of oxidase, *o*-nitrophenyl-β-D-galactopyranoside, lysine decarboxylase, ornithine decarboxylase, lipase (C14), trypsin, α-galactosidase, β-galactosidase, β-glucuronidase, α-mannosidase, and α-fucosidase; oxidation of sucrose, stachyose, D-lactose, inosine, D-sorbitol, D-mannitol, D-arabitol, D-aspartic acid, D-serine, L-alanine, L-pyroglutamic acid, pectin, L-malic acid, α-keto-butyric acid, and propionic acid; acid production from D-cellobiose, sucrose, inulin, D-melezitose, D-raffinose, D-xylitol, D-turanose, D-lyxose, D-fucose, L-fucose, potassium gluconate, and potassium 2-ketogluconate. +, positive reaction; −, negative reaction; w, weakly positive reaction.*

### Molecular Phylogenetic Analysis

**FIGURE 3:**
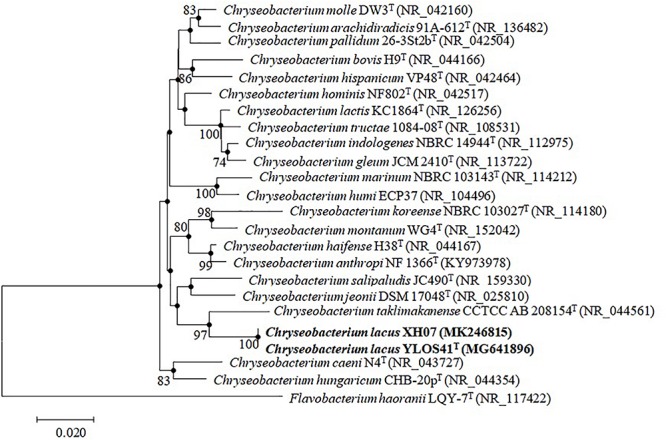
Phylogenetic tree constructed with 16S rRNA gene by the NJ algorithm, showing the position of *C. lacus* sp. nov. YLOS41^T^ and *C. lacus* XH07 among related taxa. Bootstrap values (expressed as percentages of 1000 replications) greater than 70% are shown at the nodes. *Flavobacterium haoranii* LQY-7^T^ was used as an outgroup. Filled circles indicate that the corresponding nodes were also recovered in the ML algorithm. Bar, 0.020 substitutions per nucleotide position.

**FIGURE 4:**
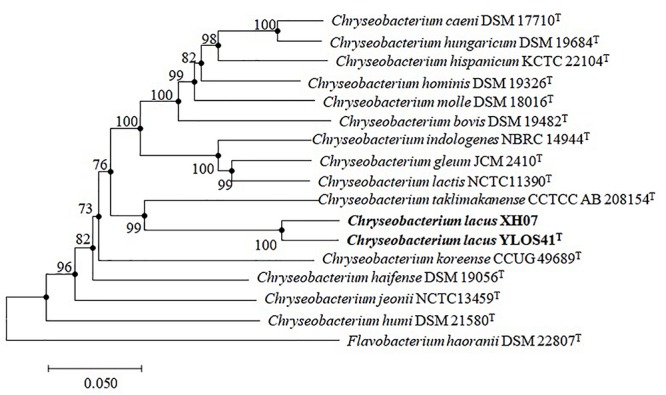
Phylogenetic reconstruction based on the concatenated sequences of *gyrB*, *rpoB*, and *rpoD* genes by the NJ algorithm, showing the position of *C. lacus* sp. nov. YLOS41^T^ and *C. lacus* XH07 among related taxa. Bootstrap values (expressed as percentages of 1000 replications) greater than 70% are shown at the nodes. *Flavobacterium haoranii* DSM 22807^T^ was used as an outgroup. Filled circles indicate that the corresponding nodes were also recovered in the ML algorithm. Bar, 0.050 substitutions per nucleotide position.

Nearly full-length 16S rRNA gene sequences (> 1400 bp) were obtained for strain YLOS41^T^ (MG641896) and XH07 (MK246815). On the basis of 16S rRNA gene sequence similarity, these two strains had 99.9% of 16S rRNA gene sequence similarity, and the type strains of *C. taklimakanense* (95.8% similarity), *Chryseobacterium salipaludis* (95.4%), *Chryseobacterium pallidum* (95.3%), and *Chryseobacterium hominis* (95.3%) were the closest neighbors to these novel strains. Phylogenetic analysis of the 16S rRNA gene sequences and MLSA analysis showed that the novel strains formed a tight group separated from the closest species within the genus *Chryseobacterium* (Figures 3, 4). The same topologies were obtained again after ML phylogenetic analysis. Comparing strains YLOS41^T^ and XH07, the identities of *gyrB*, *rpoB*, and *rpoD* were 86.32, 98.27, and 97.39%, respectively. In contrast, strain YLOS41^T^ shared low similarity values with other *Chryseobacterium* species tested (Supplementary Table S1). Phylogenetic analysis and 16S rRNA gene sequence similarity supported for the classification of the two strains as novel species within the genus *Chryseobacterium*, and strains YLOS41^T^ and XH07 may belong to one single species.

### Fingerprinting Analysis

The internal strain variation of the novel species was clarified using whole-cell MALDI-TOF MS and RAPD-PCR analysis. The two novel strains YLOS41^T^ and XH07 had different RAPD profiles based on primers P1 and P2, which demonstrated that these two strains are genetically different and are not clones of a single strain (Supplementary Figure S3). The obtained protein profiles for the two strains were species-specific with several strain-unique peaks that excluded the possibility of a clonal origin (Supplementary Figure S4).

### Chemotaxonomic Characteristics

The predominant fatty acids (relative amount > 10%) of the two strains were iso-C_15__:__0_ and anteiso-C_15__:__0_. The fatty acids of the two isolates were similar to related type strains, but with some differences in the relative amount of some fatty acids (Supplementary Table S2). The major polar lipids of the two isolates comprised phosphatidylethanolamine (PE), three aminolipids (AL1–AL3), and three unidentified lipids (L1–L3), which was in accordance with the two related type strains (Supplementary Figure S5).

### Genomic Analysis

**FIGURE 5:**
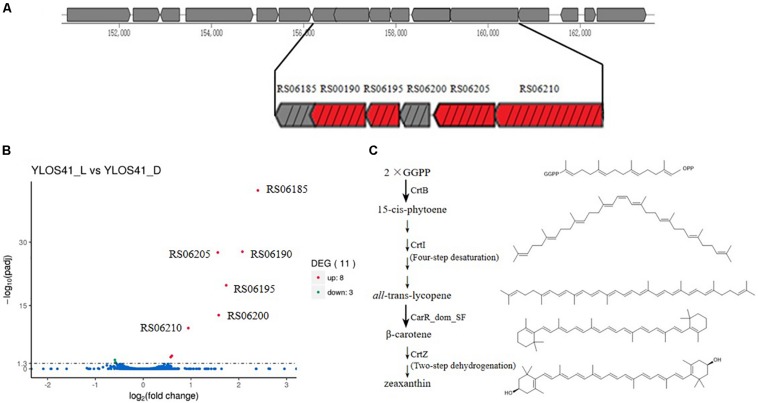
**(A)** Gene cluster of carotenoid synthesis by antiSMASH. RS06185, outer membrane lipoprotein; RS06190, lycopene cyclase CarR_dom_SF; RS06195, *crtZ*, beta-carotene hydroxylase CrtZ; RS06200, hypothetical protein; RS06205, *crtB*, phytoene synthase CrtB; RS06210, *crtI*, phytoene desaturase CrtI. Red box represents gene of synthetic enzyme of carotenoids. Box with black slash represents up-regulated gene in transcriptome. **(B)** Significantly different expressed genes of strain YLOS41^T^ under light vs. dark conditions. Statistical significance of genes with adjusted *P*-value (padj) < 0.05 regardless of the fold change was considered to be significant according to transcriptomic analysis. padj: significance difference index. **(C)** Proposed pathway of carotenoid biosynthesis in the strain YLOS41^T^. Chemical structure was drawn by Chemdraw.

The draft genome sizes of strains YLOS41^T^ and XH07 were 2.38 and 2.43 Mb, with G + C contents of 39.6 and 39.4 mol%, which contained 2,204 and 2,274 genes, respectively (Table 2). From the Venn diagram of annotation by KEGG, we found that strains YLOS41^T^ and XH07 shared 845 unique genes; strain YLOS41^T^ had 44 unique specific genes; strain XH07 had 54 unique specific genes (Supplementary Figure S6). According to the data obtained, the ANI and *is*DDH between strains YLOS41^T^ and XH07 were more than 96 and 70%, respectively. The ANI and *is*DDH values between the novel strains and other species tested varied from 70.47 to 86.76%, and 17.5 to 22.9% (Supplementary Table S3), which were clearly lower than the generally accepted cutoff thresholds of 95–96 and 70% for delineation of prokaryotic species (Richter and Rosselló-Móra, 2009; Meier-Kolthoff et al., 2013). The result confirmed that the two strains represent a novel species. The secondary metabolite gene cluster was predicted by antiSMASH, and only the carotenoid synthesis gene cluster was predicted in strains YLOS41^T^ and XH07. Four genes encoded carotenoid synthesis enzymes (Figure 5A). The flagellar assembly gene had only one, *motB*, but gliding motility-associated genes had 14, including *gldJ*, *gldN*, *gldM*, *gldL*, *gldK*, *gldH*, *gldD*, *gldB*, *gldC*, *gldF*, and *gldG*. The phenotypic characterization was consistent with the genome analysis.

**TABLE 2:** Genomic characteristics of *Chryseobacterium lacus* sp. nov. strains.

**Attribute**	**YLOS41^T^**	**XH07**
Accession no.	RWJH00000000	RWJG00000000
Genome size (bp)	2,375,938	2,428,930
DNA G + C content (mol%)	39.6	39.4
No. of contigs	32	38
Contig N50	349,994	302,431
Genes	2,204	2,274
Protein	2,139	2,175
rRNAs	5	8
tRNAs	41	39
Other RNA	3	3
Pseudogenes	16	49

### Transcriptomic Analysis

We analyzed the differentially expressed genes in the dark and with illumination to investigate the light-related genes in strain YLOS41^T^ (Figure 5B). Genes with an adjusted *P*-value of < 0.05 were assigned as significantly differentially expressed. The result showed that 11 (0.49%) of all identified genes were assigned as significantly differentially expressed regardless of the fold change. Under light conditions, most of the differentially expressed genes were up-regulated (8), and only three genes with unknown functions were down-regulated (Supplementary Table S4). Among the up-regulated transcripts, four coded for proteins involved in carotenoid synthesis, two were located in the *crt* cluster and its flanking region, but the relationship with carotenoid synthesis or regulation was still unknown, and the others have unknown functions in light response. As a result, it was confirmed that the difference in colony color under light and dark conditions was caused by the presence of carotenoids, and light irradiation significantly increased the carotenoid synthesis.

### Pathway of Carotenoids Synthesis

Through genome annotation, a total of four genes were shown to be directly involved in the synthesis of carotenoids, and they were significantly up-regulated in the transcriptome. Based on four genes, *crtB*, *crtI*, lycopene cyclase CarR_dom_SF gene, and *crtZ* (Figure 5A), we speculated on the synthetic pathway of carotenoid production of the strain YLOS41^T^ (Figure 5C). The first two steps of carotenoids synthesis were the same in all bacteria (Clotault et al., 2012). First, the condensation of two molecules of geranylgeranyl pyrophosphate (GGPP) formed 15-cis-phytoene by CrtB. Second, the four desaturation steps from 15-cis-phytoene to all-trans-lycopene were mediated by a single enzyme, CrtI (Linden et al., 1991). Lycopene was the starting compound for various group modifications that produce a large variety of carotenoids by different lycopene cyclase processes. We found the lycopene cyclase CarR_dom_SF gene that has been characterized as a novel fusion-type lycopene beta-cyclase gene (Tao et al., 2006). So, third, all-trans-lycopene was converted to β-carotene by lycopene cyclase CarR_dom_SF. Finally, β-carotene was further modified by CrtZ to produce zeaxanthin (Clotault et al., 2012). Therefore, based on the presence of the *crt* gene in the genome, we speculated that the synthetic carotenoid may be zeaxanthin. Then, the standard of zeaxanthin and carotenoids extracted from the strain YLOS41^T^ was analyzed by high-pressure liquid chromatography (HPLC), and the results supported the speculation (Supplementary Figure S7).

## Discussion

In this study, we identified a novel *Chryseobacterium* species based on the polyphasic approaches including phenotypic characterization, genomic comparison, sequence analysis of 16S rRNA and three housekeeping genes (*gyrB*, *rpoB*, and *rpoD*), fingerprinting analysis, and cellular fatty acid and polar lipid compositions. Strains YLOS41^T^ and XH07 were isolated from surface water. Microorganisms in the surface aquatic environment are exposed to visible light for a long time, which leads to an accumulation of ROS within the cells. In this study, we reported that *Chryseobacterium* species in the surface of water were resistant to light damage by synthesizing large amounts of carotenoids during the day, but not at night. To some extent, this differential expression reduces the metabolic cost. A well-known function of carotenoids is protection at the cellular level against harmful oxygen radicals, including photooxidative stress. The experimental results are consistent with the phenomena we observed and the function of carotenoids. Based on the presence of the *crt* gene in the genome, we speculated that the synthetic carotenoid was zeaxanthin.

Light irradiation induced carotenoid synthesis. This process required light-regulating systems to regulate carotenoid synthesis. However, no genes of the *car* operon or the *lit* operon were found in the genome of strain YLOS41^T^. Therefore, strain YLOS41^T^ may have a new light-inducted regulator. Among the significantly up-regulated genes of the *crt* cluster and its flanking region (Figure 5A), RS06190, RS06195, RS06205, and RS06210 encoded carotenoid synthetic enzymes, RS06185 encoded lipocalins family proteins (outer membrane lipoprotein), and RS06200 encoded hypothetical proteins. RS06185 was adjacent to the lycopene cyclase gene, and RS06200 was adjacent to the *crtB* gene. Therefore, these two genes should be largely related to the regulation of carotenoid synthesis or sensory perception of light signals. It was reported that outer membrane lipoprotein expression may be part of a more general stress response (David et al., 2003; Campanacci et al., 2004). Among the previously reported bacteria with light-induced carotenoid synthesis and light-induced transcriptional regulators, CarA/LitR were adjacent to the *crtB* gene (Takano et al., 2011, 2014). So, we boldly speculated that RS06185 could respond to the stress caused by light stimulation and that RS06200 may be involved in light-induced regulation like CarA/LitR.

In this strain, except for the genes involved in carotenoid synthesis, there are many significantly up-regulated and down-regulated genes and their relationship with light induction is still unknown. In addition, the light-regulated mechanism in this strain has an unknown pattern. This mechanism is worth exploring, which will provide new insight into the physiology and ecology of bacteria.

### Description of *Chryseobacterium lacus* sp. nov.

*Chryseobacterium lacus* [*la*’*cus*. L. gen. n. *lacus* of a lake or pond, referring to the source of the type strain].

Cells are Gram-stain-negative, rod-shaped (0.5–0.7 μm wide and 0.8–2.6 μm long), facultative anaerobic, and gliding. Colonies are circular, smooth, and yellow-colored under light conditions and white-colored under dark conditions on modified agar 2216. Growth occurs at 4–40°C (optimum, 33°C), pH 6.5–9.0 (optimum, pH 7.0–8.0), and 0.0–2.0% (w/v) NaCl (optimum, 0.0%). Cells are oxidase-negative and catalase-positive. The isolates do not produce flexirubin-type pigments. Carotenoids are detected under light cultivation and not under dark cultivation. Gelatin, casein, and Tweens 20 and 60 are hydrolyzed but starch and cellulose are not. Nitrate reduction and citrate utilization are negative. Production of indole is positive. Tests are positive for gelatinase, alkaline phosphatase, esterase (C4), esterase lipase (C8), leucine arylamidase, cystine arylamidase, acid phosphatase, naphthol-AS-BI-phosphohydrolase, and valine arylamidase; tests are negative for o-nitrophenyl-β-D-galactopyranoside, arginine dihydrolase, lysine decarboxylase, ornithine decarboxylase, urease, tryptophan deaminase, lipase (C14), trypsin, α-chymotrypsin, α-galactosidase, β-galactosidase, β-glucuronidase, α-glucosidase, β-glucosidase, *N*-acetyl-β-glucosaminidase, α-mannosidase, and α-fucosidase. In Biolog GEN III microplates, D-raffinose, α-D-glucose, gelatin, glycyl-L-proline, L-arginine, L-histidine, glucuronamide, Tween 40, acetoacetic acid, and acetic acid are oxidized. Acid is produced from D-Ribose, D-glucose, aesculin ferric citrate, D-tagatose, and potassium 5-ketogluconate in the API 50 CHB fermentation kits. The major fatty acids are iso-C_15__:__0_ and anteiso-C_15__:__0_. The major polar lipids are phosphatidylethanolamine, three aminolipids, and three unidentified lipids. The DNA G + C of the type strain is 39.6 mol%.

The type strain, YLOS41^T^ (= KCTC 62352^T^ = MCCC 1H00300^T^), was isolated from freshwater collected in Yilong Lake, Yunnan Province, China. A second strain, XH07 (= KCTC 62993) was isolated from freshwater collected in West Lake of Dali, Yunnan Province, China. The GenBank accession numbers for the 16S rRNA gene sequence and genomic accession number of *C. lacus* sp. nov. YLOS41^T^ are MG641896 and RWJH00000000, respectively. The GenBank accession numbers for the 16S rRNA gene sequence and genomic accession number of *C. lacus* XH07 are MK246815 and RWJG00000000, respectively. The SRA accession numbers are SRR10343458, SRR10343457, SRR10343456, SRR10343455, SRR10343454, and SRR10343453.

## Data Availability Statement

The datasets generated for this study can be found in the MG641896, RWJH00000000, MK246815, RWJG00000000, SRR10343453, SRR10343454, SRR10343455, SRR10343456, SRR10343457, and SRR10343458.

## Author Contributions

JZ and Z-JD designed the study. JZ performed most of the laboratory work and data analysis, analyzed the experimental results, and wrote the manuscript. CG and X-MY carried out the experiments. H-YL performed the fatty acid analysis. Z-JD, CG, and X-MY revised the manuscript.

## Conflict of Interest

The authors declare that the research was conducted in the absence of any commercial or financial relationships that could be construed as a potential conflict of interest.

## Footnotes

1 www.bacterio.net/chryseobacterium.html
