# Supplementary material for: Chryseobacterium lacus sp. nov. Isolated From the Surface Water of Two Lakes With Light-Induced Carotenoid Production
Source: Front Microbiol. 2020 Mar 4;11:251. doi: 10.3389/fmicb.2020.00251 (PMC7064467; doi:10.3389/fmicb.2020.00251)
Supplement: Supplementary file 1 [file Data_Sheet_1.docx]

Supplementary Material

Sequences of housekeeping genes

>Chryseobacterium_bovis_DSM 19482T

AGTATTCAGGCATTGGAAGGGATGGAGCACGTGCGTATGCGTCCGTCAATGTACATTGGTGATGTAGGCGTAAGAGGTCTCCACCATTTGGTTTATGAAGTCGTGGATAACTCCATTGATGAGGCTTTGGCAGGATATTGCGATACAATTACCGTTTCTATCAAAGAAGGTAATGGTGTGGAAGTGACCGATAACGGCCGTGGTATCCCGGTAGATTTTCACGAGAAAGAACAGAAGTCGGCGCTTGAGGTTGTAATGACCAAAATTGGTGCGGGTGGTAAATTTGATAAAGATTCTTACAAAGTTTCCGGAGGTCTTCACGGCGTTGGGGTTTCTTGCGTTAACGCACTTTCCAACGAGATGGTTACAACGGTCTATAGAGACGGAAATGTTTATCAGCAGGTCTATTCCCGAGGAAAAGCTCAGACAGGCGTTGAAGAAATAGGTCATAGTGATAAAA-GAGGAACCAAGCAATTCTTCCAGCCGGATGATACTATTTTCACAGAG---CTGGTTTATAACTATGACACATTGGCAAGCCGCCTGAGAGAGCTGTCTTACCTTAACAAAGGTATTACGATTACTTTGACTGACGAGAGAGAGAAACTGGAAGACGGGACTTACCGTACAGAAGTTTTCCATTCAGAAGGTGGCCTGAAAGAATTTGTAGAATATATTGATGGCAACAGAGAAGCTATCATGGAGCATGTTATCTTTATGGAAGGCGAACGCGATAATATCCCGGTAGAAGTGGCGATGCGTTACAACACGTCTTTCAACGAGAATCTGCATTCTTATGTTAATAATATCAATACGCACGAGGGTGGTACCCACCTGGCAGGTTTCAGAAGAGCTTTGACTAGAACTTTGAAAAAATATGCCGATGAATTGGGATTGCCTGCAAAGGAAAAAGTAGAAGTGACAGGTGATGATTTCCGTGAGGGATTGACTGCCGTGATTTCTGTAAAAGTAATGGAGCCCCAGTTTGAAGGGCAAACCAAAACCAAATTGGGTAACTCAGAGGTTTCCGGTGCTGTAGATAAAATTGTGGGCGAAATGCTGTCTAACTTCCTGGAAGAAAATCCACTGGAAGCTAAGCAAATCGTGCAAAAAGTGGTTTTGGCAGCCAAAGCAAGACAGGCCGCGAAGAAAGCGCGAGAAATGGTGCAGCGTAAATCACCAATGGGTGGATCTGGCTTGCCAGGAAAATTATCCGATTGTTCTTCAAAAGATCCGGCTGAATCAGAGCTGTTTTTGGTAGAGGGAGATTCCGCAGGTGGAACGGCAAAACAAGGCCGTGACCGTCATTTTCAGGCAATTTTGCCACTGAGAGGTAAAATATTGAATGTTGAGAAATCAATGCTTCATAAAGTCTACGACAACGAGGAAATTAAAAATATTTATACCGCACTTGGCGTTTCTGTAGGGACAGAAGAAGATTCCAAAGCCCTGAATATGGCAAAGCTACGTTATCACAAGATCGTGATTATGACCGATGCCGATATCGATGGTTCCCACATTTCTACTTTGATTTTGACATTCTTCTTCCGTTATATGAAGGAGATGATTGAGAATGGATACATTTATATTGCGCAACCGCCTTTGTATCTTTTGAAAAAAGGTAACAAAAAAATGTATGCTTATAATGAGAAAGAACGTGAACAATTTACATTGGAGATGTCACCGGACGGCAAAGGTGTAGAAGTGCAGCGTTACAAAGGTCTTGGAGAGATGAATCCTGAGCAGCTTTGGGAAACCACGCTTAATCCCGAACACAGAATATTGAAACAGGTAACGATCAACAATGCCGTGGAAGCTGACTCAACCTTCTCTATGCTGATGGGCGATGAAGTACCGCCAAGACGTGAGTTTATCGAGAAAAATGCAGTCTATGCAAGAATCGATGAGTTCTCTATCATCCAGAATACATTACAAAAAGACCCTACCAACTCAGAAAAAGAAGCGGTAGAGTACATCTATCGTCAGTTAAGAAATGCTGACGCACCGGATGAGGAAACTGCAAGAGGGATTATAGAAAAATTATTCTTCTCTGAGCAGCGTTATTCATTAGGCGAAGTTGGTCGTTACAGATTAAATAAAAAATTAGGTCTTAACATTCCGGAAACGACGGAAGTACTGACCAAGGAAGATATCATCGCAATTGTAAAACACCTGATCGAGTTGGTTAATTCCAAAGCGGAGGTAGATGATATTGACCACCTTTCTAACAGACGTATCAAAACTGTGGGCGAGCAATTGTCTGGTCAGTTTGGTGTCGGTCTTTCCAGGATTGCGAGAACTATCAAGGAAAGAATGAACGTAAGAGATAACGAGATCTTTACACCGGTAGACTTGGTTAATGCCAAGACTTTGACGTCTGTTATTAACTCGTTCTTCGGAACTAACCAGCTTTCTCAGTTTATGGATCAGACCAATCCACTGTCAGAGATCACGCACAAGCGTAGATTATCTGCACTAGGGCCTGGTGGTTTATCAAGAGAAAGAGCAGGTTTTGAGGTTCGTGACGTTCACCATACCCACTACGGTAGAATTTGTCCAATCGAAACTCCGGAAGGACCAAACATTGGTTTGATCTCTTCTTTGGGTATGTATGCAAAAATCAACAATCTTGGTTTCATCGAGACGCCTTACAGAAAAGTTGAAGACGGTAAAGTAGATCTTAAAGCAGCTCCAATTTATCTGAATGCAGAAGATGAAGAAGATAAAGTAATTGCTCAGGCTAACGTTGCATTGAGTGATGATGGTAACTTTGAGACAGACAGGATTATCGCACGTCTTGACGGTGATTATCCGGTTGTTGAGCCTCAACAGGTAGATTTGATAGATGTGGCACCAAACCAGATTTCTGGTATTTCTGCATCATTGATTCCGTTCCTGGAGCATGATGATGCGAACCGTGCCTTGATGGGATCTAACATGATGCGTCAGGCCGTTCCGTTGTTGAAGCCTCAGGCACCAATTGTTGGTACAGGTTTAGAAAAACAGGTAGCAAGAGACTCCCGAGTATTGATTAATGCAGAGGGTAATGGTGTTGTAGAATATGTAGATGCTGAGAGAATCACTATTAAGTACGAGAGAAGCGAAGAAGACGATTTAGTTAACTTTGATTCTGCGACCAAAACTTATAAACTGACTAAATTCAGAAAAACCAACCAAAGTACAACGATTACTTTGAGACCAAACGTAAGAGTTGGTGATAAAATCGAGAAAGGACAGGTTCTTTGTGACGGTTATGCAACCGAAAACGGAGAATTGGCATTGGGTAGAAACCTTGTGGTAGCCTTCATGCCTTGGAAAGGTTATAACTTTGAGGATGCGATCGTAATCAACGAGAAAGTAGTAAGAAACGACTGGTTTACTTCTATCCACGTAGATGAATACTCATTGGAAGTTCGTGATACCAAATTAGGTATGGAGGAATTGACAGCTGATATCCCTAACGTGTCCGAAGAAGCGACCAAAGATCTTGATGAGAATGGTATGATCCGTATTGGAGCAGAAGTAAAACCTGGTGATATCCTTATCGGGAAAATCACACCAAAAGGTGAATCTGACCCAACACCGGAAGAGAAATTACTTCGTGCGATTTTTGGTGACAAAGCCGGAGATGTAAAAGATGCTTCTCTTAAAGCTGATTCTTCATTAAGAGGTGTTGTAATCAACAAAAAGTTGTTCTCAAGAAACATCAAGGACAAAAAGAAAAGAAGTGAAGAAAAAATCAAACTGGAAGAAATCGAAAATACTTACAAAGCTAAGTTTGACGATTTGAGAAACACTTTGATTGAGAAACTTAATACTTTGGTTTCCGGTAAAACTTCTCAGGGCGTGAAAAACGATCTGGAAGAAGAAGTTATTGGTAAAGGTACCAAGTTTACGCTGCGACTTCTTCAGTCTGTAGAAGATTATAACAACATCAGTGGTGCCGACTGGACTGTGGATGCTGACAAAAATGATCTGATCAAGCAATTGATCCATAATTATAAAATCAAATACAACGATCTTTCCGGTGTCAAAAACCGTGAGAAATTTGCATTGTCTATCGGTGATGAGCTACCTGCAGGTATCATCAAACTGGCTAAGGTTTATATTGCTAAAAAACGTAAACTGAATGTTGGGGATAAAATGGCGGGTCGTCACGGTAACAAAGGTATCGTTTCAAGAATCGTTCGTGAAGAAGATATGCCATTCCTAGAAGACGGGACACCGGTAGATATCGTTCTTAACCCACTTGGGGTACCTTCCCGTATGAACATTGGTCAGATCTACGAGACCGTTCTTGGTTGGGCTGGTAAGCAGTTGGGACTTAAGTTCGCTACACCAATCTTTGATGGCGCAAGTCTTGAGCAAATCACCGAGTATACAGATCAGGCAGCACTTCCTAAATTTGGAAATACGCACCTTTATGATGGTGGTACTGGTGAGAGATTTACACAGCCGGCTACGGTAGGTGTGATCTATATGCTGAAACTGGGACACATGGTAGATGATAAGATGCACGCACGTTCTATCGGACCTTACTCATTGATTACGCAACAGCCGTTAGGAGGTAAAGCGCAATTTGGAGGACAGCGTTTCGGAGAGATGGAAGTTTGGGCTCTAGAAGCTTTTGGAGCATCCAATATCTTGAGAGAAATCCTGACTGTGAAGTCGGATGACGTGATAGGTAGAGCGAAAACTTATGAAGCAATTGCAAAAGGAGAAGCAATGCCAGAACCTGGTATTCCGGAATCCTTCAACGTATTACTTCACGAGTTACAAGGTCTTGGACTTGATGTAAGAATCTTACTTCTGGAGTTATGTTTCAGTCTTTTGATTGCTTTTTCTTTTATCTGGCGCACTCTTTCTCTTGTAAGGTCAAAAGTTTCGCCGATCTCTTCCAGCGTCATTGGATGTTTACCGTTAAGGCCAAAATACAATCTCACAAGATCCGCCTCTCTTGGCGTCAGCGTGTTAAGTGCTCTTTCGATCTCAATTTGCAGCGATTCCAGCATCAGGTCTTTATCCGGGCTTGGTGATTCTCCGGAACGTAAAACATCATAAAGGTTAGAATCTTCACCTTCCACAAGCGGCGCATCCATAGACAGGTGTCGGCCACTGTTTTTCATAGATTCCTTGATGTCTTCCTCGCTCATGTCAAGCACCTCTGCCAGTTCTTCGGGAGACGGTGGTCTCTCATTTTCCTGCTCCAGATGGGCATAAGCCTTGTTGATCTTGTTGATAGAACCAATTTTATTAAGAGGTAACCTTACAATACGGGATTGCTCTGCCAAAGCCTGAAGAATAGACTGGCGGATCCACCATACCGCATAGGAGATAAATTTGAAACCTCTGGTCTCATCATATCTTTTGGCGGCCTTCATCAATCCAAGATTCCCTTCATTAATCAAATCCGGAAGAGAAAGTCCCTGATTCTGGTACTGTTTGGATACAGAAACCACGAAACGAAGATTCGCTTTGATCAGTTTTTCCAGCGCAGCCCTGTCTCCGGCACGTATTCTTTGTGCCAAATCTACCTCTTCATCAGCGGTAATCAGTTCTACTTTCCCGATCTCTTGCAGATACTTGTCGAGTGATGCGGTTTCCCTGTTGGTAACCTGTTTTGTAATTTTTAATTGTCTCAT

>Chryseobacterium_caeni_DSM 17710T

AGTATTCAGGCATTGGAAGGAATGGAGCACGTTCGTATGCGTCCTTCGATGTACATTGGTGATGTAGGGGTCAGAGGTCTCCACCATTTGGTTTATGAAGTAGTGGATAACTCTATCGATGAGGCTTTGGCAGGATATTGTGATACGATCACTGTCGCGATAAAGGAAGGTAATGCCGTAGAAGTTATGGATAACGGTCGTGGTATTCCTGTAGATTTTCACGAGAAGGAACAGAAATCTGCGCTGGAGGTTGTAATGACCAAAATCGGAGCTGGAGGTAAGTTCGATAAAGATTCTTACAAAGTTTCCGGAGGTCTTCACGGCGTTGGGGTTTCCTGTGTTAACGCACTTTCCAACGAGATGATTACTACCGTTTACAGAGATGGGAATATCTATCAGCAGGTTTATTCCAGAGGGAAGGCACAAACAGGTGTTGAAGAGATCGGACACAGTGACAAAA-GAGGAACAAAACAGTTTTTCCAACCGGATGATTCCATTTTTACTGAA---TTGGTTTACAACTACGATACTTTGGCAAATCGGCTTAGAGAGTTGGCATACCTTAATAAAGGAATTACGATTACTTTAACAGACGAAAGAGAAAAACTGGAAGACGGAACTTTCAGAACGGAAGTTTTCCATTCGGAAGGTGGTTTGAAAGAATTCGTTGAGTATATCGACGGAAACCGTGAGTCTATTATGGAGAATGTGATCTTTATGGAAGGCGAGCGTGACGATATTCCGGTTGAGGTGGCGATGCGTTATAACACGTCTTTCAACGAGAATCTCCACTCTTATGTTAATAATATTAATACTCACGAAGGAGGAACACACTTAGCAGGTTTCAGAAGGGCTTTAACAAGAACGCTTAAGAAATATGCCGATGAACTAGGACTTCCTGCAAAGGAAAAGGTTGAAGTTACTGGAGACGATTTCCGTGAAGGTTTAACAGCTGTGATTTCTGTAAAAGTAATGGAACCTCAGTTCGAAGGTCAGACCAAAACGAAATTAGGCAACTCAGAAGTTTCTGGTGCGGTTGATAAAATCGTGGGCGAAATGCTGACGAACTTCCTCGAGGAAAATCCTGCTGAAGCCAAGATCATTGTTCAGAAAGTTGTTTTGGCAGCGAAGGCAAGGCAAGCAGCGAAGAAGGCAAGAGAAATGGTTCAGAGAAAATCTCCGATGGGAGGTTCTGGTCTGCCAGGAAAATTGTCGGATTGTTCTTCAAAAGACCCGGCTGAATCAGAATTGTTCCTTGTAGAGGGAGATTCCGCAGGTGGAACGGCTAAACAAGGTCGTGACCGTCATTTTCAGGCAATTTTGCCTTTGAGAGGTAAGATCCTGAATGTTGAGAAATCTATGGTCCATAAAGTTTATGATAACGAGGAGATCAAGAATATCTATACGGCGCTTGGCGTTTCTGTAGGAACAGAAGAGGATTCCAAAGCGTTGAATATGGCAAAACTTCGTTACCACAAAATTGTGATTATGACCGATGCCGATATTGATGGTTCTCACATCTCAACTTTGATTCTGACTTTCTTCTTCAGATATATGAAGGAATTGATTGAGAACGGATATATTTATATCGCTCAGCCGCCTTTATATCTATTGAAAAAAGGAAACAAAAAAACTTATGCATACAACGAGAAGGAGCGTGAACAATTCACTCTTGAAATGTCTCCGGACGGAAAGGGAGTAGAGGTACAACGTTACAAAGGTCTTGGAGAGATGAATCCTGAGCAGCTTTGGGAAACAACGCTGAATCCGGATGGCAGGATCCTGAAACAAGTAACCATTGACAACGCGGTAGAGGCAGATTCTACATTCTCTATGCTGATGGGTGATGAAGTTCCGCCGAGAAGAGAATTCATTGAGAAAAATGCGGTTTACGCAAGAATTGATGAGTTCTCTATTATCCAGAATACTTTACAAAAAGATCCAACCAACTCAGAAAAAGAGGCGGTGGAATATATTTATCGTCAGCTAAGAAATGCTGATGCTCCGGATGAGGAAACTGCAAGAGGAATTATTGAGAAATTATTCTTCTCAGAGCAGAGATATTCATTAGGTGAAGTTGGTCGTTACAGATTGAATAAAAAACTAGGTCTTAACATTTCTGAAGAAAATCAAGTTCTTACAAAAGAGGATATCATCTCTATCGTAAAACACTTGATCGAATTGGTTAACTCTAAGGCTGAGGTTGATGATATCGATCACTTGTCAAATAGAAGAATAAAAACTGTTGGAGAGCAATTGTCAGGACAATTTGGAGTAGGTCTTTCTAGAATTGCCAGAACAATCAAAGAAAGAATGAACGTTAGAGATAACGAGATCTTTACACCGGTTGACTTGGTTAATGCTAAGACTTTGACATCTGTTATCAACTCATTCTTCGGTACCAACCAGCTTTCCCAGTTCATGGACCAGACCAATCCATTGTCGGAGATCACTCACAAGAGAAGATTATCTGCACTAGGACCTGGTGGTCTATCCAGAGAAAGAGCAGGTTTCGAGGTTCGTGACGTTCACCATACACACTATGGTCGTATTTGTCCGATCGAGACTCCGGAAGGACCAAACATTGGTTTGATCTCTTCTTTGGGTATGTATGCGAAAATTAATACTTTAGGTTTCATCGAGACTCCTTATAGAAAAGTAGAAAACGGAAAAGTAGATCTTAATGCTGCGCCTGTTTATCTAAATGCTGAGGACGAAGAAAATCAAGTTATTGCTCAGGCAAACGTTGCCTTGAGTGATGATGGTAATTTCGAAACTGATAGAATTATTGCCCGTTTGGATGGTGATTATCCTGTGGTTGAGCCTCAACAAGTAGACCTTATTGATGTTGCACCAAACCAGATCTCTGGTATTTCCGCTTCATTGATCCCATTCCTGGAGCATGATGATGCGAACCGTGCATTGATGGGATCCAACATGATGCGTCAGGCTGTTCCATTATTGAAGCCTCAGGCACCGGTTGTAGGTACAGGTTTGGAAAAACAAGTAGCAAGAGACTCTAGAATTTTGATCAATGCAGAAGGAACTGGAGTTGTAGAATATGTGGATGCTGAGAAAATTGTTATTAAGTACGAAAGAAGCGAAGAAGACGATCTTGTTAACTTCGATTCTGCTACTAAAACTTATAAACTGACCAAGTTCAGAAAAACCAACCAGAGTACGACTATTACATTGAGACCAAACGTAAGAGTAGGTGATACAGTGGAAAAAGGTCAGGTTCTTTGTGATGGTTATGCAACTGAGAACGGGGAATTAGCTCTTGGTAGAAACTTGGTAGTGGCGTTCATGCCTTGGAAAGGTTATAACTTCGAGGATGCGATCGTGATCAATGAAAAAGTAGTTCGTGAGGACTGGTTTACTTCGATCCACGTTGATGAATATTCTTTGGAAGTTCGTGATACCAAATTAGGTATGGAAGAATTGACAGCAGATATTCCTAACGTTTCTGAAGAAGCCACCAAAGATCTGGATGAGAATGGTATGATCCGTATCGGAGCAGAAGTGAAGCCTGGTGATATCCTTATCGGTAAGATCACGCCTAAAGGTGAGTCTGATCCAACACCGGAAGAAAAACTTCTTAGAGCGATCTTCGGGGATAAAGCTGGTGATGTAAAAGATGCTTCATTGAAAGCAGATTCTTCTCTTAGAGGAGTTGTTATCAACAAAAAATTGTTCTCTAGAAATATCAAGGACAAAAAGAAAAGAAGCGAAGAGAAGATCAAACTTGAGGAAATCGAAAATACTTACAAAAATAAGTTCGACGACCTAAGAAACACTTTGATTGAAAAATTGAATACCTTGGTTTCTGGTAAAACTTCTCAGGGTGTGAAAAATGACCTTGAAGAGGAAGTAATCAGCAAAGGAACCAAATTCACTTTGAAATTGCTTCAGTCTGTAGAAGATTATGTGAACATCAGCGGTGCAGATTGGACCGTAGATGCAGACAAAAACGAATGGATCAAACAATTGATCCACAACTATAAGATCAAATACAATGATCTTTCGGGAGTTAAAAACCGTGAGAAATTTGCATTGTCTATCGGAGACGAGTTACCAGCAGGTATTATCAAATTAGCTAAAGTTTACATCGCTAAGAAACGTAAACTGAATGTAGGTGATAAAATGGCAGGTCGTCACGGTAACAAAGGTATCGTTTCAAGAATTGTTCGTGAAGAAGATATGCCATTCTTGGAGGATGGAACACCAGTAGATATCGTATTGAATCCACTTGGGGTACCTTCCCGTATGAACATTGGTCAGATCTACGAAACTGTTCTTGGATGGGCAGGTACAAAACTGGGATTGAAGTTTGCAACACCGATCTTTGATGGTGCAAGTCTTGAGCAGATCACAGAATATACTGATCAGGCAGGTCTTCCGAAATTTGGTAGTACACACTTGTATGACGGTGGAACTGGTGAGAGATTTACGCAGCCCGCAACTGTCGGTGTGATCTATATGTTGAAATTGGGACACATGGTAGATGATAAGATGCACGCACGTTCTATCGGACCTTACTCATTGATCACGCAACAGCCTTTAGGAGGTAAAGCGCAATTCGGAGGTCAGAGATTTGGAGAGATGGAGGTTTGGGCTCTTGAAGCATTTGGAGCGTCTAACATCCTGAGAGAGATCTTAACTGTGAAGTCGGATGACGTGATTGGTAGAGCAAAAACTTATGAAGCGATTGCTAAAGGTGAAGCAATGCCAGAACCTGGTATTCCGGAATCTTTCAACGTATTACTTCACGAGTTACAGGGTCTTGGACTTGATGTAAGAATCTTACTTCTTGTATTATGTTTCAATCTCTTGATCGCCTTTTCTTTGATCTGACGAACCCTTTCTCTTGTAAGGTCAAAAGTTTCACCGATTTCTTCCAAAGTCATTGGATGTTTTCCGTTCAGCCCGAAATATAATCTCACCAAATCGGCCTCTCTCGGAGTCAAAGTTTGTAGTGCTCTTTCTATCTCGATCTGTAGAGATTCCAGCATTAGATCCTTATCGGGACTTGGCGATTCTCCGGAACGCAAAACATCATATAAGTTAGAATCTTCACCTTCCACAAGTGGCGCATCCATCGATAGGTGACGACCACTGTTTTTCATAGATTCCTTGATGTCCTCCTCGCTCATATCCAGAACTTCTGCCAGCTCTTCCGGAGAAGGCGGTCTTTCGTTCTCTTGCTCCAGGTGAGCATAAGCCTTGTTGATCTTGTTGATAGAACCAATCTTATTCAAAGGTAATCTTACGATTCTCGACTGTTCGGCCAGCGCTTGCAAAATCGATTGACGAATCCACCAAACAGCATAAGAGATGAATTTGAAACCTCTCGTTTCATCATATCTTTTTGCCGCTTTCATCAGACCAAGATTTCCTTCGTTGATGAGATCCGGAAGAGAAAGACCTTGATTCTGATACTGTTTAGAAACCGAAACCACGAAACGTAAGTTAGCTTTGATCAGTTTTTCAAGAGCAGCTCTGTCACCGGCACGGATCCTCTGAGCCAAATCTACCTCCTCGTCAGCGGTGATCAATTCAACTTTTCCGATCTCTTGCAGATACTTGTCTAGTGATGCGGTTTCCCTGTTGGTAACCTGTTTTGTAATCTTTAATTGTCTCAT

>Chryseobacterium_gleum_JCM 2410T

AGTATTCAGGCATTGGAAGGAATGGAACACGTTCGTATGCGTCCTTCAATGTACATTGGTGATGTAGGAGTCAGAGGACTTCACCACTTGGTTTATGAAGTAGTAGATAACTCTATTGACGAGGCATTGGCAGGGTACTGCGATACGATCTTCGTTAGCATCAAGGAAGGAAACGGAATTGAAGTAAGTGATAACGGTAGAGGTATCCCGGTTGATTTCCACGAAAAGGAGCAGAAATCAGCCCTTGAAGTTGTAATGACTAAAATCGGGGCCGGAGGTAAGTTTGATAAAGACTCTTATAAGGTTTCAGGAGGTCTTCACGGGGTTGGGGTTTCGTGTGTGAATGCACTTTCCAATGAGATGATCACTACTGTTTACAGAGACGGAAACGTTTATCAGCAGATTTATTCCAGAGGAAAAGCGCAGACCGGTGTTGAAGAGATTGGTCACAGCGAAAGAA-GAGGAACCAAGCAGTTTTTCCAGCCGGATGATACTATATTTACAGAA---TTAGTTTACAATTACGATACATTAGCAAGCCGTTTAAGAGAGCTTTCTTACCTTAATAAAGGAATTACCATTACGCTTACCGATGAAAGAGAAAAATTGGAAGACGGATCTTTCCGTTCAGAAGTTTTCCATTCTGAAGGTGGTTTGAAGGAGTTTGTTGCTTATATTGACGGTAACCGTGAATCTATCATGGAGCATGTGATCTTCATGGAAGGAGAAAGAGATGATATCCCGGTAGAAGTAGCAATGCGTTACAATACTTCTTTCAATGAGAATCTTCACTCTTACGTTAACAATATCAACACCCATGAAGGGGGAACTCACCTGGCAGGTTTCAGACGTGCTTTGACGAGAACCCTTAAGAAATATGCTGATGATTTAGGAATTCCGCAGAAGGAAAAAGTAGAAGTTACAGGAGATGACTTCCGTGAAGGTTTAACAGCTGTAGTTTCCGTAAAAGTAATGGAGCCTCAGTTTGAAGGGCAAACTAAAACAAAATTAGGAAACTCTGAAGTTTCAGGGGCTGTAGATAAAATTGTAGGGGAAATGCTTACCAATTTCCTTGAAGAAAACCCAAATGAAGCGAAGCAGATCGTTCAGAAAGTGGTTTTGGCAGCAAAAGCAAGACAGGCCGCTAAGAAAGCGCGTGAAATGGTTCAGAGAAAATCTCCGATGGGAGGTTCCGGTCTTCCTGGAAAACTATCTGACTGTTCATCAAAAGATCCGGCAGAATCTGAAATCTTCCTTGTAGAGGGAGATTCCGCAGGGGGTACAGCCAAGCAGGGAAGAGACAGACACTTCCAGGCTATTCTTCCGTTAAGAGGTAAAATCCTGAACGTAGAAAAATCTATGCTTCACAAAGTATATGATAACGAAGAGATCAGAAATATCTATACAGCTCTTGGAGTTTCCGTAGGAACTGAGGAAGACAGCAAAGCATTGAACTTATCAAAACTGAGATACCACAAGATCGTTATCATGACCGATGCCGATATTGACGGATCTCACATTTCCACACTGATTCTTACTTTCTTCTTCAGATATATGAAAGAACTTATTGAGAATGGTTATATCTATATCGCTCAACCACCTTTATACCTGTTAAAGAAAGGAAACAAAAAAGTATACGCTTACAACGAAAAAGAGCGTGAAGAGTTTACTCTGGAAATGTCTCCGGACGGAAAAGGGGTAGAGGTACAGCGTTACAAAGGTCTTGGAGAAATGAACCCTGAGCAGCTTTGGGAAACAACCCTTAACCCTGAACACAGAATTCTGAAGCAGGTAACCATTGATAATGCAGTGGAAGCTGACAGTATTTTCTCCATGTTGATGGGTGACGAGGTTCCGCCAAGAAGAGAATTTATTGAGAAAAATGCAAAATATGCCAAAATTGATGAATTCTCTATCATCCAGAATACATTACAAAAAGACCCTACCAACTCTGAAAAAGAAGCGGTAGAGTACATTTATCGTCAGTTAAGAAACGCAGATCCGCCAGATGAGGAAACTGCAAGAGGAATCATTGAAAAATTATTCTTCTCTGAGCAGAGATATTCTTTAGGTGAAGTGGGACGTTACAGATTGAACAAAAAATTAGGTCTTAACATTCCTACTACAACTGAGGTTCTTACAAAAGAAGATATTATTGCTATCGTAAGACACCTTATCGAACTTGTTAACTCTAAAGCTGAGGTTGATGATATTGACCACTTATCAAACAGAAGAATTAAAACTGTTGGTGAGCAATTAGCAGGACAGTTTGGTGTAGGTCTTTCAAGAATTGCAAGAACAATCAAGGAAAGAATGAACGTTAGAGATAACGAAATCTTTACTCCGCTTGACCTTGTAAATGCAAAGACATTAACATCTGTAATCAACTCATTCTTTGGTACCAACCAGCTATCTCAGTTCATGGACCAAACCAACCCTCTGTCAGAGATCACTCACAAGAGAAGATTATCTGCACTGGGACCTGGTGGTTTATCAAGAGAAAGAGCAGGTTTTGAGGTTCGTGACGTTCACCATACTCACTATGGAAGAATTTGTCCGATTGAAACTCCGGAAGGACCAAACATCGGTTTGATTTCATCTTTAGGAATTTATGCGAAAATCAACAACCTAGGTTTCATCGAAACTCCATATAGAAAAGTGGAAAGTGGTAAGGTTGATCTTAACGCTGATCCTATTTACTTAAATGCAGAAGACGAAGAAGCTAAGGTAATTGCTCAGGCAAACGTTGAATTGAGCGATAACGGTGATTTCGAAACAGACAGAATTATTGCAAGATTGGATGGTGATTATCCGGTAGTAGAACCTAATCAGGTAGACCTTATCGACGTAGCACCAAACCAGATTTCCGGTATTTCCGCTTCATTGATTCCATTCCTGGAGCATGATGATGCGAACCGTGCATTGATGGGATCTAACATGATGCGTCAGGCCGTTCCTCTATTGAAGCCACAGGCTCCGATTGTTGGTACAGGGCTTGAGCAACAAGTTGCAAGAGATTCAAGAATTTTAATTAACGCTGAAGGTACAGGTACAGTACAGTACGTAGATGCTGACAAAATCGTAATTAAGTACGAAAGAAGCGAAGACGAAGATTTAGTACAATTCGAGTCTGCTACTAAAACATACAACCTTACCAAGTTCAGAAAAACCAACCAGAGTACAACCATTACCCTAAGACCAAACGTAAGGGTAGGTGATGTAGTGGAAAAAGGACAAGTACTTTGTGACGGTTATGCTACTGAAAAAGGAGAATTGGCTCTTGGTAGAAACTTAGTGGTAGCGTTCATGCCTTGGAAAGGATACAACTTCGAGGATGCGATCGTAATCAACGAAAAAGTTGTACGTGAAGACTGGTTTACTTCAATCCACGTAGATGAATATTCTCTTGAAGTTCGTGATACCAAATTAGGTATGGAAGAGCTTACAGCAGATATTCCAAACGTATCTGAAGAAGCTACCAAAGATCTTGACGAGAACGGTATGATTAGAATCGGTGCTGAAGTGAAGCCTGGAGATATTATGATTGGTAAAATCACTCCAAAAGGTGAATCAGACCCGACTCCTGAAGAAAAACTTCTTAGAGCAATCTTCGGTGATAAAGCTGGTGATGTGAAGGATGCTTCATTGAAAGCTGACTCTTCATTAAGAGGAGTTGTTATCAACAAGAAATTGTTCTCCAGAAATATTAAAGACAAAAAGAAAAGAACTGAAGAAAAACTTAAACTTGAAGAGATTGAAAACACTTACAAGGCTAAGTTTGACGAGTTGAGAAATACTTTAATTGAGAAATTAAATACACTGGTAAGCGGTAAAACTTCTCAAGGGGTACACAATGACCTTGATGAAGAGATCATCGGTAAAGGTGTGAAATTCACTCACAAGTTATTAACTTCAGTTGAAGATTATGTAAACGTTAGCGGTTCAGACTGGACAGTAGACGCTGACAAGAATGAATTGATCAAACAATTGATTCACAACTACAAAATCAAATATAACGACATCCAGGGAGTTAAAAACCGTGAGAAATTTGCTATTTCAATCGGAGACGAGCTTCCGGCAGGTATCATGAAGCTGGCTAAAGTTTACATCGCTAAGAAACGTAAACTGAATGTAGGGGATAAGATGGCAGGACGTCACGGTAATAAAGGTATCGTATCAAGAATCGTTCGTGAAGAAGATATGCCATTCCTTGAAGACGGAACACCGGTAGATATCGTATTGAATCCACTTGGGGTACCTTCACGTATGAACATCGGACAGATTTATGAAACAGTTCTTGGATGGGCTGGTCAGAAACTGGGAATGAAGTTCGCTACACCAATCTTTGACGGAGCAACTCTTGATCAGATTACTGAATATACAGACAAGGCTGGTCTTCCTAAATTCGGTCACACTTACCTTTATGATGGTGGTACCGGAGAAAGATTTACTCAGGCTGCGACTGTAGGTGTTATTTACATGCTGAAACTAGGACACATGGTTGATGACAAAATGCACGCGCGTTCTATTGGTCCTTACTCATTGATTACTCAGCAGCCGTTAGGAGGTAAGGCTCAGTTCGGAGGTCAGAGATTCGGAGAGATGGAGGTTTGGGCACTTGAAGCATTCGGTGCATCTAACATCCTGAGAGAAATCCTGACTGTGAAGTCGGATGACGTGATTGGTAGAGCAAAAACTTATGAAGCAATTGCTAAGGGTGAATCTATGCCTGAACCAGGTATTCCGGAATCATTCAACGTATTACTTCACGAGTTACAAGGTCTTGGACTAGACGTAAGAATCTTGCTTCTGGTATTGTGTTTTAGTCTCTTAATTGCTTTTTCTTTGATCTGACGAACTCTCTCTCTTGTAAGATCGAAAGTCTCACCAATTTCTTCTAAAGTCATTGGGTGTTTTCCGTTCAGTCCGAAATACAATCTTACTAAATCAGCCTCTCTTGGCGTCAAAGTATTCAATGCTCTTTCAATCTCGATTTGCAGAGATTCAAGCATCAGATCTTTATCAGGACTTGGAGATTCTCCTGAACGTAATACATCATAAAGATTAGAATCTTCACCTTCTACTAAAGGCGCATCCATAGACAGGTGTCTTCCGGAGTTTTTCATAGATTCTTTGATATCTTCCTCGCTCATATCAAGAACTTCAGCCAATTCTTCCGGAGAAGGTGGTCTTTCATTTTCCTGTTCAAGGTGAGCGTATGCTTTATTGATTTTGTTGATAGATCCAATCTTGTTCAATGGCAATCTTACAATTCTTGACTGCTCAGCCAATGCCTGTAAAATTGATTGACGGATCCACCATACTGCATAAGAGATAAATTTGAAACCTCTAGTTTCATCGTACCTTTTTGCCGCTTTCATTAATCCTAAGTTACCTTCATTGATCAAATCGGGTAAAGAAAGACCCTGGTTTTGGTATTGCTTAGATACAGATACTACGAAACGAAGGTTGGCTTTGATTAATTTCTCAAGTGCGGCTCTGTCGCCGGCACGTATTCTTTGTGCCAATTCTACTTCCTCGTCCGCAGTAATCAGTTCCACTTTACCAATTTCCTGCAAATACTTGTCTAATGAAGCAGTTTCCCTGTTGGTAACCTGCTTAGTGATTTTTAATTGTCTCAT

>Chryseobacterium_haifense_DSM 19056T

AGCATTCAGGCATTGGAAGGAATGGAGCACGTGAGAATGCGTCCGTCCATGTACATTGGTGATGTAGGTCTTAGAGGACTTCACCATTTGGTTTATGAAGTAGTAGATAACTCTATCGATGAAGCTTTAGCAGGCTATTGTGACTCGATTACCGTTACCATCAAAGAAGGTAATGCCATAGAAGTAACCGATAACGGGCGTGGAATCCCAGTAGATTTACACGAAAAAGAACAAAAATCGGCTTTGGAAGTCGTGATGACGAAGATTGGTGCAGGAGGAAAATTTGATAAAGATTCCTATAAAGTTTCCGGAGGTCTGCACGGAGTTGGGGTATCGTGTGTGAATGCACTTTCTAATGTAATGATTACCACAGTTTACCGTGACGGAAACATCTACCAACAAACCTATTCTAAAGGTAAAGCACAAACCGAAGTAACCGAAATTGGACACAGCGACAAAA-GAGGAACCAAACAATTCTTCCAACCCGATGATACCATTTTCACCGAA---TTAGTTTACAATTACGATACTTTAGCGAGCAGATTAAGAGAGCTTTCTTACCTAAACAAAGGTATTACGATCACTTTAACTGATGAAAGAGAAAAACTGGAAGACGGAAGTTTCAAATCTGAAATCTTCCACTCTGAAGGCGGTTTAAAAGAATTCGTAGAATTTATCGACGGTAACCGCGAATCGATCATGAGCAATGTGATCTTCATGGAAGGTGAAAAAGATGATATCCCGGTAGAAGTTGCGATGCGATACAACACTTCTTATAACGAAAATCTTCACTCATACGTCAACAATATCAACACCCATGAAGGTGGAACTCACCTAGCCGGTTTTAGAAGAGCTTTAACGAGAACTTTGAAAAAATTTGCTGACGAATTAGGAATCCCTGCTAAAGAAAAAGTAGAAGTTACCGGTGATGACTTTAGAGAAGGTTTAACTGCCGTGATTTCAGTAAAAGTAATGGAACCACAGTTCGAGGGACAAACCAAAACCAAACTCGGTAACTCCGAAGTTTCGGGAGCGGTAGATAAAATCGTAGGCGAAATGCTTACCAATTTCTTGGAAGAAAACCCTAACGAAGCGAAAATCATCGTACAGAAAGTTGTTTTAGCAGCAAAGGCAAGACAAGCAGCGAAAAAAGCAAGAGAACTAGTTCAAAGAAAATCCCCAATGGGCGGAAGCGGACTTCCCGGAAAACTTTCTGATTGTTCATCGAAAGATCCGGAAATTTCCGAATTATTCCTCGTGGAGGGAGATTCCGCAGGTGGTACAGCTAAACAAGGCCGAGACCGTCATTTCCAGGCAATTCTTCCTTTAAGAGGTAAAATTTTGAACGTTGAAAAATCAATGCTTCACAAGGTTTATGACAATGAAGAAATCAAAAACATCTACACCGCTCTTGGCGTTTCTGTAGGTACTGAAGAAGACTCCAAAGCACTCAATATTTCCAAACTACGTTATCACAAAATCGTGATTATGACCGATGCGGATATTGATGGATCGCACATTTCTACGTTGATTTTAACGTTCTTCTTCCGTTATATGAAAGAACTCATCGAAAACGGATACATTTATATCGCACAACCACCACTCTATTTGTTGAAAAAAGGAAACAAAAAAGTGTACGCCTACAACGAGAAGGAGCGCGAAGACCTTACTTCGGAAATGGCTCCGGACGGAAAAGGTGTGGAAGTTCAACGATACAAAGGTCTTGGGGAAATGAATCCTGAACAACTTTGGGAAACTACATTGAACCCAGAAAATAGAATCTTGAAACAAGTTACCATTGAAAGTTTAGCCGATGCTGACAACGTCTTCTCGATGTTAATGGGCGATGAAGTACCTCCAAGAAGAGAATTCATTGAGAAAAATGCAATCTACGCAAAAATTGATGAATTCTCTATCATCCAAAATACTTTACAAAAAGACCCTACCAACTCCGAAAAAGAAGCGGTGGAGTATATCTATCGTCAGTTGCGTAACGCAGATCCGCCAGATGAGGAAACTGCAAGAGGAATTATCGAAAAATTATTCTTCTCAGAACAGCGTTATTCATTAGGAGAAGTTGGTCGTTACAGATTGAACAAGAAATTAGGATTAAATATTCCTGAAAAAACGGAAGTTTTAACCAAAGAAGACATCATTTCCATCGTTCGTCACTTGATCGAATTGGTAAACTCCAAAGCGGAAGTTGATGATATCGACCACTTGTCAAACAGAAGAATTAAAACTGTTGGTGAGCAATTGGCAGGACAATTCGGTGTTGGTCTTTCAAGAATTGCAAGAACAATCCGTGAGAGAATGAACGTTAGAGATAACGAAATCTTTACTCCAATTGATCTTGTTAATGCTAAAACTTTAACATCTGTAATCAACTCATTCTTTGGTACCAACCAGCTTTCTCAGTTCATGGACCAAACCAACCCGCTATCAGAAATCACGCACAAGCGTCGTCTTTCTGCACTAGGACCTGGTGGTTTATCAAGAGAAAGAGCAGGTTTCGAGGTGCGTGACGTTCACCATACTCACTATGGTCGTATCTGTCCGATCGAAACTCCTGAAGGACCAAACATTGGTTTGATTTCTTCTTTAGGAATCTATGCAAAAATCAACAACTTAGGTTTCATCGAAACTCCTTACCGTAAAGTAAACAACGGAAAAGTAGATTTGAAAACTCCTGCAGTTTTCTTAAACGCAGAAGATGAGGAAGATAAAGTTATTGCTCAGGCGAACGTTGAAATGAACGAAGACGGAACAATTTCTACAGACCGTGTAATTGCACGTCTTGATGGTGATTATCCGGTAGTTGAACCTCAACAGGTTGATTTGATCGATGTGGCACCGAACCAGATTTCTGGTATTTCCGCTTCATTAATTCCGTTCTTGGAGCATGATGATGCGAACCGTGCATTGATGGGATCCAACATGATGCGTCAAGCTGTTCCTTTGTTGAAGCCACAAGCTCCAATCGTAGGTACTGGCTTGGAACAACAAGTTGCTAAAGACTCCAGAATTTTGATTAATGCTGAAGGAAACGGTACTGTAGAGTATGTAGATGCGGATAAAATCACCATTAAATACGAAAGAAGCGAAGACGATGATTTGGTGTCATTCGAATCTGCTACGAAAACTTATAAACTGACCAAGTTCAGAAAAACCAACCAAAGTACAACCATCACTTTGAGACCAAACGTAAGAGTAGGCGACAAAGTTCAAAAAGGTCAGGTACTTTGCGACGGTTATGCAACCGAAAACGGAGAGTTGGCATTAGGTAGAAACCTTGTGGTGGCCTTCATGCCTTGGAAAGGGTATAACTTTGAGGATGCGATCGTAATCAACGAGAAAGTAGTTCGTGAGGACTGGTTTACTTCGATCCACGTGGATGAATATTCATTGGAAGTTCGTGATACCAAATTAGGTATGGAAGAATTGACAGCAGATATTCCAAACGTTTCTGAAGAGGCAACCAAAGATCTTGATGAAAACGGTATGATCCGTATTGGAGCTGACGTGAAACCTGGAGATATTATGATTGGTAAAATCACTCCAAAAGGTGAATCTGATCCAACTCCAGAAGAAAAACTATTGAGAGCGATCTTCGGTGACAAAGCCGGTGATGTGAAAGATGCTTCTTTGAAAGCTGATTCATCATTAAGAGGGGTTGTAATTAACAAGAAATTGTTCTCTAGAAACATCAAAGACAAAAAGAAAAGATCCGAAGAGAAACTGAAACTTGAGGAAATCGAAAATACGTACAAAGCGAAATTCGATGAATTAAGAAATACCCTTCTCGAAAAATTAGGAACTCTGGTGAACGGTAAAACTTCACAAGGAGTGAAAAATGATCTTGATGAAGAAATCATCGGAAAAGGAGTGAAATTTACGACTAAATTACTTCAAAGTGTTGAAGATTATGTAAACGTAAGTGGAGCAGACTGGACCGTAGATGCCGATAAAAATGAATTGGTTAAACAGTTGATTCACAACTATAAAATCAAATTCAACGATATTCAAGGGGTTAAAAACCGTGAGAAATTTGCAATCTCAATTGGTGATGAACTTCCTGCAGGAATTATCAAACTTGCAAAAGTTTATGTTGCTAAGAAACGTAAACTGAACGTAGGTGATAAAATGGCAGGACGTCACGGTAACAAAGGGATCGTTTCCCGTATCGTTCGTGAAGAAGATATGCCATTCCTAGAAGACGGAACTCCTGTTGATATCGTGCTGAATCCACTTGGGGTACCTTCACGTATGAACATCGGGCAGATCTACGAAACTGTTCTTGGATGGGCTGGTCAGAAATTAGGATTGAAATTCGCTACACCGATCTTCGATGGGGCTAATATCGATCAAATTACCGAGTACACCAATCAGGCAGGTCTTCCGGAATTCGGGTCAACTTATCTATATGATGGTGGAACTGGCGAGAGATTCTCTCAACCGGCAACTGTTGGGGTAATTTACATGCTGAAACTCGGTCACATGGTTGATGACAAAATGCACGCACGTTCTATCGGACCTTATTCATTGATTACGCAGCAGCCACTTGGTGGTAAAGCGCAGTTTGGTGGACAAAGATTCGGTGAGATGGAGGTTTGGGCACTCGAAGCATTCGGTGCATCCAATATCCTTAGAGAAATCCTTACCGTGAAATCGGATGACGTGATTGGTAGAGCGAAAACTTATGAAGCAATCGCGAAAGGTGAAGCAATGCCTGAACCAGGTATTCCGGAATCTTTCAACGTATTACTTCACGAGTTGCAAGGTCTTGGCCTTGATATAAGAATCTTACTTCTGGTATTGTGTTTCAAGCGTTTGATTGCTTTTTCTTTAATCTGACGAACTCTTTCTCTTGTAAGATCGAAAGTTTCACCGATTTCTTCTAAAGTCATTGGATGTTTCCCGTTCAAACCGAAGTATAAACGTACCAAATCAGCTTCACGAGGCGTTAAAGTTTGCAACGCTCTTTCGATTTCGATCTGAAGAGATTCCAGCATCAAATCTTTATCCGGACTTGGCGATTCCCCGGACCGCAAAACGTCATATAAATTGGAATCTTCACCTTCAACCAATGGCGCATCCATCGACAAGTGTCTTCCGGAGTTTTTCATTGATTCTTTGATGTCGTCTTCGCTCATATCCAAAACTTCCGCCAATTCTTCCGGAGAAGGAGGTCTTTCGTTTTCTTGCTCCAGGTGAGCGTAAGCTTTGTTGATTTTATTGATAGAACCAATTTTATTTAAAGGCAATCTTACAATTCTGGATTGTTCTGCCAATGCTTGCAAAATCGATTGGCGAATCCACCAAACGGCATATGAAATAAATTTAAAACCTCTGGTTTCATCATATCTTTTAGCAGCTTTCATCAAACCAAGATTCCCTTCGTTGATCAAATCGGGTAGGGAAAGTCCTTGGTTTTGGTATTGTTTCGAAACAGAAACCACGAAACGTAAGTTGGCTTTAATTAGTTTTTCAAGGGCAACTCTATCGCCAGCGCGAATTTTTTGTGCCAATTCTACTTCTTCATCAGCGGTGATAAGGTCCACTTTACCAATTTCTTGTAGATACTTGTCGAGTGAAGCGGTTTCTCTGTTGGTAACCTGCTTGGTAATTTTTAATTGTCTCAT

>Chryseobacterium_hispanicum_KCTC 22104T

AGTATTCAGGCATTGGAAGGAATGGAGCACGTTCGTATGCGTCCTTCGATGTACATTGGTGATGTAGGAGTCAGAGGTCTCCACCATTTGGTTTATGAAGTAGTGGATAACTCTATCGATGAGGCTTTGGCAGGATATTGTGATACGATAACTGTCGCGATAAAAGAAGGTAACGCCGTAGAAGTTATGGATAACGGTCGTGGTATTCCTGTCGATTTTCACGAGAAAGAACAGAAATCTGCGCTTGAGGTGGTAATGACCAAAATTGGAGCGGGTGGAAAGTTCGATAAAGATTCTTACAAAGTTTCCGGAGGTCTTCACGGTGTTGGGGTTTCGTGTGTTAACGCACTTTCCAACGAGATGATTACAACTGTTTACAGAGACGGAAACATCTATCAACAAGTTTATTCAAGAGGGAAAGCGCAAACAGGTGTTGAAGAAATCGGAAACAGCGATTACA-GAGGAACGAAGCAGTTTTTCCAACCGGATGATTCTATTTTTACTGAA---TTAGTTTACAATTACGATACATTGGCAAACCGTCTTAGAGAATTGTCTTACCTTAATAAAGGAATCACGATTACTTTAACAGACGAGAGAGAAAAATTGGAAGATGGCTCTTTCAAATCGGAAACTTTCCATTCAGAAGGTGGTTTAAAGGAATTCGTTGAGTATATCGATGGAAACCGCGAATCTATTATGGAGAACGTCATCTTTATGGAAGGCGAGCGCGATGATATTCCGGTTGAGGTGGCAATGCGTTACAACACGTCTTTCAATGAGAATCTTCACTCTTATGTTAATAATATCAATACACACGAAGGAGGAACACACTTAGCTGGTTTTAGAAGAGCTTTGACAAGAACTCTTAAGAAATATGCTGATGAATTAGGTCTTCCTGCAAAAGAAAAAGTTGAAGTTACAGGAGACGATTTCCGTGAAGGTTTGACGGCTGTGATTTCTGTAAAAGTAATGGAGCCTCAGTTCGAGGGTCAAACTAAAACGAAATTAGGAAACTCAGAAGTTTCGGGAGCGGTTGATAAAATCGTTGGAGAAATGTTGACTAATTTCTTGGAAGAAAATCCATTAGAAGCGAAACAAATCGTACAAAAAGTGGTTTTAGCTGCAAAAGCTAGACAAGCTGCGAAAAAAGCGCGTGAAATGGTTCAGAGAAAATCTCCAATGGGAGGTTCTGGCCTACCAGGGAAGTTATCTGACTGTTCTTCTAAAGACCCAGCTGAATCTGAAATCTTTCTTGTTGAGGGAGATTCCGCAGGTGGAACGGCTAAGCAAGGTCGTGACCGTCATTTCCAAGCGATTCTTCCATTGAGAGGTAAGATTTTGAATGTTGAGAAATCAATGCTTCACAAGGTTTATGATAACGAGGAAATCAAGAATATATACACAGCACTTGGGGTTTCTGTAGGAACAGAGGAAGATTCTAAAGCGTTGAATATTACAAAACTTCGTTATCACAAAATCGTGATTATGACCGATGCTGATATCGATGGTTCTCACATCTCAACTTTGATTCTGACTTTCTTCTTCAGATATATGAAAGAATTGATTGAGAACGGATACATTTATATCGCTCAACCGCCTTTATATTTATTGAAAAAAGGAAACAAAAAAGTTTACGCTTATAACGAAAAAGAACGTGAAGAGTTTACTCTGGAGATGTCACCAGATGGAAAAGGAGTAGAGGTTCAGCGTTACAAAGGTCTTGGAGAGATGAATCCTGAACAGCTTTGGGAAACTACTCTAAATCCTGAACACAGAATCTTGAAACAAGTTGCAATCGACAATGCCGTAGAAGCAGATTCTACTTTTTCTATGTTGATGGGCGACGAAGTTCCGCCGAGAAGAGAATTCATTGAAAAAAATGCGGTTTACGCAAGAATTGATGAGTTCTCTATCATCCAAAATACTTTACAAAAAGATCCAACCAACTCAGAAAAAGAAGCGGTTGAATATATCTACCGTCAGTTAAGAAATGCTGATGCACCGGATGAGGAAACTGCAAGAGGAATTATTGAAAAATTATTCTTCTCAGAGCAGAGATATTCGTTAGGTGAAGTTGGTCGTTATAGATTGAACAAAAAATTAGGTCTTAATATTTCTCAAGAAAACCAAGTGTTGACGAGAGAAGATATCATCTCTATCGTAAAACACTTGATTGAGTTGGTAAATTCTAAGGCTGAAGTTGATGATATCGATCACTTGTCAAACAGAAGAATCAAGACTGTAGGTGAGCAATTGTCTGGACAGTTTGGTGTAGGTCTTTCTAGAATTGCTAGAACTATCAAAGAAAGAATGAACGTTAGAGATAACGAAATCTTTACACCAGTTGATTTGGTTAATGCTAAGACATTGACATCTGTTATTAATTCATTCTTTGGTACCAACCAGCTTTCTCAGTTTATGGATCAAACCAACCCACTATCAGAAATCACGCATAAGCGTAGACTTTCTGCACTAGGGCCTGGTGGTTTATCTAGAGAAAGAGCAGGTTTCGAGGTTCGTGACGTTCACCATACTCACTATGGTCGTATCTGTCCAATCGAAACTCCAGAGGGACCAAACATTGGTTTGATCTCTTCACTTGGGATGTTTGCAAAAATCAACCGTCTTGGATTTATAGAAACGCCTTATAGAAAAGTTGAAGAAGGTAAAGTTGATTTAGAGTCAGCTCCTACTTATCTTAATGCAGAGGATGAGGAAGATAAAGTAATTGCTCAGGCAAACGTTGAGATGAACGATGATGGAAGCATCAGTACAGATAGAGTTATTGCTCGTCTAGATGGAGATTATCCTGTAGTTGAGCCTCAACAAGTAGACTTGATTGATGTGGCGCCAAACCAAATTTCTGGTATTTCAGCTTCATTGATTCCGTTCTTGGAGCATGATGATGCGAACCGTGCATTGATGGGATCTAACATGATGCGTCAGGCTGTTCCATTATTGAAACCTCAAGCACCAGTTGTAGGTACAGGTTTGGAAAAACAAGTAGCTAGAGACTCAAGAGTTTTGATCAACGCAGAAGGTTCTGGAGTTGTAGAATATGTGGATGCTGAGAAAATTGTTATTAAGTACGAAAGAAGCGAGGAAGACGACTTAGTTAACTTCGATTCTGCTACTAAAACTTATAAACTAACTAAGTTCAGAAAAACCAACCAGAGTACAACTATTACATTGAGACCAAACGTAAGAGTAGGTGATGTAGTAGAAAAAGGTCAGGTTCTTTGCGACGGTTATGCAACTGAAAACGGAGAGTTAGCATTGGGTAGAAACCTTGTGGTAGCCTTCATGCCTTGGAAAGGTTACAACTTTGAGGATGCTATCGTGATTAATGAAAAAGTAGTTCGTGAGGACTGGTTTACATCAATCCACGTTGACGAATATTCTCTTGAAGTTCGTGATACCAAATTAGGTATGGAAGAATTGACAGCTGATATCCCTAACGTTTCTGAAGAAGCTACCAAAGATCTTGATGAGAATGGTATGATACGTATCGGAGCAGAAGTGAAGCCTGGTGATATCCTTATTGGGAAAATTACACCAAAAGGGGAGTCTGATCCAACGCCAGAAGAAAAACTTCTTAGAGCAATCTTCGGAGATAAAGCTGGTGATGTAAAAGATGCTTCGTTGAAAGCAGATTCTTCATTAAGAGGAGTTGTAATCAACAAAAAATTGTTCTCTAGAAATATCAAGGACAAAAAGAAAAGAAGCGAAGAGAAAATCAAGTTAGAAGAAATCGAAAATACTTACAAAGCTAAGTTCGATACTCTTAGAAATACTTTGATTGAAAAATTGAATACTCTAGTTTCTGGTAAAACGTCTCAAGGTGTACAAAATGATCTTGAAGAAGAAGTAATCAGTAAAGGAACTAAATTCACATTGAGATTACTTCAAAACGTTGAAGATTATAACAACATTAGTGGTGCAGATTGGACAGTTGATTCTGATAAAAATGAATGGATCAAACAATTGATTCACAATTATAAAATCAAATACAACGATCTTTCTGGTGTTAAAAACCGTGAGAAATTTGCATTGTCTATCGGAGACGAGTTACCAGCAGGTATCATCAAACTAGCTAAAGTTTACATCGCTAAGAAACGTAAACTGAATGTTGGTGATAAAATGGCGGGTCGTCACGGTAACAAAGGTATCGTTTCAAGAATCGTACGTGAAGAAGACATGCCATTCTTAGAAGACGGAACACCAGTAGATATCGTATTGAATCCACTTGGGGTACCTTCTCGTATGAACATTGGTCAGATCTACGAAACTGTTCTTGGTTGGGCTGGAACTAAGCTAGGACTTAAGTTTGCAACACCAATCTTTGATGGAGCAGAACTAGATGAGATTACCGAATATACAGATAAAGCAGGTCTTCCTAAATTCGGTCACACACACCTTTACGATGGTGGTACAGGAGAGAGATTTACTCAAGCAGCTACAGTTGGTGTGATTTACATGTTGAAACTAGGACACATGGTAGATGATAAGATGCACGCACGTTCTATCGGACCATACTCATTGATTACGCAACAGCCGTTAGGAGGTAAAGCACAATTTGGAGGTCAGAGATTTGGAGAGATGGAGGTTTGGGCTCTTGAAGCATTTGGAGCATCTAACATCCTTAGAGAAATCTTGACTGTTAAGTCAGATGACGTGATTGGTAGAGCAAAAACTTATGAAGCGATTGCGAAAGGAGAAGCAATGCCAGAACCTGGTATTCCAGAATCTTTCAATGTATTACTTCACGAGTTGCAAGGTCTTGGACTTGATGTAAGAATCTTACTTCTAGTATTATGTTTCAATCTCTTAATTGCTTTTTCTTTGATTTGACGAACTCTCTCTCTCGTAAGGTCAAAAGTTTCACCAATTTCTTCAAGAGTCATTGGGTGTTTTCCGTTCAATCCGAAATATAATCTTACAAGATCAGCTTCTCTCGGCGTCAAAGTCTGAAGCGCTCTTTCAATCTCGATTTGTAGAGACTCCAACATCAAATCTTTATCAGGACTTGGAGACTCACCAGAACGTAAAACATCATAAAGGTTAGAATCTTCACCTTCCACAAGTGGCGCATCCATAGAAAGGTGACGACCACTGTTTTTCATAGATTCCTTGATGTCTTCCTCGCTCATATCAAGAACTTCTGCCAATTCTTCCGGAGAAGGAGGTCTTTCGTTCTCTTGCTCAAGGTGAGCATAAGCTTTGTTGATTTTGTTGATAGAACCAATTTTGTTCAAAGGTAATCTTACAATTCTCGACTGCTCCGCCAAAGCCTGCAAAATCGATTGACGAATCCACCAAACTGCATAAGAGATGAATTTGAAACCTCTCGTTTCATCATATCTTTTAGCCGCTTTCATCAGACCAAGATTTCCTTCGTTGATTAGATCGGGAAGAGAAAGACCTTGATTTTGATACTGCTTAGAAACCGAAACCACGAAACGCAAGTTAGCTTTGATCAATTTTTCAAGAGCAGCTCTATCGCCGGCACGGATTCTCTGAGCCAAATCTACCTCCTCGTCAGCGGTAATCAATTCAACTTTTCCAATCTCTTGCAGATACTTGTCTAGTGATGCGGTTTCCCTGTTGGTAACCTGTTTTGTAATTTTTAATTGTCTCAT

>Chryseobacterium_hominis_DSM 19326T

AGTATTCAGGCATTGGAAGGAATGGAGCACGTTCGTATGCGTCCTTCGATGTACATTGGTGATGTAGGAGTCAGAGGTCTCCACCATTTGGTTTATGAAGTAGTGGATAACTCTATCGATGAGGCTTTGGCAGGATATTGTGATACGATAACTGTCGCGATAAAAGAAGGAAATGCCATAGAAGTTATGGATAATGGGCGTGGTATTCCTGTCGATTTTCACGAGAAAGAACAGAAATCTGCACTGGAGGTTGTAATGACCAAAATTGGAGCCGGTGGTAAGTTTGATAAAGATTCTTACAAAGTTTCCGGAGGTCTGCATGGTGTTGGGGTTTCTTGCGTTAACGCTCTTTCCAATGAGATGGTTACAACGGTCTATAGAGACGGTAATGTTTATCAGCAAGTTTATTCCAGAGGGAAAGCTCAGACAGGCGTTGAAGAAATAGGTCATAGTGATAAAA-GAGGAACCAAGCAGTTTTTCCAGCCAGACGATTCTATTTTCACAGAA---TTAGTTTATAACTACGATACATTAGCAAATCGTCTTAGAGAATTGTCTTACCTTAATAAAGGAATCACGATTACTTTGACAGATGAGAGAGAGAAATTGGAAGATGGTTCTTTCAAGTCAGAAACTTTCCATTCGGAAGGTGGTTTAAAGGAATTCGTTGAGTATATCGATGGAAACCGTGAGTCCATTATGGAGAACGTCATCTTTATGGAAGGCGAACGTGATGATATTCCGGTTGAGGTAGCGATGCGTTATAACACATCTTTCAATGAGAATCTTCATTCTTATGTTAATAATATTAATACGCACGAAGGAGGTACTCACTTGGCAGGTTTCAGAAGAGCTTTGACAAGAACTCTTAAGAAATATGCTGATGAATTAGGACTTCCTGCAAAAGAAAAAGTAGAAGTTACCGGAGATGATTTCCGTGAAGGTTTGACGGCGGTTGTTTCTGTAAAAGTAATGGAGCCTCAGTTCGAAGGTCAGACTAAAACGAAATTAGGTAACTCAGAAGTTTCAGGAGCGGTTGATAAAATCGTTGGAGAAATGTTAACTAATTTCTTGGAAGAGAATCCGTTAGAAGCGAAACAAATCGTACAAAAAGTTGTTTTAGCTGCGAAAGCTAGACAAGCTGCGAAAAAAGCGCGTGAAATGGTTCAGAGAAAATCTCCAATGGGAGGTTCTGGTTTACCAGGGAAATTATCTGATTGTTCTTCTAAAGACCCAGCTGAATCTGAGATTTTCCTAGTAGAGGGAGATTCTGCAGGTGGAACGGCAAAACAAGGTCGTGACCGTCATTTCCAGGCAATTCTTCCATTGAGAGGTAAGATTTTGAATGTTGAGAAATCGATGCTTCATAAGGTTTATGATAATGAGGAAATCAAAAATATCTATACAGCGCTTGGAGTTTCTGTAGGAACAGAAGAAGATTCTAAAGCGTTGAATATGGCGAAATTGAGATATCACAAAATCGTGATTATGACCGATGCCGATATCGATGGTTCTCACATTTCGACATTGATTCTGACTTTCTTCTTCCGTTATATGAAAGAACTGATCGAGAATGGATATATTTATATCGCTCAGCCACCTTTGTATCTTTTGAAAAAAGGAAACAAAAAAGTTTACGCTTATAACGAAAAAGAACGTGAGCAATTTACCTTGGAAATGTCGCCAGACGGAAAAGGTGTGGAAGTACAACGTTACAAAGGTCTTGGAGAGATGAATCCTGAACAGCTTTGGGAAACGACACTCAATCCAGAACACAGAATTCTGAAACAGGTAACAATTGATAATGCCGTTGAAGCAGATTCTACATTCTCTATGCTGATGGGTGATGAAGTACCACCTAGAAGAGAATTTATCGAGAAAAATGCGGTTTACGCAAGAATTGATGAATTCTCTATCATCCAGAATACACTACAAAAAGACCCGACTAACTCTGAAAAAGAAGCAGTGGAGTACATCTACCGTCAGCTGAGAAACGCTGATGCACCGGATGAGGAAACTGCAAGAGGAATCATTGAAAAATTATTCTTCTCTGAGCAGCGTTATTCTTTGGGTGAAGTTGGTCGTTACAGATTAAATAAAAAATTAGGTCTTAACATTCCGGAAACTACGGAAGTACTGACCAAGGAAGATATCATTGCAATTGTAAAGCACCTGATCGAGTTGGTTAATTCCAAAGCGGAGGTAGATGATATTGACCACCTTTCTAACAGACGTATCAAAACTGTGGGCGAGCAATTGTCTGGTCAGTTTGGTGTCGGTCTTTCCAGAATCGCAAGAACTATCAAGGAAAGAATGAATGTTAGAGATAACGAGATCTTTACACCGGTAGATTTGGTTAATGCCAAGACTTTGACGTCTGTTATTAATTCATTCTTCGGAACCAACCAGCTTTCTCAGTTTATGGATCAGACCAATCCGTTGTCAGAGATCACGCACAAGCGTAGATTATCTGCACTAGGACCTGGTGGTTTATCAAGAGAAAGAGCAGGTTTCGAGGTGCGTGACGTTCACCATACGCACTACGGACGTATCTGTCCGATTGAAACGCCGGAAGGACCAAACATCGGTTTGATCTCTTCTCTTGGGATGTATGCGAAAATCAATAACCTTGGTTTTATCGAAACGCCTTACAGAAAAGTAGAGGACGGAAAAGTAGATCTTCAGGCTAAGCCAATTTATCTAAATGCAGAGGATGAAGAAAATAGAGTAATTGCTCAGGCTAACGTTGCTTTGAGTGATGATGGTAACTTCGAAACGGATAGAATTATCGCTCGTTTGGATGGTGACTATCCTGTAGTAGAGCCTCAGCAAGTAGATTTGATTGACGTTGCGCCAAACCAGATTTCCGGTATTTCCGCTTCATTGATTCCTTTCTTGGAACACGATGATGCGAACCGTGCTTTGATGGGATCTAACATGATGCGTCAGGCCGTTCCATTATTGAAGCCACAAGCGCCAATCGTGGGTACAGGTCTGGAAAAGCAAGTTGCGAAAGATTCTAGAATTCTTATCAATGCAGAAGGAAATGGAGTAGTGGAATATGTGGATGCTGAAAGAATTGTTATTAAGTATGAAAGAAGCGAAGAAGATGATTTGGTAAACTTCGATTCTGCAACTAAAACTTATAAACTGACTAAGTTCAGAAAAACCAACCAGAGTACAACTATTACATTGAGACCAAATGTAAGAGTAGGTGATACAGTGGAAAAAGGACAGGTGCTTTGTGACGGTTATGCAACTGAAAACGGGGAATTAGCTCTTGGTAGAAACCTTGTAGTTGCCTTCATGCCTTGGAAAGGTTACAACTTCGAGGATGCGATTGTTATCAACGAAAAAGTAGTTCGTGAGGACTGGTTTACTTCAATCCACGTAGATGAGTATTCTCTTGAAGTTCGTGATACCAAATTAGGTATGGAAGAACTGACAGCAGATATTCCTAACGTTTCTGAAGAAGCGACTAAAGATCTTGATGAGAACGGTATGATCCGTATCGGTGCAGAAGTGAAGCCTGGTGATATCCTTATCGGGAAAATCACACCAAAAGGGGAATCTGATCCAACGCCGGAAGAAAAATTGCTTAGAGCAATCTTCGGAGACAAAGCTGGTGATGTAAAAGATGCTTCATTGAAAGCAGATTCTTCTCTTAGAGGTGTTGTTATCAACAAAAAATTGTTCTCCAGAAACATCAAGGACAAAAAGAAAAGAAGCGAGGAGAAAATTAAGTTGGAAGAAATCGAAAATACTTACAAAGCTAAGTTCGATGACTTACGAAACACTTTGATTGACAAACTAAACACGTTGGTTTCCGGTAAAACGTCTCAAGGGGTGAAAAATGACCTTGAAGAAGAAATGATCGGAAAAGGAACTAAGTTTACTTTGAAGTTGCTTCAGTCTGTTGAGGATTATGTAAACATCAGTGGTGCAGACTGGACTGTAGATGCAGACAAGAATGAATTGATCAAGCAATTGATTCACAACTATAAAATTAAATACAACGATCTTTCTGGTGTTAAAAACCGTGAGAAATTTGCATTATCCATCGGAGACGAGCTACCTGCTGGTATCATCAAATTAGCTAAAGTTTACATCGCTAAGAAACGTAAACTGAATGTGGGTGACAAAATGGCTGGTCGTCACGGTAACAAAGGTATTGTGTCAAGAATCGTTCGTGAAGAAGATATGCCGTTCTTAGAGGATGGAACACCGGTAGATATCGTATTGAATCCACTTGGGGTACCTTCCCGTATGAATATCGGTCAGATCTACGAAACTGTTCTTGGATGGGCAGGTAAGCAGCTTGGACTAAAATTCGCAACACCGATCTTCGACGGAGCTAGTTTAGATCAAATTACCGAATACACCGAGCAGGCAGCACTTCCTAAATTCGGACACACACACCTTTACGATGGTGGAACAGGGGAGAGATTTACTCAGCCAGCTACTGTTGGTGTGATTTATATGCTGAAGTTAGGACACATGGTAGATGATAAGATGCACGCACGTTCTATCGGACCGTACTCATTGATTACGCAGCAGCCATTAGGAGGTAAAGCGCAATTCGGAGGTCAGAGATTCGGAGAGATGGAGGTTTGGGCTCTTGAAGCATTTGGAGCATCCAATATTCTTAGAGAAATCTTGACTGTGAAGTCGGATGACGTGATTGGTAGAGCAAAAACTTATGAAGCAATCGCAAAAGGTGAAGCAATGCCAGAACCTGGTATTCCGGAATCTTTCAACGTATTACTTCACGAGTTACAAGGTCTTGGACTTGATGTAAGAATCTTACTTCTGGAGTTGTGTTTCAATCTTTTGATGGCTTTTTCCTTAATTTGTCTAACTCTTTCTCTCGTTAGGTCAAAAGTTTCCCCGATTTCCTCCAAAGTCATTGGATGTTTTCCGTTTAAACCGAAATATAATCTTACCAAATCTGCTTCTCTAGGCGTCAACGTGCAAAGTGCTCTTTCAATTTCGATTTGTAGAGATTCCAACATCAAATCTTTATCAGGACTTGGAGATTCTCCCGAACGCAAAACGTCATAAAGGTTAGAATCTTCACCTTCCACAAGCGGCGCATCCATAGAAAGGTGACGGCCACTGTTTTTCATAGATTCCTTGATGTCTTCCTCGCTCATATCCAGAACTTCTGCCAATTCTTCCGGAGAAGGTGGTCTTTCGTTTTCTTGCTCCAGGTGAGCATAAGCTTTGTTGATTTTGTTGATAGAACCAATCTTATTCAAAGGTAATCTTACAATTCTCGACTGCTCAGCCAAAGCCTGCAAAATCGATTGACGAATCCACCAAACAGCATACGAGATGAATTTGAAACCTCTTGTTTCATCATATCTTTTCGCCGCTTTCATCAGACCAAGATTTCCTTCGTTGATCAAATCGGGAAGAGAAAGTCCTTGATTTTGATATTGCTTAGAAACCGAAACCACGAAACGTAAGTTAGCTTTGATCAGTTTTTCAAGCGCAGCTCTGTCTCCGGCACGGATTCTCTGAGCCAAATCTACCTCTTCGTCAGCGGTAATCAATTCAACTTTTCCAATCTCCTGCAGATACTTGTCTAGTGATGCGGTTTCCCTGTTGGTAACCTGTTTTGTAATTTTTAATTGTCTCAT

>Chryseobacterium_humi_DSM 21580T

AGTATTCAGTCTTTAGAGGGAATAGAACATGTACGTTTGAGACCTTCCATGTACATTGGTGATGTGGGTGTGAGAGGACTTCACCATTTGGTTTATGAGGTAGTTGATAATTCCATTGATGAGGCTTTAGCAGGACATTGTGATACTATTACAGTTATAATTCATAAAGGGAATGGCATTACTGTAAAAGATAATGGACGTGGAATTCCCGTTGATTTACACGAAAAAGAACAAAAATCTGCCCTTGAAGTTGTAATGACAAAAATTGGCGCGGGTGGTAAATTTGATAAAGATTCTTACAAAGTTTCTGGTGGTTTACACGGTGTGGGAGTTTCAGTGGTAAATGCACTTTCTACATCTTTAATTGCGACTGTTTTCAGAGAAGGAAAAATTTATCAACAAAAATATTCAAAAGGTGCTGCTTTAGCAGACGTGCAAGAAATTGGTGTTTCTGACAAAA-GAGGAACAGAGGTTTTTTTCCAAGCAGATGGTTCTATTTTTAATGAA---TTAGAATTTAATTATAGCACACTTGCATCAAGATTAAGAGAATTGGCTTTTTTAAATAAAGGCATTACAATTACCTTAACTGATGAAAGAGAACCCAATGAAGATGGTTCTTTCCCAGTAGAAACCTTTCACTCAGATGGCGGTTTAAAGGAATTTGTGGAATATATTGACGGAAACCGTGAATCTATCATGGAAAAAGTAATTTTCATGGAAGGTGAAAGAGACGATATTCCGGTAGAAGTCGCGATGCGATACAATACTTCTTATACTGAAAACCTTCATTCTTATGTCAATAATATCAACACACATGAAGGCGGAACGCATCTAACTGGTTTTAGACGTGCGTTAACAAGAACTTTGAAAAAATTTGCTGATGAACTTGGTTTGCCTGCAAAAGAAAAAGTAGAAGTGACGGGTGATGATTTCCGTGAAGGTTTGACGGCAATTATCTCTGTGAAAGTAATGGAACCACAGTTTGAAGGACAAACCAAAACGAAATTAGGAAACTCTGAAGTTGCCGGTGCAGTGGATAAAATTGTGGGCGAAATGCTTTCTAATTTTTTAGAAGAAAATCCGAACGAGGCAAAAATTATTGTTCAGAAAGTAGTTTTAGCAGCAAAAGCAAGACAAGCGGCAAAAAAAGCCCGCGAAATGGTGCAAAGAAAATCACCAATGGGCGGAAGCGGTTTGCCAGGAAAACTATCCGATTGTACTTCTAAAGATCCAGCAGAAAGTGAATTATTTCTTGTAGAGGGAGATTCTGCAGGCGGAACTGCTAAACAAGGAAGAGATAGAATGTTTCAAGCGATTTTACCTTTAAGAGGGAAAATTTTAAACGTTGAAAAAGCAATGCTTCACAAGGTTTATGACAATGAAGAAATCAAAAATATTTATACCGCACTAGGCGTAAGCGTAGGAACAGAAGAAGATTCTAAAGAATTAAATATCAAAAAATTACGCTATCACAAAATAGTCATCATGTGTGATGCTGATATTGATGGCGCGCATATTTCTACTTTAATTTTGACTTTCTTCTTTAGATATATGAAAGAATTAATAGAAAATGGCTACATTTATATTGCATCACCGCCACTTTATTTATTAAAAAAAGGAAGCAAAAAAGTCTATACTTGGAATGAAAAAGATCGTGAGGAAGAAACTTACAAACTTTCTGCTGATGGAAAAGGAGTGGAAGTACAACGTTACAAAGGTCTTGGTGAAATGAATCCGGAGCAACTTTGGGATACAACTCTAAATCCAGACAATAGAATTTTAAAACAAGTAACTATTGATAATGCTGTAGAAGCAGATAACGTTTTCTCAATGCTGATGGGCGATGAAGTACCACCAAGACGTGATTTTATAGAGAGAAATGCGAAATATGCGAATATTGACGAGTTTTCTATTATTCAAAACACATTACAAAAAGATCCAACCAATTCTGAAAAAGAAGCCGTTGAATATATTTATAGACAATTAAGAAACGCAGATCCACCCGATGAAGAAACTGCACGCGGAATTATTGAAAAATTATTCTTCTCAGAGCAACGCTATTCTTTAGGTGAAGTAGGCCGCTATAGATTAAATAAAAAATTGAATCTAAAAATTGCCGATGACAAACAAGTTTTAACCAAAGATGATATCATTGCAATTGTAAGATATTTAATTCAGTTGGTGAATTCTAAAGCAGAGGTAGATGATATTGATCACTTATCAAACCGTAGAATTAAAACTGTTGGTGAGCAACTTTCCGGTCAGTTTGGTGTTGGTTTATCCAGAATCGCACGTACCATTAAAGAAAGAATGAACGTTCGTGATAATGAAATATTTACACCTACAGATTTAGTTAATGCTAAAACTTTAACTTCTGTAATTAATTCCTTCTTTGGAACCAACCAGTTGTCACAGTTTATGGATCAAACCAATCCACTTTCTGAAATTACACACAAAAGAAGACTTTCCGCACTAGGACCTGGTGGACTTTCACGAGAAAGAGCAGGTTTTGAGGTACGAGACGTTCACCACACGCACTACGGCAGAATTTGCCCGATTGAAACACCAGAGGGACCAAACATTGGTTTGATTTCTTCCCTTGGTATTTATGCAAAAATTAACAATCTTGGTTTCATTGAAACGCCTTATAGAAAAGTAACAAACGGTAAAGTAGAATTAAATTCTGCACCGATTTTCTTAAATGCAGAAGATGAAGAAGATAAAGTAATTGCGCAGGCGAATGTGGAAATGAAAGACGATGGAACAATTTCTACTGACCGTGTCATTGCTCGTTTAGATGGAGATTATCCAGTTGTGGAACCACAACAAGTTGATTTAATTGACGTTGCACCAAACCAAATTTCTGGTATTTCTGCCTCATTAATTACTTTCTTGGAGCATGATGATGCAAACAGAGCCTTGATGGGATCCAATATGATGCGTCAAGCCGTACCATTATTAAATCCAGAAGCACCAATCGTAGGGACTGGTTTAGAAAAACAAGTTGCAAAAGATTCTAGAATTTTAATTAATGCAGAAGGAAATGGTGTTGTAGAATATGTAGATTCAGATAAAATTATTATTAATTACGAATCAACTGAAAATGAAGATTTAGTTAGTTTTGATACTTCTGTTAAAGAATATAAATTAACAAAATTCCGTAAAACCAATCAAGGAACAACCATTACTTTAAGACCAAACGTAAGAGTAGGAGATAAAGTAGTTTTAGGACAAGTTCTTTGCGATGGCTATGCAACTGAAAAAGGTGAATTAGCACTTGGTAGAAACTTAAAAGTGGCATTTATGCCATGGAAAGGTTACAATTTTGAGGATGCAATCGTTATTAATGAAAAAGTAGTGCGTGAAGATTGGTTTACATCAATCCACATAGATGAGTATTCTCTAGAAGTAAGAGATACAAAATTAGGTATGGAAGAATTAACTTCCGACATCCCGAACGTTTCCGAAGAAGCAACCAAAGATTTAGACGAAAACGGTATGATCCGTATTGGTGCAGATGTAAAACCTGGTGATATTTTAATCGGGAAAATTACGCCAAAAGGCGAGTCTGACCCAACACCAGAAGAAAAATTGCTTCGTGCTATCTTTGGTGATAAAGCCGGCGATGTGAAAGATGCCTCTCTAAAAGCAGATTCTTCACTTCGTGGAGTTGTAATTAATAAAAAATTATTTACAAGAAACATCAAAGACAAAAAGAAAAGAAGCGAAGAGAAATTGAAACTTGAAGAAATCGAGAACGCATACAAAGCGAAAACCGATGAATTGAGAGCAGTTTTATTAGAGAAACTTAATACTTTGGTAAGCGGCAAAACATCACAAGGTGTTACCAATGATTTAGACGAAGAAGTAATTGGTAAAGGTGTAAAATTCACCCAAAAATTATTATCAAGCGTTGAAGATTATGTAAATATAAGTGGTGCAAACTGGACGGTTGATGCAGATAGAAATGAGTTGATTGTAAAATTAATTCACAACTACAAAATCAAATACAACGAAATACAAGGTGTGAAAAACCGCGAGAAATTCGCAGTTTCCATCGGTGATGAGTTGCCTGCAGGTATTATGAAATTAGCAAAAGTATACATTGCTAAAAAACGTAAATTAAATGTAGGTGATAAAATGGCCGGTCGCCACGGTAATAAAGGAATTGTTTCCAGAATTGTGCGCGAAGAAGATATGCCATTTTTGGAAGACGGAACCCCAGTTGATATCGTTTTAAATCCGCTTGGTGTACCTTCTCGTATGAACATTGGCCAGATTTACGAAACCGTTCTCGGATGGGCAGGTAAAGAGCTTGGGTTAAAATTTGCAACGCCAATTTTTGATGGTGCAAATATCGACCAGATTACAGAATATACCAAGCAAGCAGGTGTTCCAGAATTTGGAAGCACTTATCTTTATGATGGTGGAACCGGCGAGAGATTTACACAGCCTGCAACTGTTGGTATTATTTACATGTTGAAATTAGGACACATGGTAGATGATAAAATGCACGCAAGATCAATCGGGCCATATTCATTAATTACGCAGCAACCTTTAGGTGGTAAAGCGCAATTTGGTGGGCAAAGATTTGGAGAAATGGAAGTTTGGGCACTAGAAGCATTCGGTGCATCCAACATTTTAAGAGAAATCTTAACCGTGAAATCTGATGATGTGATTGGAAGAGCGAAAACTTACGAAGCCATCGCAAAAGGTGAAGCAATGCCAGAACCTGGTATTCCGGAATCCTTCAACGTATTGCTTCATGAGTTACAAGGTCTTGGTCTAGATATAAGAATCTTGCTTCTGGTATTGTGTTTCAAACGTTTTATGGCTTTTTCTTTAATCTGGCGAACGCGTTCTCTTGTTAGATCAAAAGTTTCACCGATTTCTTCTAAAGTCATCGGATGTTTCCCATTTAAACCAAAATAAAGACGAACCAAATCTGCCTCACGCGGCGTTAAAGTTTGCAATGCTCTTTCTATTTCAATTTGGAGAGATTCAAGCATTAAATCTTTATCCGGACTTGGCGATTCTCCAGAACGCAAAACATCATACAAATTAGAATCTTCACCTTCCACCAACGGCGCATCCATCGATAAATGGCGGCCAGAATTTTTCATAGATTCTTTGATGTCTTCTTCGCTCATATCCAAAACTTCCGCTAATTCTTCTGGTGAAGGTGGTCTTTCGTTTTGTTGCTCCAAATGAGCATAAGCTTTGTTGATTTTATTGATTGAGCCAATTTTATTTAAAGGCAAACGCACAATTCTAGATTGCTCTGCTAAAGCCTGCAAAATGGATTGACGAATCCACCAAACAGCGTAAGAAATAAATTTAAAACCTCTTGTTTCATCATATCTTTTGGCAGCTTTCATTAAGCCCAAATTTCCTTCATTAATTAAATCGGGAAGAGAAAGACCTTGGTTTTGGTATTGTTTAGAAACCGAAACCACGAAACGAAGATTGGCTTTAATTAATTTATTTAAGGCGATGGTATCACCTGCACGAATTTTCTGCGCTAAATTTACTTCTTCATCGGCTGTGATTAAATCTACTTTGCCGATTTCTTGTAAATATTTATCCAATGATGCGGTTTCCCTGTTGGTAACCTGTTTCGTAATTTTTAATTGTCTCAT

>Chryseobacterium_hungaricum_DSM 19684T

AGTATTCAGGCATTGGAAGGAATGGAGCACGTTCGTATGCGTCCTTCAATGTACATTGGTGATGTAGGAGTCAGAGGTCTCCACCATTTGGTTTATGAAGTAGTGGATAACTCTATCGATGAGGCTTTGGCAGGATATTGTGATACAATTACTGTTGCGATAAAAGAAGGGAACGCCGTAGAAGTTATGGATAACGGTCGTGGTATTCCTGTAGATTTTCACGAGAAAGAGCAGAAATCTGCGTTAGAGGTTGTAATGACCAAAATCGGAGCTGGTGGTAAGTTCGACAAAGATTCTTACAAAGTTTCCGGGGGTCTTCACGGAGTTGGGGTTTCGTGTGTTAACGCACTTTCCAACGAGATGATCACTACCGTTTACAGAGACGGAAACGTTTATCAGCAGGTTTATTCCAGAGGAAAAGCGCAAACCGGCGTTGAAGAAATAGGACATAGCGACAAAA-GAGGAACAAAACAGTTTTTCCAGCCGGATGATTCTATTTTTACTGAA---TTAGTTTACAACTACGATACTTTAGCAAACCGTCTTAGAGAGTTGGCATACCTTAATAAAGGAATCACGATTACTTTAACGGATGAAAGAGAAAAATTGGAAGATGGGACTTTCAGAACAGAAGTTTTCCATTCGGAAGGCGGATTGAAAGAATTCGTTGAGTATATCGACGGAAACCGTGAGTCTATTATGGAGAATGTGATCTTTATGGAAGGCGAGCGTGACGATATTCCGGTTGAGGTGGCGATGCGTTATAACACGTCTTTCAACGAGAATCTCCACTCTTATGTTAATAATATCAATACACACGAAGGAGGAACGCATTTGGCAGGTTTCAGAAGAGCTTTAACAAGAACTCTTAAGAAATATGCCGATGAATTAGGGCTTCCTGCAAAGGAAAAGGTTGAAGTTACTGGAGACGATTTCCGTGAAGGTTTGACGGCTGTGATTTCTGTAAAAGTTATGGAACCTCAGTTCGAGGGTCAGACCAAAACGAAATTAGGTAACTCGGAGGTTTCCGGTGCGGTTGATAAAATCGTGGGCGAAATGCTGACAAACTTTCTCGAGGAAAATCCTGCTGAAGCCAAGATTATCGTTCAGAAAGTTGTTTTGGCGGCAAAAGCAAGACAAGCGGCAAAGAAGGCAAGAGAAATGGTTCAGAGAAAATCTCCGATGGGAGGTTCTGGTTTGCCAGGAAAACTGTCCGATTGTTCTTCAAAAGATCCGGCTGAATCAGAATTGTTCCTTGTAGAGGGAGATTCCGCAGGTGGAACGGCTAAACAAGGTCGTGACCGTCATTTTCAGGCAATTTTGCCTTTGAGAGGTAAGATCCTGAATGTTGAGAAATCTATGGTTCATAAAGTTTATGATAACGAGGAGATCAAGAATATCTATACGGCGCTTGGCGTTTCTGTAGGAACAGAAGAGGATTCCAAAGCGTTGAATATGGCAAAACTTCGTTACCACAAAATTGTGATCATGACCGATGCCGATATCGATGGTTCTCACATCTCAACTTTGATTCTGACTTTCTTCTTCAGATATATGAAGGAATTGATTGAGAACGGATATATTTATATCGCTCAGCCGCCTTTATATCTATTGAAAAAAGGAAACAAAAAAACTTATGCATACAACGAGAAGGAGCGTGAACAATTCACTCTTGAAATGTCTCCGGACGGAAAGGGAGTAGAGGTACAACGTTACAAAGGTCTTGGAGAGATGAATCCTGAGCAGCTTTGGGAAACAACGCTGAATCCGGATGGCAGGATCCTGAAACAAGTAACCATTGACAATGCGGTAGAGGCAGATTCTACATTCTCTATGCTGATGGGTGATGAAGTTCCGCCGAGAAGAGAATTCATTGAGAAAAATGCGGTTTACGCAAGAATTGATGAGTTCTCTATCATCCAGAATACTTTACAAAAAGATCCAACCAACTCAGAAAAAGAGGCGGTGGAATATATTTATCGTCAGTTAAGAAACGCCGATGCTCCGGATGAGGAAACTGCAAGAGGAATTATTGAGAAATTATTCTTTTCAGAACAGAGATACTCTCTTGGAGAAGTTGGTCGTTACAGATTGAACAAAAAACTAGGTCTTAACATTTCGGAAGAAAATCAAGTTCTTACAAAAGAAGATATCATCTCGATTGTAAAACACTTGATCGAATTGGTTAACTCTAAGGCTGAGGTTGATGATATCGATCACTTGTCAAACAGAAGGATCAAGACTGTTGGAGAGCAATTGTCTGGACAATTCGGCGTAGGTCTTTCTAGAATTGCCAGAACAATCAAAGAAAGAATGAACGTTAGAGATAACGAGATCTTTACACCGGTTGATTTGGTTAATGCTAAGACTTTGACGTCTGTTATTAATTCATTCTTTGGTACCAACCAGCTTTCTCAGTTCATGGATCAAACCAACCCATTGTCAGAGATCACGCACAAGCGTAGACTTTCTGCACTAGGACCTGGTGGTTTATCAAGAGAAAGAGCAGGTTTCGAGGTTCGTGACGTTCACCATACTCACTATGGTCGTATCTGTCCAATCGAAACTCCGGAAGGACCAAACATTGGTTTGATCTCTTCTTTGGGTATGTATGCGAAGATCAACACTTTAGGTTTTATTGAGACACCTTACAGAAAAGTAGAAAACGGAAAAGTAGACCTTAATGCTGCGCCTGTTTATCTAAATGCTGAGGACGAGGAAAATCAAGTTATTGCTCAGGCAAACGTTGCCTTGAGTGATGAAGGTCACTTCGAGACGGATAGAATTATTGCCCGTTTGGATGGTGATTACCCAGTAGTTGAGCCTCAACAAGTCGATCTTATCGACGTTGCACCAAACCAGATCTCTGGTATTTCTGCTTCATTGATCCCGTTCCTGGAGCATGATGATGCGAACCGTGCATTGATGGGGTCTAACATGATGCGCCAGGCAGTTCCTTTATTGAAGCCTCAGGCACCAGTTGTTGGTACAGGTCTGGAAAAACAAGTTGCGAGAGATTCTAGAATTTTGATCAATGCAGAAGGAACTGGAGTTGTAGAATATGTGGACGCTGAGAAAATAGTTATTAAGTACGAAAGAAGCGAGGAGGATGATCTAGTTAACTTCGATTCTGCTACTAAAACTTATAAACTGACTAAGTTCAGAAAAACCAACCAGAGTACAACTATTACACTAAGACCAAACGTAAGAGTAGGTGATACAGTGGAAAAAGGTCAGGTTCTTTGTGATGGTTATGCAACTGAGAACGGGGAATTGGCTCTTGGTAGAAACTTGGTAGTGGCGTTCATGCCTTGGAAAGGTTATAACTTCGAGGATGCGATCGTAATTAATGAAAAAGTGGTTCGTGAGGACTGGTTTACTTCGATCCACGTTGATGAATATTCTCTGGAAGTTCGTGATACCAAATTAGGTATGGAAGAATTGACAGCAGATATTCCTAACGTTTCTGAAGAAGCTACAAAAGATCTTGATGAAAACGGGATGATCCGTATCGGTGCAGAAGTGAAGCCTGGTGATATCCTTATCGGAAAGATCACTCCAAAAGGGGAATCTGATCCAACTCCGGAAGAAAAACTTCTTAGAGCGATCTTCGGGGATAAAGCTGGTGATGTAAAAGATGCTTCATTGAAAGCAGATTCTTCTCTTAGAGGAGTTGTTATCAACAAAAAATTGTTCTCTAGAAATATCAAGGACAAAAAGAAAAGAAGCGAAGAGAAAATCAAACTTGAGGAAATCGAAAATACTTACAAAAATAAGTTCGATGATCTGAGAAACACTTTGATTGACAAATTGAATACCTTGGTTTCTGGTAAAACTTCTCAGGGTGTGAAAAATGACCTTGAAGAGGAAGTAATCAGCAAAGGAACCAAATTCACTTTGAAATTGCTTCAGTCTGTAGAAGATTATGTGAACATCAGCGGTGCAGATTGGACCGTAGATGCAGACAAAAATGAATGGATCAAACAATTGATTCACAATTATAAAATCAAATACAATGATCTTTCGGGAGTTAAAAACCGTGAGAAATTTGCATTGTCTATCGGAGACGAGTTACCAGCAGGTATTATCAAATTAGCTAAAGTTTACATCGCTAAGAAACGTAAACTGAATGTAGGTGATAAAATGGCAGGTCGTCACGGTAACAAAGGTATCGTTTCAAGAATCGTTCGTGAAGAAGATATGCCATTCTTGGAGGATGGAACACCAGTAGATATCGTATTGAATCCGCTTGGGGTACCTTCCCGTATGAATATTGGTCAGATCTACGAAACTGTTCTTGGATGGGCAGGTACAAAACTGGGATTGAAGTTTGCAACACCGATCTTTGATGGTGCAAGTCTTGAGCAAATCACAGAATATACTGATCAGGCAGGTCTTCCGAAATTTGGTAGCACACACTTATACGACGGTGGAACTGGGGAGAGATTTACGCAGCCTGCAACTGTAGGTGTGATCTACATGTTGAAACTTGGACACATGGTAGATGACAAAATGCACGCACGTTCTATCGGACCTTACTCATTGATCACGCAACAGCCGTTAGGTGGTAAAGCACAATTCGGAGGTCAGAGATTCGGAGAGATGGAGGTTTGGGCTCTTGAAGCATTTGGAGCGTCTAACATCCTGAGAGAGATCTTGACTGTGAAGTCGGATGACGTGATTGGTAGAGCAAAAACTTATGAAGCGATTGCAAAAGGAGAAGCAATGCCAGAACCTGGTATTCCGGAATCTTTCAACGTATTACTTCACGAGTTACAAGGTCTTGGACTTGATGTAAGAATCTTACTTCTTGTGTTATGTTTCAATCTTTTGATTGCTTTTTCTTTGATCTGGCGAACTCTTTCTCTCGTAAGGTCAAAAGTTTCACCGATCTCTTCCAAAGTCATCGGGTGTTTTCCGTTCAATCCGAAATATAATCTTACAAGATCAGCTTCTCTTGGTGTCAAAGTTTGAAGTGCTCTTTCTATCTCGATCTGTAGAGATTCCAGCATTAGGTCTTTATCAGGACTTGGAGATTCACCGGAACGCAAAACGTCATATAGGTTAGAATCTTCACCTTCCACAAGTGGCGCATCCATAGATAAGTGACGACCGCTGTTTTTCATAGATTCCTTGATGTCCTCCTCGCTCATATCCAGAACTTCTGCCAGCTCTTCCGGAGAAGGCGGCCTTTCGTTCTCTTGCTCCAGGTGAGCGTAAGCCTTGTTGATCTTGTTGATAGAACCAATCTTATTCAAAGGTAATCTTACAATTCTCGACTGTTCAGCCAGTGCTTGCAAGATCGACTGACGAATCCACCAAACAGCATAAGAGATGAATTTGAAACCTCTCGTTTCATCATATCTTTTTGCCGCTTTCATCAGACCAAGATTTCCTTCGTTGATGAGATCCGGAAGAGAAAGACCTTGATTCTGATACTGTTTAGAAACCGAAACCACGAAACGTAAGTTAGCTTTGATCAGTTTTTCAAGAGCAGCTCTGTCACCGGCACGGATTCTCTGGGCCAGATCTACCTCTTCGTCAGCGGTGATCAATTCAACTTTTCCGATCTCTTGCAGATACTTGTCTAGTGATGCGGTTTCCCTGTTGGTAACCTGTTTTGTAATCTTTAATTGTCTCAT

>Chryseobacterium_indologenes_NBRC 14944T

AGTATTCAGGCATTGGAAGGAATGGAACACGTTCGTATGCGTCCTTCAATGTACATTGGTGATGTAGGGACAAGAGGTCTCCATCATTTGGTTTATGAAGTAGTAGATAACTCTATTGACGAAGCGTTGGCAGGATACTGTGATACGATCTTTGTTGCAATTAAAGAAAGAAACGGAATTGAAGTAAGCGATAACGGTAGAGGTATTCCTGTTGATTTCCACGAAAAAGAACAAAAATCAGCTCTTGAAGTTGTAATGACCAAAATCGGAGCCGGAGGAAAGTTTGATAAAGATTCCTACAAGGTTTCGGGAGGTCTTCACGGAGTAGGGGTATCGTGTGTGAATGCACTTTCCAACGAAATGATCACTACCGTTTACAGAGACGGAAATGTATATCAGCAGGTATATTCCAAAGGAAAAGCACAAACCGAGGTTGAAGAAATAGGGCACAGTGACAGAA-GAGGAACAAAGCAGTTTTTCCAGCCTGATGATACGATTTTCACAGAA---CTGGTTTATAACTATGATACTTTAGCCAGCCGTTTAAGAGAACTTTCCTACCTTAACAAAGGAATCACCATTACTCTTACGGATGAAAGAGAAAAATTGGAAGACGGTTCTTTCCGATCAGAGGTTTTTCATTCTGAAGGCGGTTTAAAAGAGTTTGTTGCCTATATCGATGGAAACAGAGAATCAATCATGGAGAATGTTATCTTCATGGAAGGCGAAAGAGATGATATTCCGGTGGAAGTAGCGATGCGTTACAATACCTCTTTCAATGAGAACCTGCATTCTTATGTGAACAACATCAATACTCATGAAGGGGGAACCCACCTGGCAGGTTTCAGACGTGCTTTAACGAGAACCCTTAAAAAATATGCTGATGATCTGGGAATTCCGCAGAAAGAAAAAGTAGACATTACCGGAGATGACTTCCGTGAAGGTTTAACAGCAGTAGTTTCTGTAAAAGTAATGGAGCCTCAGTTTGAAGGACAGACTAAAACTAAGCTTGGAAACTCTGAAGTTTCAGGTGCCGTAGATAAGATTGTTGGAGAAATGTTAACCAACTTTCTTGAAGAAAACCCTAATGAAGCAAAACAAATTGTACAGAAAGTGGTTTTGGCAGCAAAAGCAAGACAGGCTGCGAAAAAGGCCCGTGAAATGGTTCAGAGAAAATCCCCGATGGGAGGTTCAGGGCTTCCTGGAAAGCTTTCTGACTGTTCTTCCAAAGATCCGGCTGAATCTGAATTATTCCTGGTAGAGGGAGATTCCGCAGGTGGAACCGCAAAACAGGGAAGAGACAGACATTTTCAGGCAATCCTGCCTTTAAGAGGTAAGATCCTGAATGTGGAAAAATCTATGCTTCATAAAGTATATGATAACGAAGAAATCAAAAATATTTATACTGCGCTTGGAGTTTCTGTAGGAACAGAAGAAGACAGCAAAGCGTTGAATATGACTAAATTGAGATACCACAAAATTGTCATCATGACCGATGCTGATATTGATGGATCTCACATTTCTACCCTGATTCTTACATTCTTCTTCAGATATATGAAAGAGCTTATTGAAAATGGATATATTTACATTGCCCAGCCGCCTCTATATCTATTGAAGAAAGGAAATAAGAAAGTATATGCTTATAACGAAAAAGAGCGTGAGGAATTTACTCTGGAAATGTCTCCGGATGGAAAAGGAGTAGAAGTACAGCGTTATAAAGGTCTTGGAGAAATGAACCCGGAACAGCTTTGGGAAACAACGCTGAATCCTGAGCATAGAATTTTAAAGCAGGTAACCATTGATAATGCAGTAGAAGCAGACAGTATCTTCTCTATGCTGATGGGGGATGAAGTTCCACCAAGAAGAGAGTTTATTGAGAAAAATGCAAAATATGCTAAGATTGATGAATTCTCCATCATCCAGAATACATTACAAAAAGACCCTACCAACTCTGAAAAAGAAGCGGTAGAGTACATCTATCGTCAGTTAAGAAATGCAGATCCGCCAGATGAGGAAACGGCAAGAGGAATCATTGAAAAATTATTCTTCTCCGAGCAGAGATACTCTTTAGGTGAAGTAGGACGTTACAGACTGAACAAAAAGCTTGGGCTTAACATCCCTACTACAACTGAAGTTCTTACCAAAGAAGATATCATTGCAATCGTAAGACACTTAATCGAACTTGTTAACTCTAAAGCAGAGGTTGATGATATTGACCACCTTTCTAACAGAAGAATCAAAACTGTTGGAGAGCAGCTGGCAGGGCAGTTCGGAGTAGGTCTTTCAAGAATTGCAAGAACAATCAAGGAAAGAATGAACGTTAGAGATAACGAAATCTTTACTCCGCTTGATCTTGTAAATGCGAAGACATTAACATCCGTAATCAACTCGTTCTTCGGTACCAACCAGCTGTCTCAGTTCATGGACCAGACCAACCCGCTGTCAGAGATCACTCACAAGAGAAGATTGTCTGCCCTAGGGCCTGGTGGTTTATCAAGAGAAAGAGCAGGTTTCGAGGTTCGTGACGTTCACCATACCCACTACGGAAGAATCTGTCCGATTGAAACTCCGGAAGGACCAAACATCGGTTTGATTTCCTCTTTAGGAATCTATGCAAAAATCAACAGACTTGGTTTCATTGAAACTCCATACAGAAAAGTAGAAGACAGCAAGATTGATCTTAATGCAGACCCTATTTATCTTAATGCAGAAGACGAAGAAGATAAAGTAATTGCTCAGGCAAACGTTGAATTGAGTGATAACGGAGACTTCTTAACAGACAGAATTATTGCAAGACTGGATGGTGATTACCCTGTAGTGGAGCCTTCTCAGGTTAACCTTATCGATGTTGCACCAAACCAGATCTCCGGTATTTCCGCTTCATTAATTCCATTCCTGGAGCATGATGATGCGAACCGTGCATTGATGGGATCCAACATGATGCGTCAGGCCGTTCCTTTGTTGAAGCCACAGGCTCCAATCGTAGGTACAGGTCTGGAACAACAGGTTGCCAAAGATTCAAGAATTCTGATCAATGCTGAGGGTACAGGTACTGTAGAATATGTAGATGCTGATAAGATCACTATTAAATATGAAAGAAGCGAGGACGAAGATTTAGTACAATTCGAGTCTGCTACTAAAACATATAACCTTACCAAGTTCAGAAAAACCAACCAGAGTACAACTATTACGCTAAGACCAAACGTAAGAGTAGGTGATGTAGTGGAAAAAGGACAGGTTCTTTGCGACGGTTACGCTACTGAAAACGGAGAATTGGCTCTTGGTAGAAACCTGGTAGTTGCGTTCATGCCTTGGAAAGGATACAACTTTGAGGATGCAATCGTAATCAACGAAAAAGTTGTACGTGAAGACTGGTTTACTTCAATCCACGTAGACGAATACTCTCTTGAAGTTCGTGATACCAAATTAGGTATGGAAGAGCTTACAGCAGATATTCCAAACGTATCTGAAGAAGCTACCAAAGATCTTGACGAGAACGGTATGATCAGAATCGGTGCAGAAGTGAAGCCTGGAGATATCATGATTGGTAAAATCACTCCAAAAGGTGAATCTGACCCAACACCTGAAGAAAAACTTCTTAGAGCAATCTTCGGTGATAAAGCTGGAGATGTGAAAGATGCTTCATTGAAAGCTGACTCTTCATTAAGAGGTGTGGTGATCAATAAGAAACTGTTCTCAAGAAACATTAAAGACAAAAAGAAAAGAACCGAAGAAAAACTTAAACTTGAAGAAATTGAAAACACTTACAAGGCTAAGTTTGACGAGCTGAGAAACACTTTAATTGAAAAACTAAACACGCTGGTAAGCGGTAAAACTTCTCAGGGAGTGAAAAACGACCTTGATGAGGAAATTATCGGTAAAGGAGTGAAGTTTACTCACAAACTCTTAACTTCAGTTGAAGATTATGTAAACGTTAGCGGTTCAGACTGGACAGTTGATGCTGATAAGAATGAATTGATCAAGCAATTGATTCACAACTACAAAATCAAATACAACGATATTCAAGGAGTTAAAAACCGTGAGAAATTTGCTATTTCAATCGGTGATGAACTTCCTGCAGGTATCATGAAGCTGGCTAAAGTTTACATTGCTAAGAAACGTAAACTGAATGTAGGAGATAAAATGGCGGGACGTCACGGTAACAAAGGTATCGTTTCAAGAATCGTTCGTGAAGAAGATATGCCGTTCCTTGAAGATGGAACACCGGTAGATATCGTATTGAACCCGCTAGGGGTACCTTCACGTATGAACATTGGTCAGATTTATGAGACAGTTCTTGGATGGGCTGGTCAGAAATTAGGATTGAAGTTTGCTACCCCAATCTTTGACGGAGCAAGTCTTGAGCAGATTACAGAGTATACAGATAAAGCAGGTCTTCCTAAATTCGGTCACACTCACCTTTATGATGGGGGTACCGGAGAAAGATTTACTCAGGCAGCTACAGTAGGTATTATCTACATGCTGAAACTGGGGCACATGGTTGACGATAAGATGCACGCACGTTCTATTGGACCTTACTCATTAATCACGCAGCAGCCGTTAGGAGGTAAAGCTCAGTTCGGAGGGCAGAGATTCGGAGAGATGGAGGTTTGGGCTCTTGAAGCATTCGGAGCATCCAACATCCTTAGAGAAATCCTTACCGTGAAATCGGATGACGTGATTGGTAGAGCGAAAACTTACGAAGCAATTGCAAAAGGTGAATCTATGCCTGAACCAGGTATTCCGGAATCATTCAACGTATTGCTTCACGAGTTACAAGGTCTTGGATTAGACGTAAGAATCTTACTTCTGGTGTTGTGTTTCAATCTCTTGATCGCTTTTTCTTTGATCTGACGTACTCTCTCTCTTGTAAGGTCGAAAGTTTCACCAATTTCTTCTAAAGTCATTGGGTGTTTCCCGTTCAGTCCGAAGTATAATCTTACCAAATCAGCCTCTCTTGGAGTCAGAGTATTCAATGCTCTCTCAATCTCGATTTGCAGAGACTCAAGCATCAGATCTTTATCCGGGCTTGGTGATTCTCCTGAACGCAATACATCATAAAGGTTAGAATCTTCACCTTCTACTAAAGGTGCATCCATAGACAGGTGTCTTCCGGAGTTTTTCATAGATTCTTTGATATCCTCCTCGCTCATGTCAAGAACTTCGGCCAATTCTTCCGGAGAAGGTGGTCTTTCGTTTTCCTGTTCAAGGTGAGCGTATGCTTTATTAATTTTATTGATGGAACCAATTTTGTTCAACGGAAGTCTTACAATTCTTGATTGCTCAGCCAACGCCTGCAAAATTGATTGACGGATCCACCATACTGCATAAGAGATAAATTTGAAACCTCTAGTTTCATCATACCTTTTTGCCGCTTTCATCAATCCTAAGTTACCTTCATTAATCAAATCGGGTAAAGAAAGGCCTTGATTCTGGTATTGTTTAGATACGGAAACTACGAAACGAAGGTTGGCTTTGATTAATTTCTCAAGTGCAGCTCTGTCGCCAGCACGTATTCTTTGTGCCAATTCTACTTCTTCGTCCGCAGTGATCAGTTCTACTTTACCAATTTCCTGCAAGTACTTGTCTAATGAAGCAGTTTCCCTATTGGTAACCTGCTTAGTGATCTTTAATTGTCTCAT

>Chryseobacterium_jeonii_NCTC13459T

AGCATCCAAGCTTTAGAAGGAATGGAACACGTAAGAATGAGACCTTCAATGTACATCGGTGATGTAGGTTTAAGAGGTTTACATCATTTAGTTTACGAAGTTGTTGACAACTCCATTGATGAGGCTTTAGCGGGACATTGCGACACCATATCAGTAATCATTCATGAAGGTGAAAGTATCTCTGTAAAAGATAACGGTCGTGGAATTCCAGTAGATTTACACGAGAAAGAACAGAAGTCAGCTTTGGAAGTTGTAATGACCAAAATTGGAGCTGGTGGAAAATTCGATAAAGATTCTTACAAAGTTTCCGGTGGTCTTCACGGAGTTGGTGTTTCGGTGGTAAATGCACTTTCAAATTCCTTAATTGCAACCGTTAAGAGAGATAGCAAAGTTTATGAGCAGAAATATTCTAAAGGAAAAGCCTTAGCAGATGTTACGGAAATTGGAATTACTACTGAAA-GAGGAACCGAAGTTTTCTTTCAACCTGACGATACTATTTTTCAGGAA---TTAGTCTTTAATTACGATACTTTAGCAAGTAGAATGCGAGAACTTTCTTATCTAAATAAAGGGATAAAAATTACCTTAACAGATGAACGCGTAACAGAAGAAAATGGTGAATTTAAAAGCGAACTGTTTTATTCTGAAGGTGGTCTGAGAGAATTCGTAGAATACATCGACGGTAATCGTGAAAGTATTATGAATAGTGTCATTTTCATGGAAGGTGAAAAAGATAATATTCCTGTAGAAGTTGCGATGAGATACAACACTTCTTACACCGAAAATCTGCACTCTTACGTTAATAATATCAATACTCATGAAGGTGGAACTCACCTCGCAGGTTTCAGAAGAGCTTTAACGAGAACTTTGAAGAAATTTGCAGATGAATTAGGACTTCCTGCAAAAGAAAAAGTAGAAGTTACCGGTGACGATTTCCGTGAAGGTTTAACTGCCGTGATTTCTGTAAAGGTAATGGAGCCTCAGTTTGAAGGTCAAACCAAAACGAAATTAGGAAACTCTGAAGTTTCTGGTGCAGTTGATAAGATCGTGGGCGAAATGTTGAGCAACTTTTTGGAGGAAAATCCAAATGAAGCAAAATTGATCGTTCAGAAAGTAGTTTTAGCTGCAAAAGCGAGACAAGCTGCAAAAAAAGCAAGAGAACTCGTTCAACGTAAATCGCCAATGGGAGGAAGTGGATTACCAGGAAAACTTTCTGATTGTTCTTCCAAAGATCCTGCAATTTCTGAGTTATTCTTAGTCGAGGGAGATTCTGCGGGTGGAACAGCAAAACAAGGTCGTGACCGCCATTTTCAAGCGATTCTACCATTAAGAGGTAAAATTTTGAACGTAGAGAAAGCAATGGTTCACAAAGTTTACGACAACGAAGAGATCAAAAATATTTATACCGCACTTGGTGTTTCTGTAGGAACTGAAGAAGATTCGAAAGCCCTAAATATTGCAAAATTAAGATACCATAAAGTAGTTATCATGTGTGATGCCGATATTGATGGTGCTCACATTTCAACTTTAATTATGACTTTCTTCTTCAGATATATGAAGGAATTAATTGAAAATGGATATATTTATATTGCACAACCTCCTTTATATTTATTAAAAAAAGGAAGTAAAAAGATTTATGCCTACAACGAAAAAGAACGTGAACAAATAACGTTAGAAATGGCTCCAGACGGGAAAGGCGTTGAAGTACAACGTTACAAAGGTCTTGGGGAAATGAATCCAGAACAACTTTGGGATACTACATTGAATCCTGAAAATAGAATTTTAAAACAAGTTACGATCGAAAGTTTAGCAGAAGCTGACAACGTATTCTCTATGTTGATGGGCGATGAAGTACCACCAAGAAGAGAATTCATCGAGAAAAATGCTATCTATGCAAAAATTGATGAATTCTCTATTATTCAGAATACTTTACAAAAAGATCCAACCAACTCTGAAAAAGAAGCGGTTGAATATATTTATCGTCAGTTGCGTAATGCAGATCCACCCGATGAAGAAACAGCAAGAGGAATTATCGAAAAATTATTCTTCTCTGAGCAACGTTATTCACTAGGAGAAGTTGGTCGTTACAGATTGAACAAAAAATTAGGATTAAATATTCCTGCGAAAACTGAAGTTTTAACCAAAGAAGATATCATCTCGATCGTAAGACATTTGATCGAATTGGTAAACGCTAAAGCGGAAGTTGATGATATTGATCACTTGTCAAACAGACGTATTAAAACTGTTGGTGAGCAATTATCAGGACAGTTTGGAGTAGGTCTTTCCAGAATTGCAAGAACAATCCGTGAGAGAATGAACGTTAGAGATAACGAAATCTTTACTCCGATTGACTTGGTAAACGCAAAAACTTTAACCTCTGTAATTAATTCATTCTTCGGAACGAATCAATTATCTCAGTTCATGGATCAAACCAATCCATTATCAGAAATCACGCACAAGCGTAGACTTTCTGCTCTAGGACCTGGTGGACTTTCAAGAGAAAGAGCAGGTTTTGAGGTACGTGACGTTCACCATACGCACTACGGAAGAATTTGCCCGATAGAAACTCCAGAGGGACCAAACATTGGTTTGATTTCTTCTCTAGGAATTTATGCAAAAATTAATACTTTAGGTTTTATCGAAACGCCTTATAGAAAAGTAAACAACGGTAAAGTTGATCTTAAAACTGCTGCTATTTTCTTGAATGCTGAAGATGAAGAAGATAAAGTAATTGCACAGGCAAACGTTGAGATGCTTGATGATGGTACTATTTCTACAGATAGAGTAATTGCTCGTTTAGACGGTGATTATCCAGTAGTTGAACCACAACAGGTTGACTTGATTGATGTTGCACCAAATCAGATTTCTGGTATTTCTGCTTCATTGATTCCTTTCCTGGAACATGATGATGCGAATAGAGCGTTGATGGGATCGAATATGATGCGTCAAGCAGTTCCATTGTTGAAACCAGAAGCTCCGATTGTTGGAACAGGTTTAGAAAAACAAGTTGCGAGTGACTCCAGAATTTTAATTAATGCTGAAGGAAACGGAACAGTAGAATACGTTGATGCGGTAAAAATTACCATTAAATATGAAAGAAGTGAAGATGCTGATTTAGTATCATTCGATACCGCTACGAAATCATATAAATTAACCAAATTCCGTAAGACCAATCAGGGAACGACCATTACTTTAAGACCAAACGTAAGAGTAGGTGATAAAGTAGTAAAAGGACAGGTTCTTTGTGATGGTTATGCAACTGAAAACGGAGAATTGGCACTTGGACGTAACTTAGTTGTTGCCTTCATGCCTTGGAAAGGGTACAACTTTGAGGATGCGATCGTTATTAACGAAAAAGTAGTTCGCGAAGACTGGTTTACTTCGATCCATGTTGATGAATATTCTCTGGAAGTTCGGGATACCAAATTAGGTATGGAAGAATTGACAGCAGATATTCCAAACGTTTCAGAAGAAGCAACCAAAGATTTAGATGAAAACGGAATGATCAGAATTGGAGCCGACGTAAAACCTGGTGACATTATGATTGGTAAGATTACTCCAAAAGGAGAATCAGATCCGACTCCGGAAGAGAAATTATTGAGAGCGATTTTTGGAGACAAAGCCGGAGACGTAAAAGATGCTTCATTGAAAGCAGATTCATCGTTGAGAGGAGTTGTAATCAATAAAAAATTATTCTCAAGAAATATCAAAGATAAAAAGAAAAGATCGGAAGAGAAATTGAAATTAGAAGAAATCGAAAATACCTACAAAGGGAAAAATGATGACCTTCGAAATCTGCTTCTTGAAAAATTAGGAACACTTGTAAACGGTAAAACGTCTCAGGGTGTAAATAATGATCTTGATGAAGAAATCATCGGAAAAGGCGTGAAATTTACTACAAAATTACTTCAATCTGTTGAAGATTATGTGAATGTAAGTGGAGCAGACTGGACGGTTGATGCTGAAAGAAATGAGTTGATCAAGCAATTGATCCACAACTTCAAAATTAAGTCTAATGACTTAAATGGTGTTAAAAACCGTGAGAAGTTTGCTATCTCTATCGGAGATGAATTACCTGCAGGAATCATCAAATTAGCGAAAGTATATGTTGCTAAAAAACGTAAACTGAACGTTGGTGATAAAATGGCGGGTCGTCACGGTAACAAAGGTATTGTATCAAGAATTGTACGTGAAGAAGATATGCCGTTCCTTGAAGATGGAACTCCGGTTGATATCGTATTGAATCCACTGGGTGTACCTTCTCGTATGAACATTGGTCAGATCTACGAAACCGTTTTAGGATGGGCTGGTAAAAAATTAGGTTTAAAATTCGCAACGCCGATTTTCGATGGTGCTAATATTGACCAAATTACAGAATATACCAAGCAAGCAAATCTTCCGGAATTTGGTAGTACTTATCTTTATGATGGTGGTACTGGTGAAAGATTCTCTCAACCTGCAACCGTTGGAATCATTTACATGTTGAAATTAGGACACATGGTTGATGATAAAATGCACGCACGTTCTATCGGACCTTATTCATTGATTACGCAGCAACCTTTGGGTGGTAAAGCGCAGTTTGGTGGACAAAGATTTGGTGAGATGGAGGTTTGGGCGTTGGAAGCATTTGGTGCTTCGAATATCCTTAGAGAAATCTTAACTGTGAAATCCGATGACGTGATTGGTAGAGCTAAAACTTACGAAGCAATTGCAAAAGGAGAAGCAATGCCTGAACCAGGTATTCCGGAATCCTTCAATGTATTGTTACATGAGTTGCAAGGACTTGGTCTTGATGTAAGAATCTTACTTCTTGTATTATGTTTCAATCTTTTGATCGCTTTTTCTTTGATCTGACGAACTCTTTCTCTTGTTAGATCAAAAGTTTCACCGATTTCTTCTAAAGTCATTGGATGTTTTCCGTTCAAACCGAAATACAAACGAACCAAATCAGCCTCACGCGGAGTCAGAGTTTGCAATGCTCTTTCGATCTCAATCTGAAGAGATTCCAACATCAAATCCTTATCCGGACTTGGAGATTCACCTGAACGCAAAACGTCGTATAAATTCGAATCTTCACCTTCAACCAATGGCGCATCCATCGACAAGTGTCTTCCGGAGTTTTTCATTGATTCCTTAATGTCATCTTCACTCATATCCAAAACTTCCGCTAACTCTTCTGGGGACGGAGGTCTTTCGTTTTCCTGCTCCAAATGTGCGTAAGCTTTGTTAATTTTATTGATCGATCCAATTTTATTTAATGGCAATCTTACAATTCTCGACTGTTCTGCCAAAGCCTGTAGAATCGACTGACGAATCCACCAAACGGCGTAGGAGATAAATTTAAAACCCCTTGTTTCATCATATCTTTTCGCAGCTTTCATCAAACCAAGATTTCCTTCGTTAATTAAATCGGGCAAAGAAAGACCTTGATTCTGGTATTGTTTAGATACAGAAACCACGAAACGCAAGTTGGCTTTAATCAATTTTTCCAGGGCAACACGGTCTCCAGCACGAATTTTTTGCGCCAAGTCAACTTCTTCGTCGGCGGTAATCAAATCTACTTTTCCGATTTCCTGTAAATACTTGTCCAGCGATGCGGTTTCCCTGTTGGTAACCTGCTTTGTAATTTTTAATTGTCTCAT

>Chryseobacterium_koreense_CCUG 49689T

AGTATTCAGGCGCTTGAAGGAATCGAACACGTAAGGTTAAGACCATCGATGTACATCGGTGATGTGGGAACCAGAGGTCTTCACCATTTGGTTTATGAAGTAGTGGATAATTCGATTGACGAGGCGCTCGCTGGACATTGCGACACCATCGATGTAAGAATCCATGAGGGAGACGGAATTTCCGTGCGTGACAACGGCCGAGGAATTCCGGTAGATTTTCACGAAAAAGAGCAGAAATCCGCATTGGAAGTGGTCATGACCAAAATCGGTGCCGGAGGAAAATTCGACAAAGATTCCTACAAAGTTTCCGGTGGTCTCCACGGTGTAGGGGTTTCCTGCGTGAATGCGCTTTCAACCTCACTGATTGCAACCGTTTACCGTGACGGAAAAGTCTATCAGCAAAAATATTCCAAAGGTCACGCTTTAGCAGATGTGGAAGTCATCGGCGAAGCAGACGCAA-GAGGAACCAGCGTTTTCTTTCAACCCGATCCAAGTATTTTCCAGGAA---CTGGTCTATAATTATGATACTTTGGCAGCACGTCTCCGTGAACTGGCTTTCCTGAACAAAGGAATCACCATCACTTTGACAGACGAAAGAGTTAAAGATGAACAAGGTGAACCAAAAACAGAAGTATTCCATTCCGAAGGCGGGTTGAAAGAATTCGTGCAATACATCGACGGAAACCGCGAAAGCATTATGCAGAACGTGATCTTCATGGAGGGAAACAAGGACGACATTCCAGTGGAAGTGGCAATGCGCTACAACACCTCCTACAATGAAAACCTGCATTCTTATGTAAACAACATCAATACCCATGAAGGCGGAACGCACCTTGCAGGTTTCAGACGTGCTTTGACGAGAACTTTGAAGAAATTTGCAGACGAACTCGGTCTTCCAGCAAAAGAAAAAGTAGAAGTAACCGGTGACGATTTCCGGGAAGGGTTGACCGCAGTGATTTCCGTGAAAGTGATGGAACCACAGTTTGAAGGCCAAACCAAGACCAAGTTAGGAAACTCCGAAGTTTCTGGTGCGGTGGACAAAATCGTGGGCGAAATGCTCACCAACTTCCTGGAGGAGCATCCGAACGAAGCGAAACTCATCGTGCAGAAAGTGGTTCTAGCAGCAAAAGCAAGACAAGCCGCGAAAAAAGCGAGAGAACTTGTACAAAGAAAATCCCCGATGGGCGGAAGTGGACTTCCTGGGAAACTTTCGGACTGTTCCTCAAAAGACCCTGTAATCTCAGAAATATTCTTGGTAGAGGGAGATTCCGCAGGTGGAACCGCAAAGCAAGGTCGAGACAGACATTTTCAGGCGATTCTTCCACTGCGAGGTAAGATTTTGAATGTGGAAAAATCTATGCTCCACAAAGTTTACGATAACGAGGAAATCAAAAATATTTACACCGCGTTAGGAGTTTCCGTAGGAACTGAAGATGATTCCAAAGCTTTGAATCTAGCCAAACTCAGATACCACAAAATCGTGATCATGACCGATGCGGATATTGATGGATCCCACATTTCTACACTGATTTTGACTTTCTTCTTCCGCTACATGAAGGAGCTGATCGAAAACGGATATATCTATATTGCGCAACCACCGTTGTATCTTTTGAAAAAAGGAAACAAGAAAATATACGCCTATAACGAAAAAGAACGCGAAGAAAAAACTTTGGAAATGTCTTCCGATGGAAAAGGCGTTGAAGTTCAGCGATACAAAGGTCTCGGTGAAATGAACCCGGAACAGCTTTGGGAAACCACGCTGAATCCGGAACACCGAATTCTGAAGCAAGTCACCATCGACAATTTGGTGGAAGCAGACAGCACCTTCTCCATGCTTATGGGAGACGAAGTGCCGCCAAGAAGAGATTTCATAGAGAAAAACGCGATCTATGCCAAAATTGACGAGTTCTCCATCATCCAAAATACTTTGCAAAAAGACCCTACCAACTCTGAAAAAGAAGCGGTAGAATATATTTACCGTCAGCTGAGAAACGCTGATCCGCCAGATGAGGAAACTGCAAGAGGAATCATCGAGAAATTATTCTTCTCCGAGCAGCGATATTCATTAGGTGAAGTAGGCCGTTACAGACTGAACAAAAAATTAGGATTAAATATTCCTGAAAAAACGGAAGTTTTAACTAAAGAAGATATCATCTCCATCGTTCGCCACTTAATCGAATTGGTGAATTCCAAAGCGGAAGTGGATGATATTGACCACTTGTCCAACAGAAGAATCAAAACTGTTGGTGAGCAATTGGCGGGTCAGTTCGGTGTTGGTCTTTCTAGAATTGCCAGAACAATCCGTGAAAGAATGAACGTTAGAGATAACGAAATCTTTACGCCTATCGATTTGGTTAATGCCAAAACTTTGACCTCTGTGATCAATTCGTTCTTCGGAACCAACCAGTTGTCCCAGTTCATGGACCAAACCAACCCACTTTCGGAAATCACTCACAAGAGAAGACTTTCCGCATTAGGACCTGGTGGACTTTCCAGAGAGAGAGCAGGTTTCGAGGTTCGTGACGTTCACCATACGCACTACGGAAGAATTTGCCCAATTGAAACTCCGGAAGGACCAAACATCGGTTTGATTTCTTCACTAGGTATCTATGCGAAAATCAACAACTTAGGATTTATTGAAACTCCATACCGAAAAGTAAAAGACGGAAAAGTTGACTTGAAGAGTGCGCCGATTTTCCTTAATGCAGAAGATGAAGAGAATAAAGTAATCGCACAGGCCAACGTGGAAATGGAAGATGACGGAACCATCTCTACAGATAGGGTTATTGCCCGACTTGATGGAGATTATCCGGTGGTGGAACCTTCAGAAGTGAACTTGATCGATGTGGCGCCAAACCAGATTTCCGGTATTTCCGCTTCATTGATTACTTTCTTGGAACATGATGATGCGAACCGTGCATTGATGGGATCCAACATGATGCGTCAAGCAGTTCCGCTATTGAAGCCGCAAGCTCCGATTGTGGGAACAGGTCTTGAAAAACAGGTCGCAAAAGATTCCAGAATATTGATCAATGCTGAAGGAAATGGAGTGGTGGAATATGTGGATGCAGATACGATCACCATTAAATATGAAAGAAGTGAAGACGAAGATTTGGTTTCGTTCGAATCCGCTACGAAATCCTATAAGTTGACCAAATTCAGAAAAACCAACCAATCCACTACGATTACTTTGAGACCGAACGTAAGAGTTGGTGACAAGGTGACCAAAGGACAGGTTCTTTGTGATGGATATGCAACCGAAAACGGAGAATTGGCATTGGGTAGAAACTGCGTGGTAGCGTTCATGCCTTGGAAAGGTTACAACTTCGAGGATGCAATCGTGATCAACGAAAAAGTAGTTCGTGAGGACTGGTTTACTTCAATCCACGTGGATGAATATTCTCTGGAAGTTCGTGATACCAAATTGGGTATGGAAGAATTGACCTCAGATATTCCAAACGTTTCTGAGGAAGCAACCAAAGATCTGGACGAAAACGGAATGATCAGAATTGGTGCGGAAGTGAAACCTGGCGACATCATGATTGGTAAAATCACGCCAAAAGGGGAATCCGATCCAACTCCTGAAGAAAAACTATTGAGAGCGATTTTCGGTGACAAAGCCGGTGATGTGAAAGATGCTTCCCTGAAAGCCGATTCATCATTGCGAGGTGTTGTGATCAACAAAAAATTGTTCTCCAGAAATATCAAAGACAAAAAGAAAAGAACCGAGGAAAAACTGAAGCTTGAAGAAATCGAAAATACCTACAAAGCAAAATTCGATGACTTAAGAAACACCCTCCTCGAAAAACTCGGAACTCTGGTAAACGGTAAAACTTCTCAAGGAGTAAATAACGACCTTAATGAAGAAATCATCGGAAAAGGAGTGAAGTTCACCACCAAATTGCTTCAGAGTGTTGAAGATTACGTCAACGTAAGTGGAGCAGATTGGACCGTTGATGCAGATAAAAACGAGTTGATCAAGCAATTGATCCACAACTACAAGATCAAGTACAATGACATCATGGGAGTGAAAAACCGTGAGAAATTTGCAATTTCAATCGGGGACGAACTTCCTGCAGGAATCATTAAATTGGCCAAAGTATATATCGCTAAGAAACGTAAGTTGAATGTTGGTGATAAAATGGCGGGTCGTCACGGAAACAAGGGTATCGTATCCAGAATCGTTCGTGAGGAAGACATGCCATTCTTGGAAGACGGGACTCCGGTTGACATCGTGTTGAACCCACTTGGTGTACCTTCACGTATGAACATCGGCCAGATTTACGAAACCGTTCTTGGCTGGGCCGGTCAGAAATTAGGATTGAAATTCGCAACTCCGATTTTCGATGGTGCTGAATTGGAAGAAATCAACGAGTACACTGACAAAGCAGGACTTCCAAGATACGGAAGCACTTATCTTTATGATGGTGGAACCGGTGAAAGATTCTCACAGCCGGCGACAGTGGGTGTAATCTACATGTTGAAATTAGGCCACATGGTAGATGACAAAATGCACGCACGTTCCATCGGACCTTACTCATTGATTACGCAGCAACCACTCGGTGGTAAAGCGCAATTCGGTGGGCAGCGTTTCGGGGAGATGGAAGTTTGGGCGCTTGAAGCGTTCGGAGCATCAAATATTTTACGCGAAATCTTGACCGTGAAATCTGATGACGTGATTGGTAGAGCAAAAACTTACGAAGCCATCGCAAAAGGAGAGGCTATGCCGGAACCTGGTATTCCAGAATCCTTCAACGTTCTTCTGCACGAACTTCAAGGTTTAGGTTTAGATGTAAGAATCTTGCTTCTGGTATTGTGCTTCAGTCTTTTGATGGCTTTTTCCTTGATTTGCCGAACCCTTTCTCTGGTCAGATCAAATGTTTCACCAATTTCTTCCAAAGTCATCGGGTGTTTTCCATTGAGCCCGAAATAGAGTCGAACCAAATCGGCTTCTCTAGGCGTCAGTGTTTGCAAAGCTCTTTCAATTTCAATCTGAAGAGACTCCAACATCAAATCTTTATCAGGACTTGGCGATTCGCCGGAACGCAAAACGTCATACAAATTGGAATCTTCACCTTCCACTAAAGGGGCATCCATTGACAAGTGACGACCCGAATTTTTCATCGATTCCTTGATGTCGTCCTCACTCATATCAAGTACTTCCGCCAATTCTTCAGGAGAAGGAGGTCTTTCATTTTCCTGCTCTAGGTGCGCGTAAGCTTTGTTGATCTTGTTGATTGAACCAATTTTGTTCAAAGGCAATCTCACAATTCTGGACTGTTCCGCCAAAGCCTGCAAAATAGATTGGCGGATCCACCAAACCGCATAAGAAATGAATTTAAAACCTCTGGTCTCGTCATATCTTTTCGCCGCTTTCATCAAGCCCAGATTTCCTTCGTTAATCAAATCGGGGAGTGACAAACCCTGATTTTGGTATTGTTTAGAAACAGAAACCACGAAACGCAGATTCGCCTTGATCAGTTTTTCCAGTGCAACTCTGTCTCCGGCACGGATTTTTTGTGCGAGTTCCACCTCTTCATCGGCAGTAATCAGATCCACTTTCCCGATTTCCTGCAAATATTTGTCAAGCGACGCGGTTTCTCTGTTGGTAACCTGTTTTGTAATCTTTAATTGTCTCAT

>Chryseobacterium_lactis_NCTC11390T

AGTATTCAGGCATTGGAAGGAATGGAGCACGTACGTATGCGTCCTTCAATGTACATTGGTGATGTGGGAGTAAGAGGTCTCCATCATTTGGTTTATGAAGTAGTAGATAACTCTATTGACGAAGCGTTGGCAGGATACTGCGATACCATTTTCGTCAACATTAAAGAAGGCAACGGAATCGAGGTTAGCGATAACGGTAGAGGTATTCCGGTTGACTTCCACGAAAAGGAACAAAAATCTGCTCTTGAAGTTGTAATGACAAAAATAGGGGCCGGTGGTAAGTTCGATAAAGATTCTTACAAGGTTTCAGGAGGTCTTCACGGAGTAGGGGTATCGTGTGTGAATGCACTTTCCAACGAGATGATCACTACGGTTTACAGAGACGGAAATGTTTATCAGCAGATCTACTCCAAAGGTAAAGCTCAGACTGGTGTTGAAGAAATCGGGCACAGTGACAAAA-GAGGAACCAAGCAGTTTTTCCAGCCGGATGATACTATTTTTACAGAA---TTAGTTTACAATTACGATACATTAGCAAGCCGTTTAAGAGAGCTCTCTTACCTTAATAAAGGGATTACGATTACTCTTACCGATGAAAGAGAGAAATTGGAAGACGGTTCTTTCAGATCAGAAGTTTTTCATTCTGAGGGAGGATTAAAAGAATTCGTTGCCTATATTGATGGAAATCGTGAGTCTATTATGGAGCATGTTATTTTCATGGAAGGAGAAAGGGATAACATTCCGGTAGAAGTAGCAATGCGTTACAACACTTCTTTCAATGAGAATCTCCATTCTTATGTAAATAATATCAATACTCATGAAGGAGGTACTCACCTTGCAGGTTTCAGACGTGCTTTGACGAGAACCCTTAAAAAATATGCTGACGACTTAGGAATTCCGCAGAAAGAAAAAGTGGAAATTACAGGAGATGACTTCCGTGAAGGATTAACAGCCGTAGTTTCTGTAAAGGTAATGGAACCTCAGTTTGAAGGCCAGACAAAAACAAAACTGGGGAACTCTGAAGTTTCTGGGGCTGTAGATAAAATTGTAGGGGAAATGCTTACTAATTTCCTTGAAGAAAATCCTAATGAAGCTAAACAGATTGTTCAGAAAGTAGTATTGGCTGCAAAAGCAAGGCAGGCTGCTAAAAAAGCCCGTGAAATGGTTCAGAGAAAATCTCCGATGGGAGGTTCCGGACTTCCGGGAAAACTATCTGACTGTTCATCCAAAGATCCGGCAGAATCTGAGATCTTCCTGGTAGAGGGGGATTCCGCAGGTGGAACCGCGAAACAAGGGCGTGACAGACACTTCCAGGCCATTCTTCCATTAAGAGGTAAAATCCTGAACGTAGAAAAGTCTATGCTTCACAAAGTATACGATAACGAAGAGATCAGAAATATCTATACTGCTCTTGGAGTTTCTGTAGGAACTGAAGAAGACAGTAAAGCGCTAAACCTTAGTAAGCTGAGATATCATAAAATTGTAATCATGACCGATGCCGATATCGATGGATCTCACATCTCTACACTGATTCTTACGTTCTTCTTCAGATATATGAAAGAACTTATTGAGAACGGATATATCTATATTGCCCAGCCACCTTTATACCTGTTAAAGAGAGGAAATAAAAAGGTATATGCTTATAACGAAAAAGAACGAGAAGAGTTTACCCTGGATATGTCTCCGGATGGAAAAGGAGTAGAAGTACAGCGTTACAAAGGTCTTGGGGAAATGAATCCTGAGCAGCTTTGGGAAACAACCCTGAATCCTGAGCATAGAATTCTAAAACAGGTAACGATTGATAATGCTGTAGAAGCAGACAGTATTTTCTCAATGTTGATGGGAGATGAGGTTCCGCCAAGAAGAGAATTTATCGAGAAAAATGCAAAATATGCAAAAATTGATGAGTTCTCTATCATTCAGAATACATTACAAAAAGACCCTACTAACTCTGAAAAAGAGGCAGTAGAGTACATTTATCGTCAGTTAAGAAACGCAGATCCGCCAGATGAGGAAACTGCAAGAGGAATTATTGAAAAATTATTCTTCTCTGAGCAGAGATACTCTCTTGGTGAAGTAGGACGTTACAGACTAAACAAAAAGTTAGGTCTTAACATCCCGACTACTACTGAGGTTCTTACAAAAGAAGATATCATTGCGATCGTAAGACACTTGATCGAGCTTGTAAACTCTAAAGCGGAAGTGGATGACATCGACCACTTATCCAACAGAAGAATTAAAACTGTTGGTGAGCAATTGGCAGGACAGTTTGGAGTAGGTCTTTCAAGAATTGCAAGAACAATCAAGGAAAGAATGAACGTTAGAGATAACGAAATCTTTACTCCACTTGATCTTGTTAATGCTAAGACGTTAACGTCTGTAATCAACTCATTCTTTGGTACCAACCAGCTTTCTCAGTTTATGGACCAAACCAACCCATTGTCAGAGATTACTCACAAGAGAAGATTATCTGCATTAGGACCTGGTGGTTTATCAAGAGAAAGAGCAGGTTTCGAGGTTCGAGACGTTCACCATACCCACTACGGAAGAATCTGTCCGATTGAAACTCCGGAAGGACCAAACATCGGTTTGATTTCATCTTTAGGTATTTATGCGAAAATCAACAGACTAGGTTTCATCGAAACTCCGTACAGAAAAGTAGAAAATGGTAAAGTAGAGCTTACTGCAGATCCGGTTTACTTAAATGCAGAAGACGAAGAATCTAAAGTAATTGCTCAGGCAAACGTTGAATTGAGCGATAATGGTGATTTCGAAACAGACAGAATTATTGCAAGATTGGATGGTGACTATCCGGTAGTTGAGCCTCAACAGGTTGACCTTATTGATGTTGCACCTAACCAGATTTCCGGTATTTCCGCTTCATTGATTCCGTTCCTGGAGCATGATGATGCGAACCGTGCCCTGATGGGATCCAACATGATGCGTCAGGCCGTTCCTTTATTAAAGCCACAGGCTCCAATCGTAGGTACAGGGCTTGAGCAACAAGTTGCAAGAGATTCAAGAATTTTGATCAACGCTGAAGGTACCGGTACTGTAGAGTATGTAGATGCTGACAAAATCGTTATTAAATATGAAAGAAGCGAAGATGAGGATTTAGTACAATTCGAGTCTGCTACTAAAACATATAACCTTACTAAGTTTAGAAAAACTAACCAGAGTACAACAATTACCCTAAGACCAAACGTAAGAGTAGGTGATGTAGTGGAAAAAGGACAGGTACTTTGTGACGGTTATGCTACTGAAAAAGGAGAATTGGCTCTTGGTAGAAACTTAGTGGTTGCGTTCATGCCTTGGAAAGGATATAACTTCGAGGATGCGATCGTAATCAACGAAAAAGTTGTACGTGAAGACTGGTTTACTTCAATCCACGTAGATGAATATTCTCTTGAAGTTCGTGATACTAAATTAGGTATGGAAGAACTTACAGCAGATATTCCAAACGTATCTGAAGAAGCTACCAAAGATCTTGACGAGAACGGTATGATCAGAATCGGTGCTGAAGTGAAGCCTGGAGATATCATGATCGGTAAGATCACTCCAAAAGGAGAATCTGACCCGACTCCTGAAGAAAAACTTCTGAGAGCTATCTTCGGTGATAAAGCCGGTGATGTGAAGGATGCTTCATTGAAAGCTGACTCTTCATTAAGAGGGGTGGTGATCAACAAGAAGTTGTTCTCAAGAAATATCAAAGACAAAAAGAAAAGAACTGAAGAAAAACTTAAGCTTGAAGAAATCGAAAATACTTACAAAGCTAAGTTTGATGAGTTGAGAAATACTTTAATTGAAAAATTAAATACACTGGTAAGCGGTAAAACTTCTCAGGGGGTACACAACGACCTTGATGAAGAGATCATCGGTAAAGGTGTGAAGTTCACTCACAAGTTATTAACTTCAGTTGAAGATTATGTAAACGTTAGCGGTTCCGATTGGACAGTAGACGCTGATAAGAATGAATTGATCAAACAATTGATTCACAATTACAAAATCAAATATAACGATATCCAAGGGGTTAAAAACCGTGAGAAATTTGCAATTTCAATCGGAGATGAGCTTCCTGCAGGTATTATGAAGTTGGCTAAAGTTTACATCGCTAAGAAACGTAAACTGAATGTAGGGGATAAAATGGCAGGACGTCACGGTAACAAAGGTATCGTATCAAGAATCGTTCGTGAAGAAGATATGCCGTTCCTTGAAGATGGAACACCGGTAGATATCGTATTGAATCCACTTGGGGTACCTTCCCGTATGAACATCGGACAGATTTATGAAACAGTTCTTGGATGGGCTGGTCAGAAGCTGGGAATGAAGTTCGCTACACCAATCTTCGATGGAGCAACGCTTGATCAGATTACAGAATATACAGATAAAGCAGGTCTTCCTAAATTCGGACATACTTACCTTTATGACGGTGGTACCGGAGAAAGATTTACACAGGCAGCTACAGTGGGTGTTATCTATATGCTGAAACTTGGACACATGGTTGATGATAAGATGCACGCACGTTCTATTGGTCCTTACTCATTGATTACTCAACAGCCGTTAGGAGGTAAAGCTCAGTTCGGAGGTCAGAGATTCGGAGAGATGGAGGTTTGGGCACTTGAAGCATTCGGTGCATCCAACATCCTGAGAGAGATCCTGACTGTGAAGTCGGATGACGTGATTGGTAGAGCGAAAACTTATGAAGCGATTGCGAAAGGAGAATCTATGCCTGAACCAGGTATTCCGGAATCATTCAACGTATTACTTCATGAGCTACAAGGTCTTGGATTAGACGTAAGAATCTTACTTCTGGTATTGTGCTTCAATCTCTTGATCGCTTTTTCTTTGATCTGACGCACTCTCTCTCTTGTAAGATCGAAAGTTTCACCAATTTCTTCTAAAGTCATTGGGTGTTTTCCGTTCAGTCCGAAGTATAACCTTACCAAATCTGCCTCTCTTGGAGTCAAAGTATTCAATGCTCTTTCAATCTCGATTTGTAGAGATTCAAGCATCAAATCTTTATCCGGACTTGGTGATTCCCCTGAACGCAATACATCATAAAGGTTAGAATCTTCACCTTCTACTAAAGGTGCATCCATAGACAAATGTCTTCCGGAGTTTTTCATCGATTCTTTAATATCCTCCTCACTCATGTCAAGAACTTCAGCCAATTCTTCCGGAGAAGGTGGTCTTTCATTTTCCTGCTCAAGGTGAGCGTATGCTTTATTAATTTTATTGATGGAACCAATTTTGTTCAACGGAAGTCTTACAATTCTTGATTGCTCAGCCAACGCCTGTAAAATTGATTGACGGATCCACCATACTGCATAAGAGATAAATTTGAAACCTCTAGTTTCATCATACCTTTTTGCCGCTTTCATCAATCCTAAATTTCCTTCATTAATCAAATCGGGTAAAGAAAGGCCTTGATTCTGGTACTGTTTAGATACGGAAACCACGAAACGAAGGTTGGCTTTGATTAGTTTCTCCAGTGCAGCTCTGTCGCCAGCACGTATTCTTTGTGCCAATTCTACTTCTTCGTCCGCAGTAATCAGTTCTACTTTACCAATTTCCTGCAAATACTTGTCTAATGAAGCAGTTTCCCTATTGGTTACCTGCTTAGTGATCTTTAATTGTCTCAT

>Chryseobacterium_molle_DSM 18016T

AGTATTCAGGCATTGGAAGGAATGGAGCACGTTCGTATGCGTCCTTCGATGTACATTGGTGATGTGGGAGTCCGTGGTCTCCACCATTTGGTTTATGAAGTCGTGGATAACTCTATTGACGAAGCTTTGGCCGGATATTGTGATACGATTACTGTTGCAATAAAAGAAGGGAATGCAGTCGAAGTGATGGACAATGGCCGTGGTATTCCTGTCGATTTTCATGAGAAAGAACAGAAGTCTGCTTTGGAGGTTGTAATGACCAAAATCGGAGCCGGAGGTAAGTTTGATAAAGATTCCTATAAAGTTTCCGGAGGTCTTCACGGTGTTGGTGTTTCCTGCGTTAACGCACTTTCCAACGAGATGATCACAACCGTTTACAGAGATGGAAATGTTTATCAGCAGGTTTATTCCAGAGGAAAAGCACAAACTCAGGTTGAAGAAATCGGACACAGTGATAAAA-GAGGAACAAAGCAGTTTTTCCAGCCCGATGATTCTATTTTTACAGAA---TTGGTGTATAACTACGATACATTGGCAAACCGCCTCAGAGAACTGTCTTATCTCAACAAAGGAATTACAATTACGTTGACCGACGAAAGAGAAAAACTGGAAGACGGCACTTTCCGGACAGAAGTTTTTCATTCGGAAGGTGGTTTGAAGGAATTTGTTGCATATATCGACGGTAACCGTGAATCGATCATGGAGAATGTTATCTTCATGGAAGGTGAACGTGATGATATTCCGGTGGAAGTGGCAATGCGTTACAATACATCTTTCAATGAGAATCTTCATTCCTATGTCAATAATATCAATACGCATGAAGGTGGAACGCACCTGGCGGGTTTCAGAAGAGCTTTAACTAGAACTTTGAAGAAATATGCCGACGAATTGGGACTTCCTGCAAAAGAAAAAGTTGAAGTCACAGGAGACGATTTCCGTGAAGGTTTGACGGCCGTGATCTCTGTAAAAGTAATGGAACCACAATTCGAAGGACAGACTAAAACCAAGTTAGGGAATTCAGAAGTGTCGGGAGCGGTTGATAAAATCGTTGGCGAAATGCTGACCAACTTCCTGGAGGAAAATCCGGCTGAGGCCAAGATCATTGTTCAGAAAGTAGTTTTAGCTGCGAAAGCAAGACAAGCTGCGAAGAAAGCCAGAGAAATGGTACAGAGAAAATCGCCGATGGGCGGCTCCGGATTACCGGGGAAATTGTCTGATTGCTCTTCTAAAGACCCAGCTGAATCTGAGCTATTCCTTGTAGAGGGAGATTCTGCAGGTGGAACGGCTAAACAAGGTCGTGACCGTCATTTTCAGGCGATTTTGCCTTTGAGAGGTAAGATTTTGAATGTCGAGAAATCGATGGTTCACAAGGTTTATGATAATGAAGAAATCAAGAATATCTATACGGCGCTTGGTGTTTCTGTAGGAACGGAAGAAGATTCCAAAGCGCTAAATATGGCAAAACTGAGATATCACAAAATCGTCATTATGACCGATGCGGATATTGATGGTTCTCACATTTCGACTTTGATTCTGACGTTCTTCTTCAGATATATGAAAGAGCTTATCGAGAATGGGTATATTTATATCGCTCAGCCGCCTTTATATTTACTTAAGAAAGGTAATAAAAAGATCTACGCTTATAATGAGAAAGAACGTGAAGAGTTTACTCTTGAGATGTCTCCGGACGGAAAAGGAGTAGAAGTGCAGCGTTATAAAGGTCTTGGGGAGATGAATCCTGAGCAGCTTTGGGAAACAACACTTAACCCTGAACACAGAATCCTTAAACAGGTAACGATTGACAATGCGGTTGAGGCTGATTCAACCTTCTCAATGCTGATGGGGGATGAAGTCCCACCAAGAAGAGAATTCATCGAGAAAAATGCGGTATACGCAAGAATTGATGAATTCTCTATCATCCAGAATACATTACAAAAAGACCCTACCAACTCCGAGAAAGAAGCGGTAGAATATATCTATCGTCAGCTCAGAAATGCTGATGCGCCGGATGAGGAAACCGCAAGAGGAATTATTGAAAAATTATTCTTCTCCGAGCAGAGATATTCACTTGGTGAAGTAGGTCGTTACAGACTAAACAAAAAATTAGGTCTTAATATTTCTGAAGATACTCAGGTTCTGACAAAAGAAGATATCATCTCTATCGTAAAACACTTGATCGAACTGGTGAATTCTAAGGCTGAGGTTGATGATATCGACCACCTTTCCAACAGACGTATCAAAACTGTAGGTGAGCAGTTGTCAGGACAGTTTGGTGTAGGTCTTTCCAGAATTGCAAGAACAATCAAGGAAAGAATGAACGTTAGAGATAACGAAATCTTTACGCCGGTTGATTTAGTTAATGCTAAGACTTTGACATCTGTTATCAATTCATTCTTTGGTACCAACCAGCTTTCTCAGTTCATGGATCAGACCAACCCATTGTCAGAGATCACTCACAAGAGAAGATTATCTGCACTGGGACCTGGTGGTTTATCAAGAGAAAGAGCTGGTTTCGAGGTTCGTGACGTTCACCATACACACTACGGAAGAATTTGTCCGATTGAAACTCCGGAAGGACCGAATATCGGTTTGATATCTTCTTTGGGAATGTATGCGAAAATCAATACGCTTGGTTTCATAGAAACACCTTATAGAAAAGTTGAGAACGGGAAAGTTGATCTTAACGCAAAACCAATCTACCTGAATGCTGAAGATGAAGAATATAAAGTAATTGCTCAGGCAAACGTTGCGTTGAGTGATGACGGTAACTTCGAAACAGACAGGATTATTGCCCGTCTTGATGGAGATTACCCGGTAGTAGAGCCTCAGCAAGTAGATTTGATTGATGTTGCACCAAACCAGATTTCCGGTATTTCTGCATCTTTGATTCCATTCCTGGAGCATGATGATGCGAACCGTGCATTGATGGGGTCGAATATGATGCGTCAGGCAGTTCCATTATTGAAACCTCAGGCACCAATCGTTGGTACAGGTTTGGAAAAACAAGTAGCAAAAGATTCCAGAATCCTGATCAATGCAGAAGGAAGTGGTGTTGTAGAATATGTAGATGCTGAGAGAATCGTAATCAAATACGAAAGAACCGAAGAAGACGATCTTGTCAACTTTGATCCGGCAACGAAAACTTATAAACTTACTAAGTTCAGAAAAACCAACCAGAGCACAACAATTACTCTCAGACCAAACGTAAGAGTAGGTGATAAGGTAGAAAAAGGACAGGTGCTTTGTGACGGTTATGCAACTGAGAACGGAGAATTGGCTCTTGGTAGAAACCTGGTAGTTGCGTTCATGCCTTGGAAAGGATATAACTTCGAGGATGCAATCGTGATTAATGAAAAAGTAGTTCGTGAAGACTGGTTTACTTCGATTCATGTTGACGAATATTCTCTAGAGGTTCGTGATACCAAATTGGGTATGGAAGAACTGACAGCGGATATTCCTAACGTTTCTGAAGAAGCAACCAAAGATCTCGATGAGAACGGTATGATCCGTATCGGTGCAGAAGTGAAGCCTGGTGATATCCTTATCGGAAAAATCACGCCAAAAGGTGAATCTGACCCGACTCCGGAAGAAAAACTTCTTAGAGCAATCTTCGGAGACAAAGCTGGTGACGTGAAAGATGCTTCATTGAAAGCGGATTCTTCTCTCAGAGGAGTTGTTATCAACAAAAAATTGTTCTCCAGAAATATCAAAGACAAAAAGAAAAGAAGCGAAGAGAAAATAAAGTTGGAAGAAATCGAAAATACTTACAAAAATAAGTTCGACGATTTAAGAAATACTTTGATAGACAAACTGAATACTCTGGTTTCCGGTAAAACATCTCAAGGAGTTAAAAATGACCTGGATGAGGAAATGATCGGAAAAGGAACCAAGTTCACTTTGAAATTGCTTCAGTCTGTTGAGGATTATGTAAACATCAGCGGCGCAGACTGGACTGTTGATGCAGACAAAAATGAATTGATCAAGCAATTGATCCACAATTATAAGATTAAATACAACGATCTTTCGGGTGTTAAAAACCGTGAAAAATTTGCATTGTCAATCGGAGATGAGCTACCTGCAGGTATTATCAAATTAGCAAAAGTTTACATCGCTAAGAAGCGTAAACTGAATGTTGGAGATAAGATGGCTGGTCGTCACGGTAACAAAGGTATCGTTTCAAGAATCGTTCGTGAAGAAGATATGCCGTTCCTGGAAGACGGAACACCGGTTGATATCGTATTGAATCCACTTGGGGTACCTTCTCGTATGAACATCGGTCAGATCTATGAAACTGTTCTTGGATGGGCTGGTAAACAGCTTGGATTGAAATTCGCAACGCCGATCTTCGACGGAGCTAGTTTAGATCAAATTACCGAGTACACCGAGCAGGCAGCACTTCCTAAATTCGGAAACACGCACCTTTACGACGGTGGAACAGGTGAGAGATTTACGCAGCCGGCAACCGTTGGTGTGATCTATATGTTGAAGCTGGGACACATGGTAGATGATAAGATGCACGCGCGTTCTATCGGACCTTACTCATTGATTACGCAGCAGCCGTTAGGAGGTAAAGCGCAATTCGGAGGTCAGAGATTCGGAGAGATGGAGGTTTGGGCTCTTGAAGCATTCGGAGCATCGAATATTCTTAGAGAGATCCTGACTGTGAAGTCGGATGACGTGATTGGTAGAGCAAAAACTTATGAAGCGATTGCAAAAGGAGAAGCAATGCCAGAACCTGGTATTCCGGAATCTTTCAACGTATTACTTCACGAGTTACAAGGTCTTGGACTTGATGTAAGAATCTTACTTCTGGAATTATGTTTCAGCCTTTTGATCGCTTTTTCCTTGATCTGACGAACTCTTTCTCTTGTTAAATCGAAGGTTTCACCAATTTCTTCCAATGTCATAGGATGTTTCCCGTTCAGACCGAAATATAATCTTACAAGATCCGCTTCTCTTGGTGTGAGGGTATTCAAGGCTCTTTCAATCTCAATTTGAAGAGATTCAAGCATCAGATCTTTATCCGGACTTGGCGATTCTCCGGAACGTAACACGTCATAAAGGTTAGAATCTTCACCTTCCACAAGCGGTGCATCCATCGATAGGTGACGACCACTGTTTTTCATAGATTCTTTGATGTCTTCCTCGCTCATATCCAGAACTTCTGCCAATTCTTCCGGAGAAGGCGGTCTTTCGTTCTCTTGCTCCAAGTGAGCATAAGCTTTGTTGATTTTGTTGATAGAACCAATCTTATTCAAAGGTAATCTTACAATTCTCGACTGCTCAGCCAAAGCCTGCAAAATCGATTGACGAATCCACCAAACAGCATAAGAAATGAATTTGAAACCTCTCGTTTCATCATATCTTTTCGCCGCTTTCATCAGACCAAGATTTCCTTCATTGATTAGATCCGGAAGAGAAAGTCCCTGATTCTGATACTGTTTAGAAACCGAAACCACGAAACGTAAGTTGGCTTTGATCAGTTTTTCAAGAGCAGCTCTGTCTCCGGCACGGATTCTCTGAGCCAAATCTACCTCCTCATCGGCGGTAATCAATTCAACTTTTCCAATCTCTTGCAGATACTTGTCCAGTGATGCGGTTTCCCTGTTGGTAACCTGTTTTGTAATTTTTAATTGTCTCAT

>Chryseobacterium_taklimakanense_CCTCC AB 208154T

AGTATCCAGGCACTGGAAGGAATGGAGCATGTAAGGATGCGTCCGTCAATGTACATTGGAGATGTGGGTCTAAGAGGTTTGCACCATTTGGTTTATGAGGTGGTGGACAATTCCATTGACGAAGCATTGGCCGGGTATTGCGATACCATTACCGTAACCATCAAAGAAAGCAACGCCATAGAGGTGACCGACAACGGTCGCGGTATCCCGGTAGATTACCACGAAAAAGAGCAAAAATCCGCCCTTGAAGTTGTAATGACAAAAATCGGTGCCGGTGGTAAGTTCGATAAAGATTCTTATAAAGTATCAGGCGGGCTTCACGGTGTGGGAGTTTCATGTGTCAATGCGCTTTCAACCGAAATGGTGACTACGGTTTACAGAGACGGTAATGTTTACCAGCAAACCTATTCCAGAGGTAAAGCGCAAACCGATGTAAAAGAAGTTGGAAAAAGCGACCAAA-GAGGGACAAAACAGTTCTTCCAGCCGGACGACACCATCTTCACGGAG---CTCGTTTACAATTACGATACTTTGGCGAGCAGGCTTCGCGAACTTTCTTATCTGAATAAAGGTATAACGATTACTTTAACCGATGAAAGAGAGCGCCAGGAAGACGGAACTTTCAAATCTGAATCCTTCTATTCGGAAGGTGGTTTAAAGGAATTTGTAGAATATATTGACGGAAACCGCGAATCTATTATGGAAAATGTGATTTTCATGGAAGGCGAGCGCGACAATATTCCCGTTGAGGTAGCCATGCGCTACAACACGTCTTTCAACGAGAATTTACATTCCTACGTCAACAATATCAACACCCACGAAGGTGGAACGCACCTCGCAGGATTCAGAAGGGCTTTGACAAGAACTCTGAAAAAATTCGCCGATGAGCTCGGGCTTCCGGCTAAAGAAAAAGTGGAGGTTACCGGTGATGATTTCCGTGAAGGTTTGACCGCTGTGATTTCGGTAAAAGTAATGGAACCTCAGTTCGAAGGTCAGACGAAGACTAAATTAGGAAATTCCGAAGTTTCCGGTGCGGTGGATAAAATCGTTGGTGAAATGCTTACCAATTTCCTTGAGGAAAATCCCAACGAAGCAAAGCAGATTGTACAGAAAGTGGTTCTTGCCGCAAAAGCGAGACAGGCTGCGAAAAAGGCGAGAGAAATGGTACAGCGCAAATCGCCGATGGGCGGTTCCGGACTTCCGGGAAAACTGGCAGACTGCTCGTCTAAAGACCCGGAAATTTCTGAGATTTTTCTGGTAGAGGGGGATTCCGCGGGTGGAACTGCAAAACAGGGACGCGACCGTCATTTCCAGGCCATTCTTCCCTTAAGAGGTAAGATTTTGAACGTAGAAAAATCAATGCTTCACAAGGTTTACGATAACGAGGAGATCAAAAATATCTACACGGCACTGGGTGTTTCTGTAGGAACTGAAGAAGACAGCAAGGCCCTGAATTTAAGCAAATTAAGATATCATAAAGTGGTGATTATGACCGATGCCGATATCGACGGTTCGCACATTGCGACTTTGATTCTGACGTTCTTCTTCCGTTATATGAAAGAACTGATTGAGCATGGTTATGTGTATATCGCTTCGCCGCCGCTTTATTTGTTGAAAAAAGGCAACAAGAAAGTATATGCCTGGAATGAGAAAGAAAGAGAAGAAAAAACACTCGAAATGTCTCCTGACGGGAAAGGAGTAGAGGTGCAGCGTTACAAAGGTCTCGGCGAGATGAATGCGGAACAGCTCTGGGAAACTACCCTTAATCCGGATAACAGAACCCTGAAACAGGTAACCATTGATAGCCTTGCAGATGCAGATAATGTATTTTCGATGCTGATGGGTGATGAGGTGCCGCCAAGAAGGGATTTTATCGAGAAAAATGCAAAATACGCAAGAATTGATGAATTCTCTATCATTCAGAATACATTACAGAAAGACCCTACAAACTCTGAAAAAGAGGCTGTAGAGTACATCTACCGTCAGTTGCGTAACGCAGATCCACCTGATGAGGAAACGGCAAGAGGAATCATTGAAAAACTGTTCTTCTCAGAGCAGCGTTATTCTTTAGGTGAGGTAGGGCGTTACCGTCTGAATAAAAAATTAGGTTTAAATATTCCTGAAACGACTGAAGTCCTTACCAAAGACGACATCATCGCCATCGTGCGCCACCTGATCGAGTTGGCAAATTCAAAAGCGGAAGTTGATGATATCGACCACCTTTCCAACAGAAGAATCAAGACTGTTGGCGAGCAGTTGGCAGGTCAGTTCGGTGTTGGTCTTTCCAGAATTGCAAGAACCATCCGCGAGAGAATGAACGTGCGTGATAACGAGATTTTCACGCCGGTTGATTTGGTTAATGCTAAAACTTTAACTTCAGTAATCAACTCGTTCTTTGGTACTAACCAACTTTCTCAGTTCATGGACCAAACGAACCCGCTTTCAGAAATCACTCACAAGAGAAGACTGTCTGCATTGGGGCCTGGCGGTCTTTCAAGAGAAAGAGCAGGTTTCGAGGTACGGGACGTTCACCATACACACTACGGAAGAATCTGTCCGATTGAAACTCCGGAAGGGCCAAACATCGGTTTGATTTCTTCTTTGGGTATCTATGCTAAAATCAACAACTTAGGTTTCATTGAAACACCATACAGAAAAGCAGAAAACGGTAAAATTAATCTAAATGAAGAGCCAATTTACCTGAATGCTGAAGATGAAGAGGCCAAAGTGATCGCTCAGGCAAACGTTGAGATGAATGATGACGGTACGATTGCTACCGAAAGGGTAATTGCGCGTTTGGATGGTGACTATCCGGTAGTTGAACCCAACCAGGTTGACCTTATCGACGTAGCACCGAACCAGATTTCCGGTATTTCCGCTTCTCTTATTCCGTTCCTTGAGCATGATGATGCGAACCGTGCATTGATGGGATCCAACATGATGCGTCAGGCCGTTCCGTTATTGAAGCCTGAGGCTCCAATCGTTGGTACAGGGCTTGAAAAACAGGTCGCGAAAGACTCCAGAATTTTGATTAATGCAGAAGGTAACGGTGTTGTAGAATATGTAGATGCAGACAAAATCGTCATCAAATACGAAAGAAGCGAGGATGAAGACATCGTAAGCTTCGAATCTGCAACTAAAACTTACAATCTTACCAAGTTCAGAAAAACCAACCAAAGTACTACCATTACACTTCGCCCGAACGTAAGAGTTGGCGATAAAGTGGTTGAAGGGCAGGTGCTTTGCGACGGTTATGCTACTGAAGACGGTGAATTGGCACTGGGTAGAAACCTTGTTGTAGCGTTCATGCCTTGGAAGGGATACAACTTCGAGGATGCGATTGTAATCAACGAAAAGGTTGTTCGTGAGGACTGGTTTACTTCCATCCACGTAGATGAATATTCACTTGAAGTTCGCGATACCAAATTAGGTATGGAAGAACTTACTGCTGATATCCCTAACGTGTCCGAAGAGGCTACAAAAGACCTTGATGAAAACGGGATGATCCGTATTGGTGCTGAGGTGAAGCCTGGCGATATCATGATCGGTAAGATCACGCCAAAAGGTGAATCTGACCCGACGCCGGAAGAAAAACTTCTTCGTGCGATCTTCGGTGATAAAGCCGGTGATGTGAAAGATGCTTCGTTGAAAGCCGACTCTTCATTGAGAGGTGTGGTTATCGACAAGAAACTTTTTTCCAGAAACATCAAGGATAAAAAGAAAAGAGCTGAAGAAAAGCTTAAACTTGAGGAAATCGAAAACAGCTACAAAGCGAAATTCGATGATTTAAGGAATACGTTAATCGAAAAACTGAACACTTTAGTAAGCGGTAAAACTTCACAGGGGGTAAACAACGACCTGAACGAAGAAATTATCGGTAAAGGGGTGAAATTCACGCTGAAACTGCTTCAGTCTATCGACGATTATGTAAACGTAAGCGGTGCCGACTGGACTGTTGATGCAGACAGAAACGACCTGATTAAGCAGTTGATCCACAACTATAAAATTAAATACAACGATATCGCCGGAGTTAAAAACCGTGAGAAATTCGCAATTTCAATCGGGGACGAACTTCCTGCAGGTATTATGAAACTGGCTAAAGTTTACATTGCTAAGAAACGTAAACTGAACGTTGGGGATAAGATGGCAGGACGTCACGGTAACAAAGGTATCGTTTCACGTATCGTTCGTGAAGAAGATATGCCGTTCCTTGAGGATGGAACCCCTGTTGATATCGTACTGAACCCGCTTGGGGTACCATCCCGTATGAACATCGGCCAGATCTACGAAACGGTTCTTGGCTGGGCAGGTAAAAAACTGGGAATGAAGTTCGCAACGCCAATCTTCGACGGTGCGTCTCTTGAGCAGATTACTGAATACACAGACCAGGCAGGTCTTCCGAAATTCGGAAACACTTATCTGTACGACGGTGGAACCGGTGAAAGATTCACTCAGCCGGCAACGGTTGGTGTGATTTACATGCTGAAACTCGGCCACATGGTTGATGACAAGATGCACGCAAGATCAATCGGTCCATACTCACTCATTACACAGCAGCCGCTTGGAGGTAAAGCACAGTTTGGTGGTCAGCGTTTTGGTGAGATGGAGGTTTGGGCACTTGAAGCATTCGGTGCATCCAACATCCTGAGAGAAATCTTGACCGTTAAGTCTGATGACGTGATTGGTAGAGCGAAAACTTACGAAGCCATCGCCAAAGGTGAAGCAATGCCGGAACCGGGAATTCCTGAATCGTTCAACGTATTGTTGCACGAGCTTCAGGGTCTTGGATTGGACGTGAGAATCTTGCTTCTGGTATTGTGTTTCAGTCTTTTGATGGCTTTTTCTTTAATCTGGCGCACTCTTTCTCTCGTAAGGTCAAAAGTTTCACCTATTTCCTCCAAAGTCATCGGGTGTTTCCCGTTCAGTCCGAAATACAGTCTTACCAAATCAGCTTCTCTTGGCGTAAGCGTTTGTAATGCTCTTTCAATCTCAATTTGCAGAGATTCCAGCATCAGGTCTTTATCAGGGCTTGGCGATTCACCGGAGCGCAAAACGTCGTAGAGGTTGCTGTCTTCTCCTTCCACAAGCGGTGCATCCATTGAAAGGTGGCGGCCGCTGTTTTTCATGGATTCCTTAATGTCGTCTTCGCTCATGTCCAGTACTTCAGCCAGTTCTTCCGGGGAAGGCGGTCTTTCATTTTCCTGTTCAAGGTGAGCGTAAGCTTTGTTAATCTTGTTGATGGAACCAATTTTGTTGAGCGGAAGCCTTACAATTCTCGACTGTTCTGCCAGTGCCTGCAAAATCGACTGACGAATCCACCAAACAGCGTATGAAATAAATTTGAAACCTCTGGTTTCATCGTATCTTTTAGCGGCTTTCATCAGTCCCAGATTTCCTTCATTGATCAAATCCGGTAGTGAAAGTCCCTGATTTTGGTACTGTTTGGATACTGAAACGACGAAACGAAGGTTGGCCTTGATGAGTTTTTCCAGTGCGGCTCTGTCGCCGGCGCGGATTCTTTGTGCCAGTTCTACTTCCTCGTCTGCGGTGATCAGTTCCACTTTCCCGATTTCCTGTAGGTACTTGTCTAGCGATGCGGTTTCCCTGTTGGTAACCTGTTTAGTAATTTTTAGCTGTCTCAT

>Flavobacterium_haoranii_DSM 22807T

AGTATTCAGGCGCTTGAAGGAATGGAGCACGTACGTATGCGTCCTTCCATGTATATCGGTGATACTGGTGTTAGAGGTTTACACCATTTAGTATATGAGGTGGTTGATAACTCTATCGATGAAGCTTTAGCTGGACATTGTGATACGATTAAAGTTACCATAAACGAAGATAATTCTATTACTGTTGAAGATAACGGTCGTGGTATTCCGGTAGATATTCATAAAAAAGAAGGTGTTTCTGCTCTTGAAGTTGTTATGACGAAAATTGGAGCTGGAGGTAAATTCGATAAAGATTCTTATAAAGTTTCTGGTGGACTTCACGGTGTTGGGGTTTCGTGTGTTAATGCACTTTCTGATCATTTACAAGCTACTGTTTTCAGAGAAGGAAAAATTTGGGAACAAGAGTATGAAAGAGGTAAAGCTTTATATCCGGTAAAACAAATTGGAACTACTGATAAAA-GAGGAACTACGGTTACTTTTAAACCAGATGGTACAATTTTCACACAAACTTTAGTTTATTCTTACGATACATTAGCGGCTCGTTTACGTGAGCTTTCTTTCTTAAATAAAGGGATTACAATTACTTTAGTTGATAAAAGAGAAACTGATAAAGACGGTAATTTCTTAGGAGAAACTTTCCATTCACAAGAAGGTTTAAAAGAATTCGTTAAGTTTTTAGATGGAAACCGTGTGCCAATTATCGGTCACGTAATTTCAATGGAAAACGATAAAGGCGAAATTCCAGTTGAAGTAGCTTTAATTTATAACGATAGTTATTCAGAAAATATATTTTCTTACGTAAATAATATCAATACTCACGAAGGAGGAACGCATTTACAAGGTTTCCGTATGGGATTAACGCGAACGTTGAAAAAATATGCCGATGCTTCTGGGTTGTTAGATAAATTAAAATTCGAAATTTCTGGAGATGACTTCCGTGAAGGTTTAACAGCAATTATTTCTGTAAAAGTTGCAGAACCTCAATTCGAAGGTCAAACCAAAACAAAACTTGGAAATAGAGAAGTAGTTTCTCCAGTTTCGCAAGCCGTTGCGGAAATGTTAGAGAATTATTTGGAAGAAAATCCAAATGATGCTAAAACTATCGTTCAAAAAGTAATTTTAGCTGCTCAAGCACGTCACGCTGCTAAAAAGGCACGCGAAATGGTGCAACGTAAAACCGTGATGGGTGGCGGAGGATTGCCAGGTAAATTATCAGATTGTTCGGAACAAGATCCAGAAAAATGCGAAATTTTCCTTGTTGAGGGTGACTCGGCGGGTGGAACTGCAAAACAAGGACGTGATAGATTTTTCCAAGCTATTTTACCTTTACGAGGTAAGATTTTGAACGTGGAAAAAGCGATGCAACATAAAGTATTTGAAAACGAAGAAATTCGAAATATTTTTACAGCTTTAGGGGTTACAGTAGGTACAGAAGAAGATAGTAAAGCGTTGAATTTATCAAAATTACGTTATCATAAAGTAGTGATCATGTGTGATGCCGATGTCGATGGTTCTCACATTTCTACATTAATTTTAACGTTCTTCTTCCGTTACATGAAAGAACTTATTGAAGGAGGACACGTTTATATTGCGCAACCACCTTTATATTTAGTTAAAAAAGGAAACAAGAAAGCGTATGCTTGGAATGATGAGCAACGCGATCAAATTTCTACTGAAT---------ACGGTGGAGGAGCAAACGTTCAACGATATAAAGGTCTTGGAGAGATGAACGCTGAGCAGTTATGGGAAACTACATTAAATCCTGAATTTAGAACGCTTCGTCAGGTTACTATTGATAGTTTATCTGAAGCTGATAGAGTTTTCTCTATGTTAATGGGAGATGAAGTGCCACCTCGTAGAGAGTTTATTGAGAAAAATGCTGCTTACGCTAAGATTGATGATTACACAATTATTCACAATACATTACAAAAAGACCCAACAAACTCTGAAAAAGAAGCTGTTGAGCATATATATAGACAATTACGTAATGCTGAACCACCTGATGAGGAGACAGCAAGAGGAATAATTGATAAATTATTCTTCTCTGATCAACGTTATAATTTAGGTGATGTAGGACGTTACAGAATGAATAAAAAGTTAGGCTTAGATATCCCAATGGATAAACAAGTATTAACTAAGGAAGATATTATTACTATCGTTAAGTATTTAATTGAGTTAATCAATTCAAAAGCTGAGATTGATGATATCGACCACTTATCAAACCGTCGTGTAAGAACAGTAGGTGAGCAATTATCAGCTCAGTTTGGTGTTGGTTTAGCACGTATGGCAAGAACTATTCGTGAGAGAATGAACGTTCGTGATAATGAGGTGTTTACACCAATCGACTTGATTAATGCTAAAACATTATCATCAGTTATCAATTCATTCTTTGGTACAAACCAGTTATCTCAATTTATGGACCAAACGAATCCATTAGCAGAGATTACTCACAAACGTCGTTTATCTGCTTTAGGACCTGGAGGTTTATCTAGAGAAAGAGCTGGTTTCGAGGTACGTGACGTTCACTATACGCACTACGGACGTTTATGTCCAATTGAAACTCCAGAGGGACCAAACATTGGTTTGATTTCATCTTTAGGGGTTTATGCAAAAGTAAACGGAATGGGATTCATCGAAACTCCTTATCGTAAAGTTGAAAACGGTGTTGTAGATCTTAAAAACGATCCAATTTATTTAAGTGCTGAAGAAGAAGAAGGTAAATTAATCGCTCAGGCTAACATTGAAATGACTACTGAAGGTCAAATCACTGCTGAAAGAGTAATTGCTCGTGAAGAAGGTGATTTCCCAGTTGTAGAACCAAATACAGTTCATTATACTGACGTTGCACCAAACCAAATTGCTTCAATCTCGGCTTCATTAATTCCGTTCTTAGAGCATGATGATGCGAACCGTGCGTTGATGGGATCAAACATGATGCGTCAAGCGGTTCCTTTATTACGTCCTCAAGCTCCAATTGTTGGAACTGGATTAGAAAGACAAGTTGCTTCAGATTCAAGAGTTTTAATTAACGCTGAAGGGGATGGAGTTGTAACTTATGTAGATGCTGAAAAAGTAACTATTAAATACGATAGAACTGAAGAAGAAAGAATGGTAAGTTTTGACGAAGACGATAAAACTTATTTCTTAATTAAATTCAGAAAAACCAACCAAAGTACTTCTATCAACTTAAAACCAATCGTAAGAAGAGGTGATAGAGTTGTTAAAGGCCAAGTTCTTTGTGAAGGTTATGCAACTCAAAACGGAGAATTAGCTTTAGGTAGAAACTTACAAGTTGCCTTCATGCCTTGGAAAGGATATAACTTCGAGGATGCAATTGTAATTTCTGAAAAAGTTGTTCGTGATGATATTTTTACATCAATTCACATTGATGATTATTCATTAGAAGTACGTGATACAAAATTAGGTAACGAAGAGTTAACTAACGATATTCCTAACGTTTCTGAAGAGGCTACTAAAGACTTAGATGAAAACGGTATGATTCGTATCGGTGCTGAAGTAAAACCTGGTGATATCTTAATTGGTAAGATTACTCCAAAAGGAGAGTCTGATCCAACACCAGAAGAAAAATTACTTCGTGCAATCTTTGGTGACAAAGCTGGTGATGTTAAAGATGCTTCATTAAAAGCTTCTCCATCATTAAGCGGTGTAGTTTTAGACAAAAAATTATTTGCTAAAGCTGTAAAAGATAAACGTAAACGTTCTAAAGATAAAGAAGACGTTGATAAACTAGAAATGGAGTTTGACGTTAAATACAACGAATTAAAAGATAAATTAATTGAAAAATTATTTATCATCGTTGACGGAAAAACTTCTCAAGGTGTTATCAATGATTTAGGTGAAGAAGTATTACCAAAAGGTAAAAAATTCACTAAAAAGATGTTACAAGCAGTTGATGATTTTGCTCACTTAACAAAAGGTCAATGGACTACTGATGAGCATACAAATAAATTAATCAACGAACTTGTTCATAACTATAAAATCAAGTTAAACGACTTACAAGGATGGTTAAGAAGAGAAAAATTCACCATCACTGTAGGAGATGAGTTACCATCTGGAATCTTAAAACTTGCTAAAGTTTACATTGCTAAAAAACGTAAACTTAAAGTAGGAGATAAAATGGCGGGACGTCACGGTAATAAAGGTATTGTTGCGAAAATTGTTCGTCAAGAAGATATGCCTTTCTTAGAAGACGGAACACCAGTAGATATCGTATTGAATCCACTAGGGGTACCTTCTCGTATGAACATTGGTCAGATTTATGAAACTGTTTTAGGTTGGGCAGGTTTAAAACTTGGTAAAAAGTTTGCAACTCCAATCTTTGATGGTGCTACATTAGATGAAATCAATGCTTTAACTGATGAAGCTGGTATTCCAAGATTCGGTCATACTTATTTATATGATGGTGGAACTGGAGAGCGTTTCCATCAGCCAGCAACTGTGGGTGTAATTTACATGTTGAAATTAGGTCACATGGTTGATGATAAGATGCACGCACGTTCTATCGGACCTTACTCTTTAATTACGCAACAACCATTAGGAGGTAAAGCTCAATTCGGAGGTCAGCGTTTTGGAGAGATGGAGGTTTGGGCTCTTGAAGCTTATGGTGCTTCAAGTACCTTGAGAGAGATCTTAACGGTTAAATCTGATGACGTAATTGGTAGAGCTAAAACTTACGAGTCAATCGTAAAAGGTGAACCAATGCCAGAACCAGGATTACCTGAGTCATTCAATGTATTAATGCATGAATTAAAAGGTCTAGGTTTAGACATCAGAATTTTACTTCTAGAAGTGTGTTTTAATCTGCGAATTGCTTTTTCTTTAATTTGGCGAACACGTTCACGCGTTAAATCGAAAGTTTCTCCTATTTCTTCAAGAGTCATTGGATGTTGATCACCTAATCCAAAATATAAACGAATAACATCAGCTTCTCTTGGAGTTAATGTTTCTAAAGCTCTTTCAATCTCAGTTTGTAAAGACTCGTGAATTAATTCTCTATCAGGATTTGGAGATTCTCCAGAACGTAATACGTCATATAAGTTAGAATCTTCACCTTCAACTAAAGGTGCATCCATTGATAAATGACGACCAGAATTTTTCATAGATTCTTTAACGTCATTAACTGTCATGTCTAATTCTTTTGCAATTTCTTCGGCAGAAGGAGCACGTTCACTAGATTGTTCTAACAACGCGTACATTTTATTGATCTTGTTGATAGAACCAATTTTATTTAGTGGTAAACGTACAATACGAGATTGTTCTGCTAAAGCTTGTAAGATAGATTGACGAATCCACCATACAGCATATGAAATAAATTTAAAACCACGAGTTTCATCAAAACGCTGAGCCGCTTTAATTAATCCTAAGTTTCCTTCGTTAATTAAATCGGGTAATGTTAATCCTTGGTTTTGATATTGTTTAGCAACCGAAACTACGAAACGTAAATTAGCTTTAGTTAATTTTTCTAAAGCTCTTTGATCGCCTGCTTTAATTCTTTGTGCTAATTCTACCTCTTCATCTGCAGTAATTAAATCAACTTTACCAATTTCTTGTAGGTATTTGTCTAAGGAAGCAGTTTCACGATTAGTTACCTGCTTAGTAATTTTGAGCTGTCTCAT

>Chryseobacterium_lacus_XH07

AGTATTCAGGCACTGGAAGGAATGGAGCACGTTCGTATGCGTCCTTCCATGTACATTGGTGATGTCGGTTTAAGAGGTCTTCACCATTTGGTTTATGAAGTGGTGGATAACTCTATTGATGAGGCATTGGCGGGACACTGCGACACCATTTCTGTGGTCATTCACGAAGGTGAAAGTATTTCAGTAAAAGATAATGGCCGTGGAATTCCGGTTGATTTTCACGAAAAAGAGCAGAAATCCGCTTTGGAAGTGGTCATGACCAAAATTGGAGCCGGTGGAAAATTTGATAAAGATTCTTACAAAGTTTCCGGTGGACTCCACGGAGTAGGAGTTTCCGTAGTGAATGCACTTTCCAATTCCTTGATCGCAACCATTAATCGGGACGGTAAAATTTATCAGCAAAAATATTCCAAAGGAAAAGCTTTAGGAGATGTAAAAGAAATCGG-AACAACCACAGAACGGGGTACCGAAGTTTTTTTTCAAGCCGATGATACCATTTTTCAGGAA---TTAATCTTTAATTACGATACTTTAGCAAGCAGAATGCGTGAGCTTTCTTACTTGAACAAGGGCATTAAAATTACCTTAACAGACGAAAGAGAAAAAAACGAAGAAGGTAATGCTAAGCAAGACATCTTTTTTTCTGAAGGGGGTTTAAAAGAATTCGTAGAATACATCGATGGAAATCGCGAAGCCATTATGGACAGCGTCATCTTTATGGAAGGCGAACGCGAGGGAATTCCTGTGGAAGTGGCGATGCGTTATAACACATCTTTTAACGAAAATCTGCATTCCTATGTTAATAATATTAATACCCATGAAGGCGGAACCCATTTAGCCGGGTTCAGAAGAGCTTTAACGAGAACTTTAAAGAAATTCGCCGATGAACTGGGACTTCCTGCCAAAGAAAAAGTAGAAGTTACGGGTGACGATTTCCGCGAAGGATTGACGGCTGTAATTTCCGTAAAAGTAATGGAACCGCAGTTTGAGGGACAAACCAAAACAAAACTAGGAAACTCTGAGGTTTCGGGCGCAGTGGATAAGATCGTAGGCGAAATGCTGACCAACTTCCTGGAAGAAAATCCTAATGAAGCCAAGATCATCGTTCAAAAAGTAGTATTAGCGGCTAAAGCCCGACAAGCCGCCAAAAAAGCGAGAGAAATGGTACAGCGCAAATCGCCGATGGGCGGTGCTGGACTTCCGGGAAAACTGGCTGACTGTTCTTCCAAAGATCCTTCGATTTCTGAATTATTCCTCGTGGAGGGTGATTCCGCAGGCGGAACGGCTAAACAAGGCCGCGACCGTCATTTTCAGGCGATTCTTCCTTTGAGAGGTAAAATTCTGAATGTTGAGAAATCCATGCTTCATAAAGTTTACGAGAACGAAGAAATTAAAAATATTTATACGGCACTCGGTGTTTCCATAGGAACTGAAGAAGATAGCAAGGCTTTGAATATTAGCAAATTAAGATACCATAAGGTAGTAATTATGACAGATGCTGATATTGACGGATCTCACATTTCCACCTTGATTCTTACTTTCTTTTTCAGATATATGAAAGAATTGATTGAACAGGGATACATTTATATCGCTTCTCCGCCGCTTTATCTTTTGAAAAAAGGAAATAAAAAAGTGTATGCTTGGAATGAAAAAGAAAGAGAAGAAAAAACGCTGGAAATGTCTGCCGACGGCAAAGGAGTAGAAGTTCAGCGATACAAAGGTCTTGGGGAAATGAATGCAGAACAGCTTTGGGAAACCACCCTGAACCCAGATAACAGAACTTTGAAACAAGTGACCATTGAAAGCTTGGCAGATGCAGATAATGTATTTTCGATGCTGATGGGAGATGAGGTTCCGCCAAGAAGGGATTTTATTGAGAAGAATGCCCGCTACGCTAGAATTGATGAATTTTCTATCATCCAGAATACATTACAAAAAGACCCTACAAACTCTGAAAAAGAGGCTGTGGAATATATCTACCGTCAGCTGAGAAATGCAGATCCGCCAGATGAGGAAACGGCAAGAGGAATTATTGAGAAACTATTCTTTTCTGAACAAAGATATTCCCTAGGTGAGGTTGGGCGCTACCGTCTGAACAAAAAACTAGGATTAAATATCCCTGAAACCACAGAAGTTCTAACCAAGGACGACATCATCGCTATTGTTCGCCACCTTATTGAGTTGGCCAACTCTAAAGCGGAAGTGGACGATATTGACCACCTTTCTAACAGAAGGATTAAAACGGTTGGTGAGCAGTTGGCAGGACAATTCGGCGTTGGTCTTTCCAGAATTGCAAGAACAATTCGCGAGAGAATGAATGTGCGTGATAACGAGATTTTTACGCCAGTGGATTTGGTGAATGCGAAGACATTAACTTCCGTAATCAACTCTTTCTTTGGTACCAATCAGCTTTCTCAGTTTATGGACCAAACGAACCCATTGTCGGAAATCACACATAAGAGAAGATTGTCAGCTTTAGGGCCTGGTGGCCTTTCAAGAGAAAGAGCAGGTTTCGAGGTTCGAGATGTTCACCATACGCACTACGGAAGAATCTGTCCGATTGAAACTCCGGAAGGACCGAACATTGGTTTAATTTCGTCATTAGGTATCTATGCGAAAATCAACAACTTAGGGTTTATTGAAACGCCCTACAGAAAAGTAGAGAACGGAAAAGTAGATCTGAAGGCGAAACCGATATATCTGAATGCAGAAGATGAAGAGGATAAAGTTATCGCACAGGCTAACGTTGACCTGAACGAAGACGGAGTGTTCGTAACAGACAGAATTATTGCCCGTTTGGATGGTGATTATCCTGTAGTTGAACCTCAGCAGGTAGACCTTATCGATATTGCTCCAAATCAGATTTCCGGTATTTCTGCATCCTTGATTCCGTTTTTGGAACATGACGATGCGAACCGTGCCTTGATGGGATCCAACATGATGCGTCAGGCAGTGCCATTATTGATGCCGGATGCGCCGATTGTAGGAACAGGTCTTGAAAAACAAGTAGCAAAAGATTCCAGAATTCTGATTAATGCAGAAGGATCCGGAATTGTAGAATATGTAGATGCTGATAAAATTGTTATTAAATATGACAGAAGTGAAGATGAAGACATTGTAAGCTTCGAATCGGCAACTAAAACTTATAATCTAACCAAATTCAGAAAAACCAACCAAGGTACAACCATTACTTTGAGGCCGAACGTAAGAGTAGGCAATAAAGTTCATGAAGGACAGGTGCTTTGCGACGGTTATGCAACTGAAGATGGCGAGTTGGCGTTGGGTAGAAACCTTACCGTAGCATTCATGCCATGGAAAGGATATAACTTTGAGGATGCCATCGTAATTAACGAAAAAGTGGTTCGTGAGGACTGGTTTACGTCGATTCACGTAGATGAATATTCTCTTGAAGTTCGTGATACCAAATTAGGAATGGAAGAACTCACGGCAGATATCCCAAATATCTCAGAAGAGGCTACCAAAGACCTTGATGAAAACGGTATGATTAGAATTGGAGCCGACGTGAAGCCTGGCGACATTATGATCGGTAAAATTACGCCTAAAGGTGAATCTGATCCTACACCGGAAGAAAAATTACTGAGAGCGATCTTTGGTGATAAAGCCGGCGATGTGAAAGATGCCTCCCTGAAAGCAGATTCTTCATTAAGAGGAGTGGTGATCAACAAAAAGCTGTTCTCCAGAAATATCAAGGATAAAAAGAAAAGAGCGGAAGAAAAACTTAAATTGGAAGAAATCGAAAACGACTACAAAGCAAAATTCGACGATTTAAGAAATACGCTGATCGAAAAACTTGGGACTTTGGTAAATGGGAAAACTTCACAAGGCGTGAATAACGATTTGAGTGAAGAAATCATTGGAAAAGGAGTTAAATTTTCTACCAAATTGTTACAAAGTGTTGAAGATTACGTGAATGTTAGCGGTTCCGACTGGACGGTTGATAATGACAAGAACAATCTTATTAAACAATTGATTCATAACTATAAAATCAAGTATAATGACATTGCCGGTGTGAAAAACAGAGAGAAATTTGCTGTTTCCATCGGGGATGAACTTCCTGCAGGAATCATGAAACTTGCCAAAGTTTATGTCGCTAAAAAACGTAAACTGAATGTTGGTGATAAAATGGCAGGGCGTCACGGAAACAAAGGTATTGTTTCACGTATTGTTCGTGAAGAAGATATGCCGTTCCTAGAAGACGGAACTCCAGTAGATATCATTTTGAATCCGCTAGGTGTGCCTTCACGTATGAACATCGGACAGATTTATGAAACTGTTCTTGGATGGGCCGGTCAAAAACTGGGTCTTAAGTTTGCTACTCCGATTTTCGACGGTGCATCATTGGATGACATCACGAAATATACGGATGAAGCAGGTTTGCCAAGATACGGAAGTACCTATCTGTACGACGGTGGAACCGGTGAAAGATTCACACAACCGGCAACTGTAGGTGTGATTTATATGTTGAAATTAGGCCACATGGTGGATGACAAGATGCACGCACGTTCTATCGGTCCTTATTCATTGATTACGCAGCAACCACTCGGTGGTAAAGCGCAATTCGGTGGACAGCGTTTCGGGGAGATGGAAGTTTGGGCTCTGGAAGCATTTGGTGCATCCAACATCCTGAGAGAAATCTTAACCGTGAAGTCTGATGACGTGATTGGTAGAGCGAAAACTTATGAAGCCATCGCCAAAGGTGAAGCAATGCCAGAACCTGGAATTCCTGAATCTTTCAACGTATTGTTGCACGAACTGCAGGGTCTTGGTCTGGATGTGAGAATTTTACTTCTCGTATTGTGTTTCAGCCTTTTGATGGCTTTTTCTTTAATCTGGCGAACGCGCTCACGGGTAAGGTCGAAGGTTTCTCCGATTTCTTCCAGCGTCATCGGATGTTTTCCGTTCAGTCCGAAATAGAGGCGCACCAGATCCGCTTCTCTTTGGGTCAGCGTCTGCAGTGCTCTTTCAATTTCAATCTGAAGGGATTCCAGCATCAGGTCCTTATCCGGGCTTGGCGATTCGCCGGAACGCAACACATCGTATAAATTGGAATCTTCACCTTCTACCAGCGGGGCATCCATCGAAAGGTGACGGCCGCTGTTTTTCATGGATTCCTTGATGTCTTCTTCGCTCATATCCAGCACTTCTGCCAGTTCTTCCGGGGAAGGAGGCCTTTCGTTTTCCTGCTCCAGGTGGGCGTACGCTTTGTTGATTTTATTGATGGAACCAATTTTGTTCAAAGGAAGCCTTACAATTCTCGACTGTTCCGCCAGTGCCTGAAGAATGCTCTGCCTGATCCACCAAACGGCGTAGGAAATGAATTTAAAACCTCTGGTTTCGTCATACCTTTTGGCCGCTTTCATCAGTCCGAGGTTGCCTTCATTAATCAGATCCGGAAGCGAAAGCCCCTGGTTTTGGTATTGCTTGGATACAGACACTACGAAACGAAGGTTGGCTTTGATGAGTTTTTCCAGTGCGGCTCTGTCGCCGGCGCGGATTCTTTGTGCCAGATCTACTTCTTCGTCTGCAGTAATCAGTTCCACTTTACCGATTTCCTGAAGATACTTGTCAAGCGATGCGGTTTCCCTGTTGGTAACCTGTTTTGTGATTTTTAACTGTCTCAT

>Chryseobacterium_lacus_YLOS41T

AGTATTCAGGCACTGGAAGGAATGGAGCACGTTCGTATGCGGCCTTCCATGTACATAGGTGATGTAGGTGTAAGAGGTCTGCACCATTTAGTTTATGAAGTGGTGGATAACTCTATCGATGAGGCTTTGGCAGGACACTGCGACACCATTACAGTAACTATTAAGGAGAATAATGCCATAGAAGTTACCGATAACGGACGGGGTATTCCGGTAGATTTTCACGAAAAAGAGCAAAAATCTGCATTGGAAGTCGTTATGACGAAAATTGGAGCAGGTGGGAAATTCGATAAAGATTCTTATAAAGTATCCGGCGGACTTCACGGTGTTGGTGTGTCTGTCGTTAATGCACTTTCTACAGAAATGGTCACCACAGTGTATCGTGACGGCCACGTTTATCAGCAAATGTACTCACGCGGAAAAGCCAAGGCTGATGTGGAAAAAATTGGCGATAGCGATAAAA-AAGGAACTCAACAGTACTTTCAGCCGGATGATACTATCTTTACGGAA---CTTGTGTATAATTATGATACCCTGGCCAGCAGAATGCGCGAGCTGTCTTATCTTAACAAAGGGATCACCATTACTTTAACCGATGAAAGGGAACGGAATGAAGACGAAACTTTTAAATCTGAAACATTTTATTCCGAAGGCGGACTTAAAGAATTTGTAGAATATATTGATGGAAACCGCGAGGCCATTATGGACAGCGTGATTTTCATGGAAGGTGAACGTGAAGGAATTCCCGTGGAAGTGGCGATGCGTTACAACACTTCCTTCAACGAGAATCTGCATTCTTATGTTAATAATATTAATACCCATGAAGGCGGAACACACTTGGCTGGTTTCAGAAGAGCTTTAACCAGAACTTTAAAGAAATTCGCAGATGAATTGGGACTTCCTGCCAAAGAAAAAGTAGAAGTTACGGGTGATGATTTCCGGGAAGGATTGACGGCTGTAATTTCCGTGAAAGTGATGGAGCCTCAGTTTGAGGGGCAAACCAAAACAAAACTCGGAAACTCTGAGGTTTCAGGAGCAGTGGATAAAATAGTAGGCGAGATGCTGACCAACTTTCTGGAAGAACATCCCAACGAAGCTAAACTGATTGTTCAGAAAGTAGTATTAGCCGCAAAAGCCCGACAGGCTGCCAAGAAAGCCAGAGAAATGGTACAGCGCAAATCGCCGATGGGCGGTGCGGGGCTTCCCGGTAAATTGGCCGACTGTTCTTCCAAAGATCCTGAAATTTCAGAACTATTTCTGGTAGAGGGGGATTCCGCCGGCGGAACAGCGAAACAAGGCCGCGACCGACACTTTCAGGCCATCCTTCCCTTGAGAGGTAAAATTCTGAATGTGGAAAAATCCATGCTTCATAAAGTGTATGAAAACGAAGAAATCAAAAATATCTATACGGCACTCGGCGTTTCCATAGGAACTGAAGAAGATAGCAAGGCTTTGAATATTAGCAAATTAAGATACCATAAAGTAGTAATTATGACAGATGCTGATATTGACGGATCTCACATTTCCACCTTGATTCTTACTTTCTTTTTCAGATATATGAAAGAATTGATTGAACAGGGTTACATTTATATCGCTTCTCCGCCGCTTTATCTTTTGAAAAAAGGAAATAAAAAAGTGTATGCTTGGAACGAAAAAGAAAGAGAAGAAAAAACGCTGGAAATGTCTGCTGATGGCAAAGGAGTAGAAGTTCAGCGGTACAAAGGTCTTGGGGAAATGAATGCAGAACAGCTTTGGGAAACCACCCTGAACCCAGATAACCGAACTTTGAAGCAAGTTACTATCGAAAGCTTGGCAGATGCAGATAATGTATTTTCGATGCTGATGGGAGATGAGGTTCCGCCAAGAAGGGATTTTATTGAGAAGAATGCCCGCTACGCTAGAATTGATGAATTTTCTATTATCCAGAATACATTACAAAAAGATCCTACAAACTCTGAAAAAGAGGCTGTGGAATATATCTACCGTCAGCTGAGAAATGCAGATCCGCCAGATGAGGAAACGGCAAGAGGAATTATTGAGAAATTATTCTTTTCTGAACAAAGATATTCATTAGGTGAGGTTGGGCGTTACCGTCTGAACAAAAAATTAGGATTAAATATCCCTGAAACCACAGAAGTTCTAACCAAGGACGACATCATCGCTATTGTTCGCCACCTTATTGAGTTGGCCAACTCTAAAGCGGAGGTGGACGATATTGACCATCTTTCTAACAGAAGGATTAAAACAGTTGGTGAGCAGTTGGCAGGACAATTCGGCGTAGGTCTTTCCAGAATTGCAAGAACAATTCGCGAGAGAATGAATGTGCGTGATAACGAGATCTTTACGCCAGTTGATTTGGTGAATGCGAAGACATTAACCTCCGTAATCAACTCTTTCTTTGGTACCAATCAGCTTTCTCAGTTTATGGACCAAACGAACCCCTTGTCGGAAATCACACATAAGAGAAGATTGTCGGCTTTAGGGCCTGGTGGCCTTTCAAGAGAAAGAGCAGGTTTCGAGGTTCGAGATGTTCACCATACGCACTACGGAAGAATCTGTCCGATTGAAACTCCGGAAGGACCGAACATCGGTTTGATTTCCTCGTTAGGTATCTATGCGAAAATCAACAACTTAGGGTTTATTGAAACACCCTACAGAAAAGTAGAGAACGGAAAAGTAGATCTGAAGGCGAAACCGATATATCTGAATGCAGAAGATGAAGAGGATAAGGTTATCGCACAAGCTAACGTTGACCTGAACGAAGACGGAGTGTTCGTAACAGACAGAATTATTGCCCGTTTGGATGGTGATTATCCTGTAGTTGAACCTCAGCAGGTAGACCTTATCGATATTGCTCCAAATCAGATTTCCGGTATTTCTGCATCCTTGATTCCGTTTTTGGAACATGACGATGCGAACCGTGCCTTGATGGGATCCAACATGATGCGTCAGGCAGTGCCATTATTGATGCCGGATGCGCCGATTGTAGGAACAGGTCTTGAAAAACAAGTAGCAAAAGATTCCAGAATTCTGATTAATGCAGAAGGATCCGGAATTGTAGAATATGTAGATGCTGATAAAATTGTTATTAAATATGACAGAAGTGAAGATGAAGACATTGTAAGCTTCGAATCAGCAACTAAAACTTATAATCTAACCAAATTCAGAAAAACCAACCAAGGTACAACCATCACTTTGAGGCCAAACGTAAGAGTAGGCAATAAAGTTCATGAAGGGCAGGTACTTTGCGACGGTTATGCAACTGAAGATGGAGAGTTGGCGTTGGGTAGAAACCTTACCGTAGCATTCATGCCTTGGAAAGGATATAACTTTGAGGATGCCATCGTAATTAACGAAAAAGTGGTTCGTGAGGACTGGTTTACGTCGATTCACGTAGATGAATATTCTCTTGAAGTTCGTGATACCAAATTAGGAATGGAAGAACTCACAGCAGATATCCCGAATATCTCTGAAGAGGCTACCAAAGACCTTGATGAAAACGGTATGATTAGAATTGGAGCCGACGTGAAGCCTGGCGACATTATGATCGGTAAAATTACGCCTAAAGGTGAATCTGATCCTACACCGGAAGAAAAATTACTGAGAGCGATCTTTGGTGATAAAGCCGGCGATGTGAAAGATGCTTCCCTGAAAGCAGATTCTTCATTAAGAGGAGTGGTGATCAACAAAAAGCTGTTCTCTAGAAATATCAAGGATAAAAAGAAAAGAGCGGAAGAAAAACTTAAATTGGAAGAAATCGAAAACGACTACAAAGCAAAATTCGATGATTTAAGAAATACGCTGATCGAAAAACTTGGAACTTTGGTTAATGGGAAAACTTCACAAGGCGTGAATAACGATTTGAGTGAAGAAATCATTGGAAAAGGAGTTAAATTTTCTACCAAATTGTTACAAAGTGTTGAAGATTATGTGAATGTTAGCGGTTCCGACTGGACGGTTGATAATGACAAGAATAATCTTATCAAACAATTGATTCATAACTATAAAATCAAGTATAATGACATTGCCGGTGTGAAAAACAGAGAGAAATTTGCTGTTTCCATCGGGGATGAACTTCCTGCAGGAATCATGAAACTTGCCAAAGTTTATGTCGCTAAAAAACGTAAACTGAACGTTGGTGATAAAATGGCAGGGCGTCACGGTAACAAAGGTATTGTTTCACGTATTGTTCGTGAAGAAGATATGCCGTTCCTGGAAGACGGAACACCAGTAGATATCATTTTGAATCCACTAGGCGTACCTTCCCGTATGAACATCGGACAGATTTATGAAACTGTTCTTGGATGGGCCGGTCAAAAACTGGGTCTTAAGTTTGCTACTCCGATTTTCGACGGTGCATCATTGGATGACATCACGAAATATACGGATGAAGCAGGTTTGCCAAGATACGGAAGTACCTATCTGTACGACGGTGGAACCGGTGAAAGATTCACACAACCGGCAACTGTAGGTGTGATTTATATGTTGAAATTAGGCCACATGGTGGATGACAAGATGCACGCACGTTCCATCGGTCCTTATTCATTGATTACGCAGCAACCACTCGGTGGTAAAGCGCAATTCGGTGGACAGCGTTTCGGGGAGATGGAAGTTTGGGCTCTGGAAGCATTTGGTGCATCCAACATCCTGAGAGAAATCTTAACCGTGAAGTCTGATGACGTGATTGGTAGAGCGAAAACTTATGAAGCCATCGCCAAAGGTGAAGCAATGCCAGAACCTGGAATTCCTGAATCTTTCAACGTATTGTTGCACGAACTGCAGGGTCTTGGTCTGGATGTGAGAATTTTACTTCTCGTATTGTGTTTCAGCCTTTTGATCGCTTTTTCTTTAATCTGGCGAACGCGCTCACGGGTGAGGTCGAAGGTTTCTCCGATTTCTTCCAGCGTCATCGGATGCTTTCCGTTCAGTCCGAAATAGAGGCGCACCAAATCCGCTTCTCTTTGGGTCAGCGTTTGCAGTGCTCTTTCAATTTCAATCTGAAGAGATTCCAGCATCAAGTCCTTATCCGGGCTTGGCGATTCGCCGGAACGCAGCACATCGTATAAATTGGAATCTTCACCTTCTACCAGTGGGGCATCCATCGAAAGGTGACGGCCGCTGTTTTTCATGGATTCCTTGATGTCTTCTTCGCTCATATCCAGCACTTCTGCCAGTTCTTCCGGGGAAGGAGGTCTTTCGTTTTCCTGCTCCAGGTGGGCGTACGCTTTGTTGATTTTATTGATGGAACCAATTTTGTTCAAAGGAAGCCTTACAATTCTCGACTGTTCTGCAAGGGCCTGAAGAATACTCTGCCTGATCCACCATACGGCGTAGGAAATAAACTTAAAACCTCTGGTTTCGTCATACCTTTTGGCCGCTTTCATCAGTCCGAGGTTGCCTTCATTAATCAGGTCCGGAAGCGACAATCCCTGGTTTTGGTATTGCTTGGATACAGACACTACGAAACGAAGGTTGGCTTTGATGAGTTTTTCCAGTGCGGCCCTGTCGCCGGCGCGGATTCTTTGTGCCAGATCTACTTCTTCGTCTGCAGTAATCAGTTCCACTTTACCGATTTCCTGAAGATACTTGTCAAGCGATGCGGTTTCCCTGTTGGTAACCTGTTTTGTGATTTTTAACTGTCTCAT
