# Supplementary material for: Chryseobacterium lacus sp. nov. Isolated From the Surface Water of Two Lakes With Light-Induced Carotenoid Production
Source: Front Microbiol. 2020 Mar 4;11:251. doi: 10.3389/fmicb.2020.00251 (PMC7064467; doi:10.3389/fmicb.2020.00251)
Supplement: Supplementary file 2 [file Data_Sheet_2.docx]

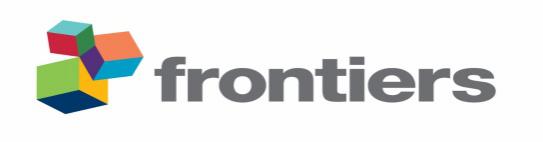


Supplementary Material

# *Chryseobacterium* *lacus* sp. nov. with light-induced carotenoid production isolated from the surface water of two lakes in Yunnan Province

**Jing Zhang ^1, 2^,** **Cheng Gao^1^, Xue-Mei Yu ^1^, He-Yuan Lun ^1^, Zong-Jun Du ^1, 2,^ ***

^1^ Marine College, Shandong University, Weihai, Shandong, 264209, China

^2^ State key Laboratory of Microbial Technology, Shandong University, Qingdao, Shandong, 266237, China

*** Correspondence:**

Corresponding Author

[duzongjun@sdu.edu.cn](mailto:duzongjun@sdu.edu.cn)

Table S1. Accession numbers of genomes and sequence similarity (%) of three housekeeping genes with *C. lacus* sp. nov. YLOS41^T^ in MLSA analysis.

| Species | Accession no. | Sequence similarity (%) with *C. lacus* sp. nov. YLOS41^T^ | | |
| --- | --- | --- | --- | --- |
|  |  | *gyrB* | *rpoB* | *RpoD* |
| 1 | RWJH00000000 | * | * | * |
| 2 | RWJG00000000 | 86.32 | 98.27 | 97.39 |
| 3 | CP034171 | 81.15 | 83.73 | 85.39 |
| 4 | FNBH00000000 | 78.79 | 80.62 | 81.24 |
| 5 | QNUG00000000 | 78.63 | 80.20 | 80.40 |
| 6 | JASZ00000000 | 78.36 | 82.63 | 81.71 |
| 7 | PPEH00000000 | 78.35 | 81.78 | 80.40 |
| 8 | FTPU00000000 | 78.24 | 80.44 | 82.78 |
| 9 | FNWX00000000 | 78.13 | 81.17 | 81.35 |
| 10 | LJOD00000000 | 78.05 | 81.46 | 80.76 |
| 11 | AUFK00000000 | 77.77 | 81.24 | 81.35 |
| 12 | LR134289 | 77.71 | 81.17 | 81.00 |
| 13 | LR134503 | 77.53 | 80.64 | 81.24 |
| 14 | FRAM00000000 | 77.15 | 80.68 | 81.59 |
| 15 | LFNG00000000 | 76.85 | 82.29 | 82.54 |
| 16 | FNUS00000000 | 75.37 | 77.64 | 78.27 |
| 17 | [FQZH00000000](https://www.ncbi.nlm.nih.gov/nuccore/FQZH00000000.1) | * | * | * |

Strains: 1, *C.* *lacus* sp. nov. YLOS41^T^; 2, *C.* *lacus* XH07; 3, *C. taklimakanense* CCTCC AB 208154^T^; 4, *C. hungaricum* DSM 19684^T^; 5, *C. hispanicum* KCTC 22104^T^; 6, *C. haifense* DSM 19056^T^; 7, *C. lactis* NCTC11390^T^; 8, *C. bovis* DSM 19482^T^; 9, *C. hominis* DSM 19326^T^; 10, *C. indologenes* NBRC 14944^T^; 11, *C. caeni* DSM 17710^T^; 12, C. gleum JCM 2410^T^; 13, *C. jeonii* NCTC13459^T^; 14, *C. molle* DSM 18016^T^; 15, *C. koreense* CCUG 49689^T^; 16, *C. humi* DSM 21580^T^; 17, *Flavobacterium haoranii* DSM 22807^T^.

Table S2. Cellular fatty acid composition (%) of the novel strains and related species.

| Fatty acid | 1 | 2 | 3 | 4 |
| --- | --- | --- | --- | --- |
| Straight-chain fatty acids |  |  |  |  |
| C_16:0_ | 0.8 | 3.7 | - | 3.1 |
| C_18:0_ | 0.5 | 0.6 | - | 1.2 |
| Unsaturated fatty acids |  |  |  |  |
| anteiso-C_17:1_ *ω*9*c* | 1.8 | - | - | - |
| C_18:1_*ω*9*c* | - | 0.6 | 0.7 | 1.4 |
| Branched fatty acids |  |  |  |  |
| iso-C_13:0_ | 1.7 | 1.3 | 0.7 | 1.4 |
| iso-C_14:0_ | 2.8 | 1.7 | 1.4 | - |
| iso-C_15:0_ | **40.5** | **32.8** | **33.9** | **37.2** |
| iso-C_16:0_ | 5.5 | 2.8 | 5.2 | - |
| iso-C_16:1_ H | 1.6 | 0.8 | 0.9 | - |
| iso-C_17:0_ | 1.3 | - | - | 4.1 |
| anteiso-C_15:0_ | **14.5** | **19.4** | **20.6** | 0.7 |
| anteiso-C_17:0_ | 0.5 | - | - | - |
| Hydroxy fatty acids |  |  |  |  |
| C_15:0_ 2-OH | 0.6 | 0.9 | 1.2 | - |
| iso-C_15:0_ 3-OH | 1.8 | 2.1 | 2.3 | 3.6 |
| iso-C_16:0_ 3-OH | 4.5 | 3.2 | 8.2 | 1.0 |
| iso-C_17:0_ 3-OH | **11.4** | 8.4 | 9.1 | **22.0** |
| Summed Feature ^a^ |  |  |  |  |
| 3 | 1.2 | 8.7 | 1.1 | 7.1 |
| 4 | - | - | 1.5 | - |
| 8 | - | 2.8 | - | 0.8 |
| 9 | 6.8 | 7.6 | 7.5 | **16.6** |

Strains: 1, *C.* *lacus* sp. nov. YLOS41^T^; 2, *C.* *lacus* XH07; 3, *C. taklimakanense* CCTCC AB 208154^T^; 4, *C. gleum* JCM 2410^T^. All data listed in the table are from this study.

–, trace quantities (< 0.5%); Fatty acids present at >10% are indicated in bold;

^a^ Summed Features represent groups of two or three fatty acids that could not be separated by GLC with the MIDI system. Summed Features 3 consisted of C_16:1_ *ω*7*c*/C_16:1_ *ω*6*c*, Summed Features 4 consisted of iso-C_17:1_ I/anteiso- C_17:1_ B，Summed Features 8 consisted of C_18:1_ *ω*7*c*/C_18:1_ *ω*6*c*，and Summed Features 8 consisted of 10-methyl C_16:0_/iso-C_17:1_ *ω*9*c*.

Table S3. ANIb, ANIm and *is*DDH value (%) between *C. lacus* sp. nov. and its closely related species.

| Species | *C.* *lacus* YLOS41^T^ | | | | *C.* *lacus* XH07 | | |
| --- | --- | --- | --- | --- | --- | --- | --- |
|  | ANIb | ANIm | *is*DDH | ANIb | | ANIm | *is*DDH |
| 1 | * | * | * | 96.52 | | 96.99 | 72.5 |
| 2 | 96.47 | 96.98 | 72.5 | * | | * | * |
| 3 | 73.06 | 85.06 | 20.9 | 73.04 | | 85.28 | 20.8 |
| 4 | 70.92 | 84.44 | 17.5 | 70.84 | | 85.00 | 18.1 |
| 5 | 71.13 | 86.35 | 20.5 | 71.10 | | 86.42 | 20.8 |
| 6 | 70.54 | 86.05 | 20.6 | 70.53 | | 86.26 | 20.3 |
| 7 | 72.84 | 86.61 | 19.6 | 72.69 | | 86.36 | 19.5 |
| 8 | 71.18 | 86.76 | 22.0 | 71.04 | | 86.41 | 22.9 |
| 9 | 71.03 | 84.48 | 19.4 | 70.97 | | 84.54 | 18.8 |
| 10 | 70.79 | 84.93 | 18.6 | 70.71 | | 85.23 | 18.7 |
| 11 | 71.27 | 83.53 | 17.9 | 71.26 | | 84.08 | 17.9 |
| 12 | 71.45 | 84.20 | 19.0 | 71.49 | | 84.69 | 18.7 |
| 13 | 71.90 | 84.77 | 19.5 | 72.04 | | 85.71 | 20.3 |
| 14 | 70.85 | 86.36 | 21.1 | 70.77 | | 86.84 | 22.4 |
| 15 | 70.38 | 84.36 | 18.5 | 70.45 | | 84.82 | 18.6 |
| 16 | 70.46 | 83.90 | 19.8 | 70.54 | | 84.69 | 20.2 |

Strains: 1, *C.* *lacus* sp. nov. YLOS41^T^; 2, *C.* *lacus* XH07; 3, *C. taklimakanense* CCTCC AB 208154^T^; 4, *C. gleum* JCM 2410^T^; 5, *C. lactis* NCTC11390^T^; 6, *C. bovis* DSM 19482^T^; 7, *C. haifense* DSM 19056^T^; 8, *C. hispanicum* KCTC 22104^T^; 9, *C. hominis* DSM 19326^T^; 10, C. humi DSM 21580^T^; 11, *C. indologenes* CI_885^T^; 12, *C. jeonii* NCTC13459^T^; 13, *C. koreense* CCUG 49689^T^; 14, *C. mole* DSM 18016^T^; 15, *C. caeni* DSM 17710^T^; 16, *C. hungaricum* DSM 19684^T^.

Table S4. Significantly differentially expressed genes of strain YLOS41^T^ under light vs. dark condition. Genes with adjusted P < 0.05, regardless of the fold‐change.

| locus_tag | log2Fold-change (light vs. dark) | padj | product | cellular role |
| --- | --- | --- | --- | --- |
| RS02775 | 0.57527 | 1.69E-03 | formate--tetrahydrofolate ligase | Unknown |
| RS05385 | 0.5989 | 8.28E-04 | elongation factor G | Unknown |
| RS06185 | 2.3992 | 5.38E-43 | Outer membrane lipoprotein | Unknown |
| RS06190 | 2.0754 | 1.74E-28 | lycopene cyclase CarR_dom_SF | Crt biosynthesis |
| RS06195 | 1.7367 | 1.60E-20 | beta-carotene hydroxylase | Crt biosynthesis |
| RS06200 | 1.579 | 1.90E-13 | hypothetical protein | Unknown |
| RS06205 | 1.5605 | 2.72E-28 | phytoene synthase protein | Crt biosynthesis |
| RS06210 | 0.94333 | 2.18E-10 | phytoene desaturase | Crt biosynthesis |
| RS02480 | -0.57373 | 3.06E-02 | hypothetical protein | Unknown |
| RS03965 | -0.58598 | 3.06E-02 | peptide chain release factor 1 | Unknown |
| RS10695 | -0.59247 | 7.64E-03 | DEAD/DEAH box helicase | Unknown |

**
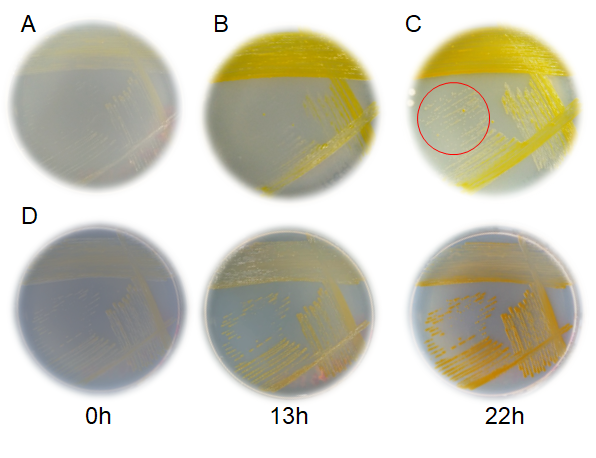
**

Figure S1. Colony color of the strain YLOS41 cultured for 15 hours in light (A) or darkness (B), the strain cultured for 15 hours in light continued to culture for 8 hours in dark (C), and the strain cultured for 20 hours in dark continued to expose to light irradiation for 0 to 22 hours (D). The colonies in the red circle are new colonies in dark.


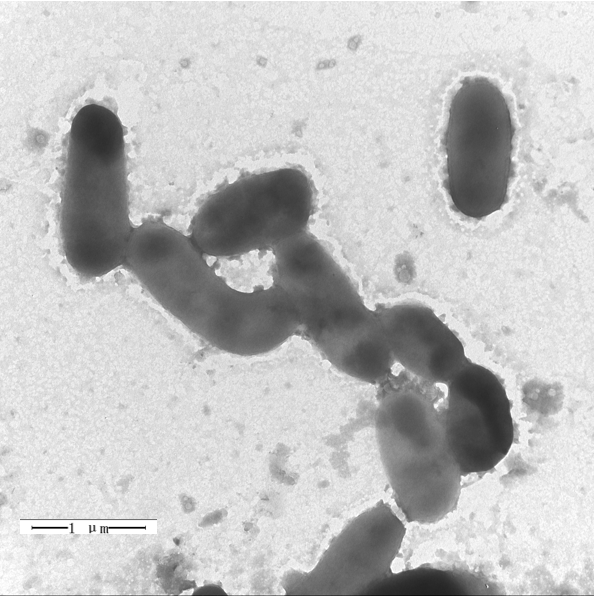

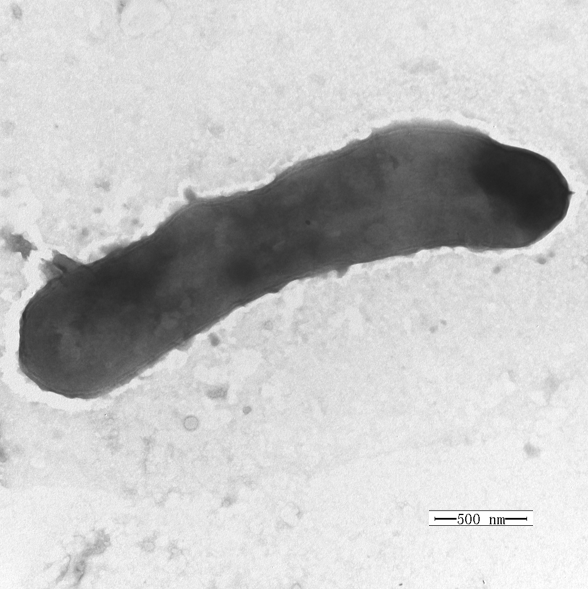


1. (b)

Figure S2. Transmission electron micrograph of cells of *C.* *lacus* sp. nov. YLOS41^T^ (a) and *C.* *lacus* XH07 (b) after growth on modified agar 2216 at 33 °C for 24h.


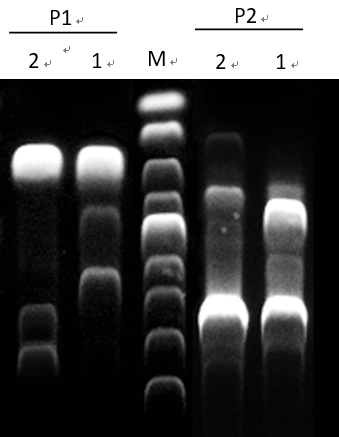


2000 bp

5000 bp

3000 bp

1500 bp

1000 bp

750 bp

100 bp

250 bp

500 bp

Figure S3. RAPD-PCR profiles of novel strains *Chryseobacterium* *lacus* YLOS41^T^ (lanes 1) and XH07 (lanes 2). Two primers with random sequences (P1, 5’-CTGCTGGGAC-3’ and P2, 5’-CGCCCTGCCC-3’) were used. Lane M, 5000-bp DNA size marker.

A


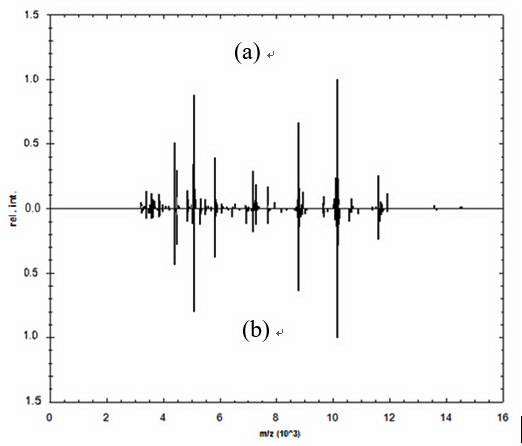


B


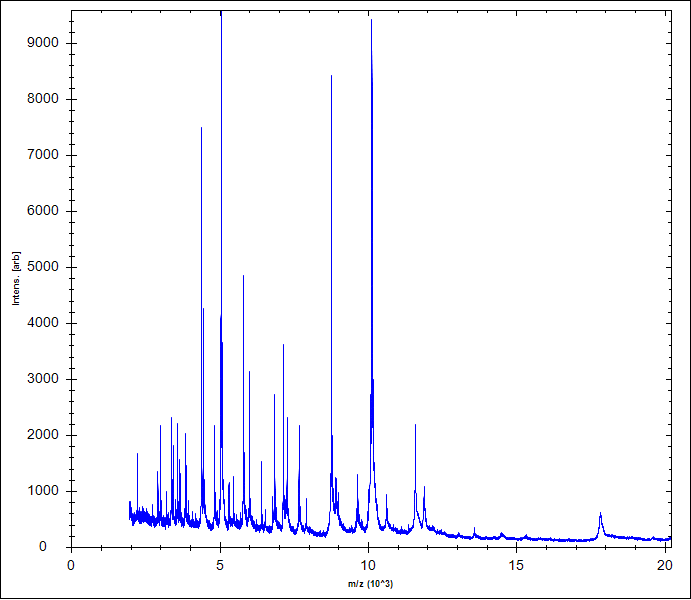

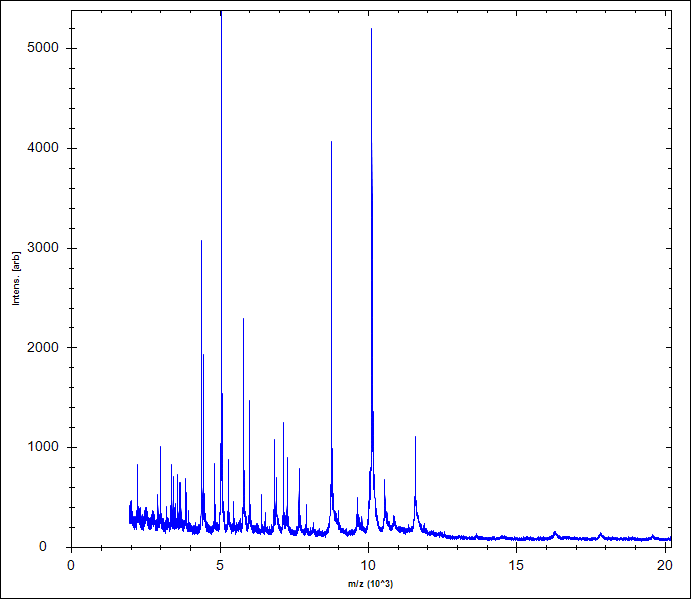


1. (b)

Figure S4. MALDI-TOF MS spectrogram of cell lysates of *C.* *lacus* sp. nov. YLOS41^T^ (a) and *C.* *lacus* XH07 (b). A, comparison spectrogram; B, original spectrogram.


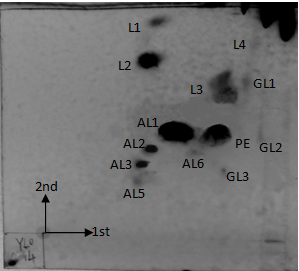

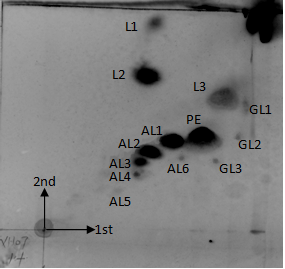


1. (b)


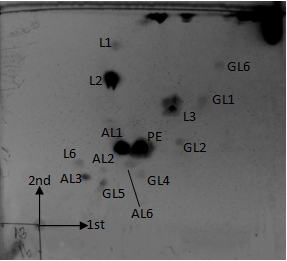

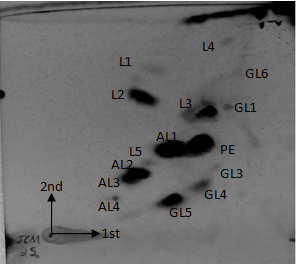


(c) (d)

Figure S5. Two-dimensional TLC of total lipids of *C.* *lacus* sp. nov. YLOS41^T^ (a), *C.* *lacus* XH07 (b), *C. taklimakanense* CCTCC AB 208154^T^ (c), *C. gleum* JCM 2410^T^ (d), stained with 10% molybdatophosphoric acid. PE, phosphatidylethanolamine; AL, aminolipid; GL, glycolipid; L, lipid.


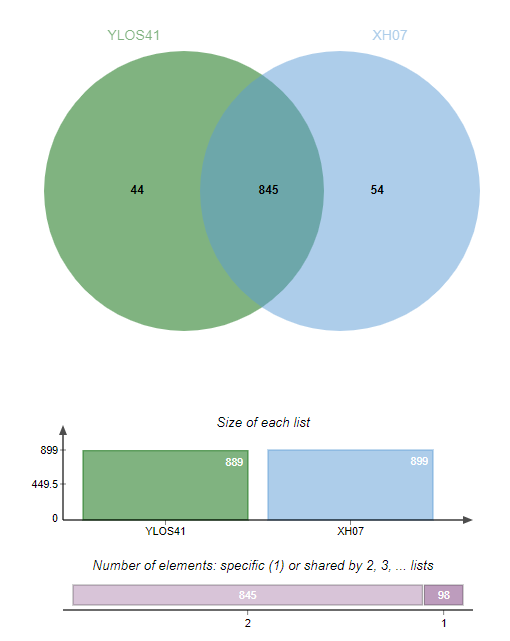


Figure S6. Venn diagram of annotation by KEGG of *C.* *lacus* sp. nov. YLOS41^T^ and *C.* *lacus* XH07.


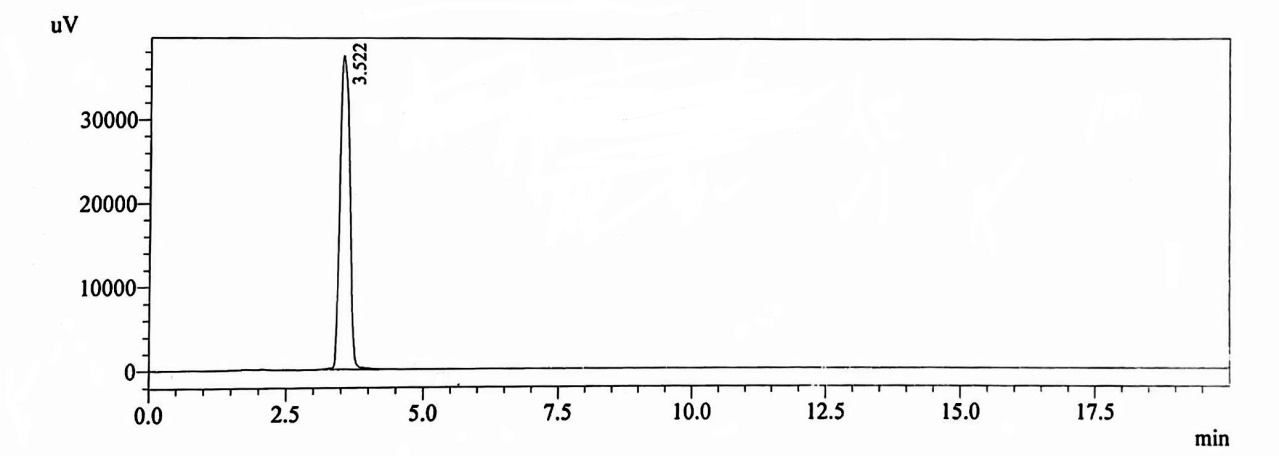

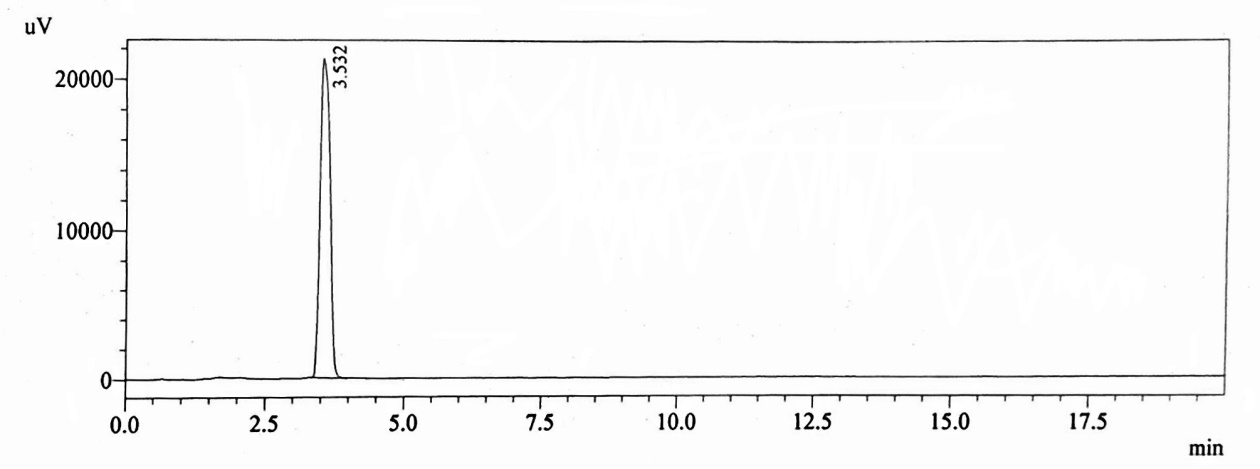


(a)

(b)

Figure S7. High-pressure liquid chromatography (Shimadzu HPLC LC-20AT) spectra of carotenoids extracted from strain YLOS41^T^ (a) and the standard of zeaxanthin (b).

The tests were by C18 column with a mobile phase of acetonitrile-water (volume ratio being 88:12). UV detection was performed at 254 nm.
